# A qualitative study of oral health knowledge among African Americans

**DOI:** 10.1371/journal.pone.0219426

**Published:** 2019-07-10

**Authors:** Sherieda Muthra, Rhonda Hamilton, Katherine Leopold, Everett Dodson, Dale Mooney, Sherrie Flynt Wallington, Chiranjeev Dash, Lucile L. Adams-Campbell

**Affiliations:** Georgetown University Lombardi Comprehensive Cancer Center, Washington D.C., United States of America; University Lyon 1 Faculty of Dental Medicine, FRANCE

## Abstract

**Objectives:**

The purpose of this qualitative oral health needs assessment was to probe and better understand the oral health knowledge, beliefs, and barriers of District residents, particularly in DC wards where oral health disparities are most prevalent.

**Methods:**

Forty-eight (n = 48) participants were recruited for four focus groups. The focus group instrument consisted of a structured interview guide addressing the following topics: oral health history, perceived barriers to oral health, knowledge and perceptions about oral systemic health, and preferred message channels for receiving information on oral/dental health. Content analysis was performed using NVivo, a computerized, qualitative informatics tool.

**Results:**

The majority of participants in this study practiced both brushing and flossing in their daily dental routine and did not believe that tooth loss is a normal part of ageing. There was lack of knowledge on the connection between oral and systemic health, specifically impact of smoking, alcohol use, and sweets and sexual activity. Focus groups identified two main barriers to healthcare access–communication and affordability. Participants who had a dentist were satisfied and felt that their needs were met.

**Conclusion:**

Our findings indicate a need for educational intervention and improved communication from oral health providers to increase awareness of the impact of systemic health and risky behaviors can have on oral health.

## Introduction

Despite advances in oral health status and disease prevention in the United States over the past four decades, comparatively low rates of improvement have been achieved among socioeconomically disadvantaged and select racial/ethnic minority populations [1–3]. Though historically treated as an isolated aspect of health, it is now known that oral health contributes to and is affected by overall systemic health. Poor oral health has been associated with cancer, inflammatory disease, cardiovascular disease, diabetes, adverse pregnancy outcomes, and osteoporosis [4–9]. Research has also shown that most systemic diseases have oral manifestations, such that oral health can serve as an indicator of general health [10].

Disparate oral disease burden is more prevalent in socioeconomically disadvantaged and minority populations, yet the reasons for these disparities are poorly understood. Studies exploring oral health disparities have found that socioeconomic factors of income and education play a significant role in the imbalance that exists [11–13]. Lower income and education levels often correlate with worse oral health status and general health. In a study of National Health and Nutrition Examination Survey III (NHANES III) data focusing exclusively on 5,616 African Americans, 62% of study participants indicated that they did not have regular dental care and only visited a dental professional when needed. NHANES III data also revealed that dental health was worse for study participants who were poor, unemployed, and/or uninsured, particularly for those living in southern states [14].

According to the 2017 SocioNeeds Index for medically underserved areas in Washington, DC, rank highest with regard to socioeconomic need, which is correlated with poor health outcomes [15]. African Americans in underserved areas of the District are disproportionately affected by high incidence rates of untreated dental caries, gingivitis, periodontal disease, and oral cancer compared to non-Hispanic Whites [16]. However, scant data are available on oral health access among District residents.

The purpose of this qualitative oral health needs assessment was to probe and better understand the oral health knowledge, beliefs, and barriers of District residents, particularly in DC wards where oral health disparities are most prevalent. The data obtained in this study may serve as a guide for improved oral health care in communities with the greatest need.

Other major barriers to receiving oral health care that exist among underserved African American adults include limited access to dental insurance and prohibitively high costs [17,18]. African American men in low-income, urban communities that have difficulty finding dental care are 4.81 times more likely to report no dental insurance [18]. Older African Americans report that transportation to and from dental resources can prevent access to dental care [17]. These data show that several African American constituencies, when making the decision to seek oral health care, face significant personal and social hurdles.

## Methods

### Sample and setting

The target population for this study was African American men and women residing in underserved areas of Washington, DC. Eligibility criteria for participation included the following: (a) being at least 18 years of age; (b) residing in Washington, DC; and (c) being able to speak and understand English. Recruitment strategies entailed distribution of recruitment flyers to key points of contact at public venues and community-based organizations, including churches, community centers, health fairs, and public housing by our community outreach and recruitment staff. The Georgetown-Lombardi Community Advisory Board, which consists of community residents and representatives of community-based organizations, also provided assistance with targeted recruitment. The focus groups were convened at the Georgetown-Lombardi community-based office, churches, and public housing communities. The participants were identified and recruited between January and March 2016. A total of 74 men and women were screened and 16 individuals were ineligible. The men and women were ineligible due to: (1) age, n = 11); and not resident of DC (n = 5). In addition there were 10 no shows. This study was funded by the Richmond Foundation and approved by the Georgetown University Medical Center Institutional Review Board.

### Focus groups

Focus groups were chosen as the qualitative data collection method for this study. Forty-eight (n = 48) participants were recruited for four focus groups with 9–14 participants per group. Each participant provided written informed consent for the IRB approved study. Data were obtained through self-report by the participants. The focus group instrument consisted of a structured interview guide. The interview guide was developed based on Morgan’s funnel strategy [19] design (see Focus Group Instrument, Supplement 1) in order to explore the experiences, views, and insight of participants. The focus groups were undertaken until saturation, meaning no new information of value emerged [19]. The duration of each group was approximately 1 hour and 30 minutes. Two focus group moderators trained in qualitative research facilitated the focus groups and asked questions across the following topics: oral health history, perceived barriers to oral health, knowledge and perceptions about oral systemic health, and preferred message channels for receiving information on oral/dental health. Lunch and a small incentive ($25 gift card to a local grocery store or pharmacy) were provided. Transcription of audio recordings was analyzed for themes and key points.

### Data analysis

Focus group sessions were digitally recorded and transcribed by Precision Consulting LLC in Wilmington, Delaware. All data from focus groups were transformed into codable units for analysis. Systematic transcript analysis involved detailed reading and group discussion of the full transcripts by coders, inclusive of content analysis. Analysis was carried out using a constant comparative method. Participant responses to focus group questions were categorized based on the focus group interview guide. Emergent themes from the data were determined through the use of codes. Thematic categories were developed from each question and all participant responses were considered to be thematic elements. Themes were determined by the frequency of responses referencing a given thematic element. Content analysis was performed using NVivo, a computerized, qualitative informatics tool.

## Results

A total of 48 individuals (40 females and 8 males, all African American) participated in four focus groups. Each of the four focus groups consisted of Washington, DC, residents from medically underserved areas. The overarching focus group themes that emerged based on analysis were (a) oral health knowledge, (b) behaviors, (c) attitudes, (d) barriers, and (e) needs. There was primarily a lack of knowledge regarding the relationship between disease and oral health. Most participants (60.4%) did not know about the connection between chronic disease and oral health and did not recall ever hearing about it. Few participants (15%) had discussed the link between oral health and diseases such as HPV, cardiovascular disease, and diabetes with their oral health provider. Among the fifteen participants who discussed the connection between oral health and tooth loss with aging, most participants (67%) knew that losing teeth was not a normal part of getting older but 20% thought it was a normal part of getting older. One participant explained, “From what I’ve heard, if you had good dental care it shouldn’t have anything to do with age.” Another participant noted, “I lost most of my teeth…the ones that are gone…in my younger years, so I don’t think it’s [a part of] getting older.” There were participants who were unsure if there was a correlation between aging and losing teeth, with one participant stating, “It seems like everyone that’s older than me always have fake teeth. I don’t know.” Another respondent said, “Everything is because of getting older.”

Oral health and hygiene was characterized by the participants based on: having regular visits to the dentist (64%); daily brushing (50%); daily flossing (20%); and to take care of health and gums (75%). Two participants added that good oral hygiene is about “your complete dental care.” Interestingly, one participant noted that it is “taking care of the total body.” While most participants demonstrated knowledge regarding good oral hygiene, two participants shared that they believed flossing between teeth was unnecessary because they always brush their teeth very well.

Oral health behaviors, comprising three subthemes: food, beverages, and bad habits, were revealed by the participants that the following contributed to poor oral health: 30% sweets; 20% alcohol; and 20% smoking. One respondent noted, “Cigarettes and tobacco especially will contribute to poor oral health and cancer.” Only 10% viewed sexual activity as being a behavior that could contribute to poor oral health.

Overall, participants across all four focus groups agreed that oral health was just as important and as serious as other health problems. Many participants correlated oral health with general health, noting that poor oral health causes problems with mental health, cardiovascular health, diabetes, systemic infection, and death. One participant said, “I take it very seriously because if you don’t get your teeth and stuff taken care of—I know a lot of people who passed away by having bad teeth in their mouth,” while another stated, “I think because it will stir up other things in your body; where your teeth is bad other things will go bad in your body…your heart or with different things will go bad.” These responses were characteristic of participants’ attitudes toward the importance of oral health as it pertains to their overall health.

The majority of focus group participants were insured; however, many were unsure as to whether they actually had dental coverage. Concerns were also raised that despite insurance, it is sometimes difficult to find dentists who accept their insurance and are also accepting new patients. Among the insured participants, knowledge of what services are covered by their dental insurance was mixed, with the majority of participants indicating that they did know their dental coverage and stating that services such as cleanings, x-rays, and extractions were covered. For participants that did not know their dental insurance coverage, the most common reasons presented were either that the insurance company did not inform them, coverage had recently been changed, or there was a designated family member or caregiver that handled the details regarding their medical care. All but one participant said that they knew of a place that they could go if they had a dental emergency. Participants noted that in case of a dental emergency during times when they were unable to go to a dentist, they could go to places such as the emergency room and urgent care clinics.

Many participants (91%) felt that cost is a barrier to receiving dental care. The majority of participants expressed that even with insurance, high costs often prevented people from going to the dentist. Some participants noted that with their type of insurance, they did not have to worry about the cost of dental care. One participant in particular said, “I’ve been fortunate in having a dental plan through my employer, so I spend very little money out of my pocket.” Another respondent concurred, stating, “I’ve got insurance and it covers a hundred percent of preventive maintenance for two times a year, et cetera. Anything that requires orthodontia, I have healthcare that comes out before taxes.”

In addition to cost, fear and pain are barriers that prevent participants from going to the dentist. Several participants explained that fear may be why many people do not go to the dentist. One participant shared, “The man that was cleaning my teeth, I didn’t really like how he was cleaning my teeth. It hurt. I had to fake him out like I was really having more pains to make him hurry up and finish my mouth.” Of the four participants who said fear and pain were not barriers to going to the dentist, two acknowledged that they did not like going but went anyway. As one respondent said, “I don’t like it, but I go.” Another concurred, saying, “It’s just like anything else. You’re tired of going to the doctor, but you got to go.” One of the responses to pain medication specifically said she used self-medication so that she would not have to go to the emergency room: “I wanted to say I stopped it on my own so I didn’t have to go to the emergency room, because I had fresh piece of garlic, and I just stuck it up on the side of my gum and the it knocked out the pain right away.” One female participant said she has used lemon extract to help with pain in teeth, while a tea bag to take away the pain was stated by another female. Other participants said they self-medicated with over the counter pain medication.

Participants shared that aside from needing a dentist that accepted their insurance, they desired certain qualifications, specifically communication, bedside manner, trust, cleanliness, and experience. Many participants, 37.5% indicated that communication was the greatest need when it came to dental qualifications, while 25% expressed the need for trust, and 12.5% equally expressed the need for bedside manner, cleanliness, and experience. Regarding the need for communication, participants shared comments such as “I like ones that will explain what they’re doing in detail” and “The dentists don’t seem to have a lot of time to explain things to you anymore.” Participants also noted that for the cost of dental care, they want to be told what is being done, the reasons why it is being done, and other options. Respondents overwhelming seemed to express having a greater comfort level with dentists that demonstrated good communication, and this also seemed to be associated with a higher level of trust in the dentist. Overwhelmingly, the consensus was that participants preferred to receive oral health information directly from their dentist or doctor. Many participants also expressed a desire for mixed media communications, including visual communications through television, the Internet, brochures, and focus group settings such as the one that they were currently in.

## Discussion

Although participants identified a connection between oral and general health, knowledge gaps in the context of this relationship regarding systemic disease and behaviors such as smoking/alcohol use, sexual activity, and sugar/sweets consumption remained. The deficit in knowledge of the connection between systemic disease and oral health detected here reflects the findings of previous studies in providers [20–22] and suggests the need for an educational intervention for providers and patients to expand awareness of the impact that systemic disease and risky behaviors have on oral health. Continuing education courses for oral health providers should focus on the growing body of research implicating a strong relationship between oral health and systemic disease and the importance of discussing this connection with patients during general dental visits. Providers can also screen for systemic diseases when setting up patient profiles and offer individualized advice during regular dental visits. Weak patient-provider communication could explain the lack of knowledge and fearful attitudes expressed by participants. Interventions for providers should focus on development of culturally competent provider communication skills and encourage providers to spend time explaining treatments, and expectations to patients. As public health and prevention leaders in oral health care, dental hygienists may have the time and expertise to improve provider-patient communication at regular dental visits.

### Implications for practice

Our findings suggest that educational intervention and improved communication from oral health providers may prove to be invaluable methods for reduction of oral health disparities in underserved communities.

This study included a small, self-selected group of African-Americans within medically underserved populations in Washington, D.C. The participants were mainly female (77%) and senior (60%), so findings are not generalizable, especially for males and younger adults in the population. Results of the current study provide useful insight into the oral health knowledge, barriers, and behaviors among African-Americans in underserved areas of Washington, DC. Findings indicate a need for educational intervention and improved communication from oral health providers to increase awareness of the impact that systemic disease and risky behaviors can have on oral health.

## Supporting information

S1 Text Oral Health Transcription Qualitative Analysis. This is the qualitative analysis for all of the oral health focus groups.
(DOCX)

S2 Text Oral Health Focus Group Transcription–Church 1. This is the Church 1 focus group transcription.
(DOCX)

S3 Text Oral Health Focus Group Transcription–Church 2. This is the Church 2 focus group transcription.
(DOC)

S4 Text Oral Health Focus Group Transcription–Housing. This is the Housing focus group transcription.
(DOC)

S5 Text Oral Health Focus Group Transcription–Senior Recreation Center. This is the Senior Recreation Center focus group transcription.
(DOC)
