# Supplementary material for: A qualitative study of oral health knowledge among African Americans
Source: PLoS One. 2019 Jul 10;14(7):e0219426. doi: 10.1371/journal.pone.0219426 (PMC6619789; doi:10.1371/journal.pone.0219426)
Supplement: S1 Text — This is the qualitative analysis for all of the oral health focus groups. (DOCX) [file pone.0219426.s001.docx]

**Oral Health Transcription Qualitative Analysis**

**Findings**

The following sections provide a detailed qualitative analysis of focus group results from four locations – Church, Senior Recreation Center, Housing 1, and Church 2 – addressing oral health disparities in the District of Columbia.

All data from the four focus groups was transformed into codable units for analysis. The real names of the participants were not indicated in the data analysis. Each participant had a unique pseudonym to protect his or her identity. All data was then loaded in the NVivo software and analyzed by highlighting certain texts, comparing the responses of one participant to another, compiling texts that were thematically similar, and organizing data based on emergent themes. Data was analyzed using a constant comparative analysis method. First, emergent themes from the data were determined through the use of codes. Next, the data was combined and pieced together, noting were emerging categories were and grouping similar material together. The final phase was selecting core categories and systematically relating each of these core categories to major themes. These findings address each emerging theme, how these findings correspond to the major themes across focus groups, as well as any outliers or discrepancies that emerged during analysis. Findings of this analysis are noted below for each major theme.

**Major theme 1. Knowledge**

There were three subthemes within the major theme of knowledge: oral hygiene and health, prevention and oral health, and oral care and insurance.

**oral hygiene and health.** Within the category of oral hygiene and health, there were four subcategories: knowledge about hygiene, about oral health and other diseases, oral health and ageing, and risky behaviors for oral health. Within the subcategory of hygiene, nine participants characterized their knowledge as taking care of their mouth, including brushing, flossing, and gums; two participants described their oral hygiene knowledge as regular visits to the dentists, and one said its taking care of the total body. Maria explained, “oral health care, to me, is taking care of your teeth, your mouth, your gums.” Sydney agreed, saying, “Oral health to me means brushing…taking care of your teeth, doing the things that you need to do, visiting the dentist….” Tracy concurred: “Oral health care to me is going to the dentist at least once a year to get your teeth checked, make sure you brush them every day.” She added simply, “When I hear oral health the first thing I think about is your mouth, completely. Your complete dental care.” Another respondent explained her oral hygiene knowledge as it was applied to her daily routine: “We brush our teeth in the morning, before we eat and probably…and brush our teeth like after we eat something.”

Within the subcategory of oral health and disease, there was primarily a lack of knowledge. Twelve participants noted that they had never talked with their dentist about diseases that could connect to oral health, such as HPV, cardiovascular disease, and diabetes; only seven participants said their dentist had ever even mentioned these diseases. Sarah said, “Mine didn’t…not a single one of those did a dentist ever explain about your heart, your health…your mouth, but you’re saying your heart and all those other things…cancer…no.” Sasha agreed, saying, “No. My dentist never told me anything about those.” Another respondent said her doctor had never addressed HPV: “Not at the dentist; that’s crazy. That’s a good question.” Adrienne, who said she had talked to her dentist about diseases said it’s happened “Only one time.” For another respondent, her dentist talks to her because of personal tragedy: “Because my twin had cancer and she passed away, so I’m being checked regularly for dentist and at the doctor’s.” One other respondent, Maria, said the one disease her dentist had mentioned was oral cancer. However, it was clear that this subcategory was defined by an overall lack of knowledge about these diseases.

Within the subcategory of oral health and ageing, most of participants did seem to have accurate knowledge. Ten of the participants said that losing teeth was *not* a normal part of getting older; two said it could be; and only three said that yes, losing teeth was just a part of getting older. One respondent, who noted that it was not normal to lose teeth as one ages, noted, “My father was in his 80s when he passed and he had every one of his.” Liz had a similar sentiment, saying, “I think when my grandfather died he died with all of his teeth, and he was almost 90 years old.” Sarah also agreed, saying, “From what I’ve heard that if you had good dental care it shouldn’t have anything to do with age.” Another respondent noted that one can lose one’s teeth at any age, so getting older should not be a defining factor: “I lost most of my teeth…the ones that are gone…in my younger years, so I don’t think it’s getting older.”

Sasha was unsure if there was a correlation between losing teeth and ageing, noting “it seems like everyone that’s older than me always have fake teeth. I don’t know.” Another respondent attributed teeth loss to old age, along with many other symptoms, saying “Everything is because of getting older.”

The last category in this major theme of knowledge was risky behaviors for oral health. Within this category were three subthemes – food, beverages, and bad habits. The distribution of responses for this category can be found in Table 1 below.

Table 1.

*Risky Behaviors for Oral Health*

| Themes | Number of Participants | Percentage of Participants |
| --- | --- | --- |
| Sweets | 4 | 20% |
| Smoking | 4 | 20% |
| Alcohol | 4 | 20% |
| Sugar | 2 | 10% |
| Gum | 2 | 10% |
| Soda | 2 | 10% |
| Sexual Activity | 2 | 10% |

As Maria said problems with oral health can often be attributed to “bad choices in our foods that we eat. I think that may help to contribute to poor oral health…things we drink and eat.” Four of the twenty respondents (20%) pointed the finger at sweets. As one respondent explained:

And the dentist told me sweets will ruin your teeth. And that’s probably why I still got all my teeth in my mouth right now. I eat sweets, but I eat them in spells. If I buy some cookies, next month they’re going to be in the trash, because they’re stale. I don’t deal with sweets too much. That’s why he told me I’ve still got all my teeth in my mouth. I don’t do candy or sweets.

Tiffany agreed, saying:

I eat sweets every now and then...but when I’m finished… but I don’t eat it that much. It’s only certain things I like. I eat them in spells and I’ll brush my teeth… a friend of mine back in high school, she was so young when she lost all of her teeth in high school. She just used to eat bags of candy. Not brushing her teeth, not taking care until they were rotten.

Four other respondents said that alcohol was bad for oral health, while another four cited smoking as a risky habit. One respondent noted, “cigarettes, and tobacco, especially will contribute to poor oral health and cancer” while another noted “smoking and caffeine together” were bad for one’s oral hygiene. Soda was also pointed out as a risky behavior, given its high amount of sugar and caffeine, as was sweetened chewing gum, sugar in general, and oral sex.

**prevention and oral health.** Within the category of prevention and oral health, there were two subcategories that emerged – communication about oral health and prevention and knowledge about food and beverages that helped with oral preventive care. Ten participants noted that they had received information about oral health and preventative care that was connected to other issues like oral care hygiene, smoking cessation, or dietary counseling, while only three participants said that they had never received such information. Of those ten participants, three explicitly noted that the information they had received was connected to, as Will put it, “smoking cessation.” Another respondent noted she had received information of all of the other physical care topics: “General nutrition, number one. Number Two…in fact, all of those that you named is included in a lot of health information that goes out to patients and those of us who go out and counsel or have health fairs.”

In terms of where participants received information and knowledge about oral care specifically, there were varied responses. Four participants cited social media, with three expressly pointing to Facebook. As Will explained,

The way the social media is set up today it’s very informative and it’s 24 hours a day nonstop. What I look at is structure in a lot of these programs so that at the end of the program what have I gotten out of it. When they first start there’s a question, at the end what’s the answer. I am looking for a solution. I think it’s very informative. You have multi choices.

Six participants cited television as their source of oral care information, though within that medium there were different answers. Two of those six cited Dr. Oz, one point to Oprah, and two others explicitly said advertisements were where they received information. One participants said, “You just listen to Oprah…Oprah, Oprah, Oprah,” while another noted, “On television it’s a lot. You know when they’re advertising their Sensodyne and all that kind of stuff….” Will agreed, noting “When they advertise toothbrushes, toothpaste, mouthwash. Many of the commercials have it all. It’s a regular thing. It’s constant.”

Two more participants said it was focus groups like the one they were participating in that gave them oral health information. Tracy noted it was “A group like this. You get information like this, that’s helpful.” Liz agreed, saying, “What you’ve done, what you’ve made available to the church is a good example off that…excellent example of that. That’s why I am here.” Two other participants cited magazines. Sarah specifically noted she reads “the Bottom Line magazine…and one of the articles they did a three‑page spread on oral cancer which was in depth. That was good. The reading media.” Pearl noted that private dentists advertise themselves and their services in print media as well, which acts as a source of information. Finally, one other participant pointed to the power of the Internet. Sarah said, “You don’t even have to have a computer, you can just Google on your cell phone and find an answer.”

In addition to knowledge about oral health and prevention that had been communicated, participants were able to discuss their knowledge about food and beverages that were actually helpful in oral health care. Eight participants noted that water was essential for strong oral health and five participants said milk, with its calcium, was important to oral health. Three participants pointed to vegetables and two others to fruits. One respondent noted, “Vegetables. Salads. I fix a lot of salads.” In addition, two participants cited lean protein and two cited whole grains as important for oral health. Interestingly, two participants also noted the importance of reading food and beverage labels in order to get an accurate view of what one is consuming. Sarah explained:

One thing I heard is that…I like a lot of juices and I was paying attention to the milligrams on the back. When I bought this cranberry juice that says 26 milligrams, I thought that’s not a lot, but that’s 26 milligrams per glass. What I started to do since I liked the cranberry juice, I just mix it half and half. That takes some of the…you still got a little sweet, the color, the juice. You got water and cranberry juice now, but those juices are very…got a lot of sugar in them.

Will concurred, saying, “That’s a good point. The class I took over at the hospital, they teach you how to read those labels. Reading the labels on cans and bottles is very important…per serving, not the whole bottle.”

**Oral care and insurance.** The final category in the theme of knowledge is awareness of and about oral care and insurance. All but one participant said that they had the knowledge of a place they could go to if they had a dental emergency other than the dentist’s office*.* Twelve participants noted that they could go to the hospital, including the emergency room, or a clinic. Participants listed GW, Howard University, and the Cardoza Clinic as places they could go for emergency dental care. Will also pointed out that as a Veteran, he can also go to the VA hospital for treatment. Maria noted that when DC General was still in existence, “you could always go over there for dental emergency at no cost.” Shawn was somewhat of an outlier, saying that he could always get an appointment with his dentist: “I’ve even had my dentist make an appointment for me on Sunday. He said you can come it, it will just be me and you, we can pull your tooth, take pictures, or whatever, on a Sunday. He took me.”

The final aspect of knowledge was if participants knew what was covered by their dental insurance. Eight participants said they did know what their insurance covered, while three said they did not know. Maria says she knows because “most of the time, when you get there, they’ll tell you what’s covered and what’s not covered.” Susan described what her insurance covers: “What’s covered is your cleaning, X-rays, and probably extractions, but if you have to get something else done you might have to pay.” Adrienne also was able to elucidate what her insurance covers: “I don’t have everything on that. I have like cleaning and extractions…something like that…but just real expensive work, I don’t have that on there.”

There were a couple of reasons that participants said they did not know what their insurance covered. For one participant, she delegated the insurance work to her daughter: “If I don’t know, my daughter takes care of all that, and she finds out what’s what, and I don’t never need to worry about anything as far as medical or whatever. She oversee[s] it all, she acts more like a mother.” For another participant, it was because her insurance was just altered: “Well, they just changed my insurance without them telling me. I didn’t know they was going to change it, because of the situation that I’m going to be doing something different now, so they didn’t even send me no book or stuff in the mail, so I’ve probably just got to get on the phone and call them.” For the third participant, Medicaid did not inform her of what was covered: “I had to make all the calls because they didn’t tell me about the straight Medicaid, but I did get on the phone and I got the card right away.”

**Major theme 2. Attitudes**

Within the major theme of attitudes, there were three subcategories: attitudes towards oral health, attitudes towards dentists, and dentists’ attitudes towards their patients.

**towards oral health.** Overall, all of the participants across all four focus groups agreed that oral health was just as important and just as serious as other health problems. Many participants correlated oral health with other general health. Adrianne said, “I take it very seriously because if you don’t get your teeth and stuff taken care of – I know a lot of people who passed away by having bad teeth in their mouth, and they passed away behind that, by not getting proper dentists or not going back and forth to the dentist.” Tracy agreed, saying she learned the relationship on television: “On TV there was an ad with in going to the doctor the first thing the guy was told to do was first get his dental health taken care of because that effects a lot within his body.” Adrienne agreed with the link between oral and physical health: “I think because it will stir up other things in your body; where your teeth is bad other things will go bad in your body…your heart or with different things will go bad.” Cameron connected oral health to both mental and physical health:

They kind of go hand-in-hand because when you’ve got diabetes it affects your oral hygiene. When you have high blood pressure it also affects your oral hygiene. So, one effects the other. If you have high blood pressure, it can affect you, if you have bad teeth it can affect you… I think it also messes with your mental…your oral hygiene can mess with your mental as well because sometimes you can have so much pain until mentally it can give you a headache or it could cause you to have a problem.

For Maria, she just recently realized the link between oral and physical health:

I never did think that oral health problems were that serious until recently when I’ve heard…just as you were saying earlier, but I heard before…that having an infection or whatever in your mouth can cause other problems with other organs and even I heard not long ago that a child had passed because of his bad dental care. That made me think more about it, but before I didn’t think very much about it.

**towards dentists.** Within this subcategory, there were four primary attitudes towards dentists: problematic, nostalgia, pleasant, and fear. The dispersion of these results can be found in Table 2.

Table 2

*Attitudes towards Dentists.*

| Attitudes | Number of Participants | Percentage of Participants |
| --- | --- | --- |
| Pleasant/Good | 8 | 50% |
| Fear | 4 | 25% |
| Nostalgia | 3 | 19% |
| Problematic | 1 | 6% |

Eight participants said that they have a pleasant or good attitude towards their dentists. Four specifically said they feel comfortable with their dentist (all of which were in the second focus group), and two from the third focus group said they trust their doctor. One respondent in the second focus group said her attitude was happy:

I’m always happy – I don’t know, but I’ve seen people in there saying things, they look like – I guess it’s how they take pain, but they was coming out all like this. Some was coming out with pain medicine. I wasn’t even in pain. The lady at the desk was saying, “You still happy, and you got a smile on your face.” I said, that’s because it’s a good dentist.

Sydney also said she had a positive attitude towards her dentist:

I am fortunate because I’ve had very good experiences. I’ve had very good dentists. One dentist I went to for 20 some years. He passed. All of them have been really good with sitting down and explaining things. I find that…I would say that my dentist, except for the dentist I had, my dentists fire me because I tend to be so uptight when I go to the dentist. I finally found a dentist who said I can’t treat you with anything unless I put you to sleep. That’s helped me a great deal.

Within the subcategory of fear towards dentists, there were mixed responses. Four of the respondents said that they were fearful of dentist. Maria explained,

Well, it’s just the fear. It’s the fear. Now I have been to the dentist. I’ve had a tooth extracted over the years….a couple of teeth, as a matter of fact. I had a filling. The last time that I went it was devastating to me. I have a fear of the dentist. That last time I went it was it for me. I haven’t been back… I have a terrible experience when I go to dentists. I understand now that they’re putting you to sleep.

Karen agreed, “I’m having a problem with a fear that I can’t get off the curbs and get on escalators or anything now, so they’re sending me over to Providence. So I saw this doctor, he’s a psychiatrist, that’s what they said. And I went over there and the first time I went he gave me a pill, an anxiety pill, to combat this fear that I have.” Maria 2 explained that even though she is fearful, she still goes to the dentists: “but when I got to go, I don’t like the pain, I go.” However, ten participants expressly stated that they are not scared and have no emotion of fear towards their dentist. So while fear was an expressed attitude by some of the participants, a majority did not feel that way. As one respondent explained,

The good thing about it I have a dentist now…when you were younger you were afraid to go to the dentist because they would stick you before they pulled your tooth, and that hurt. Now I have a dentist who is pain free. Everything is absolutely pain free. He can pull your tooth and you don’t even know he’s done it. The man’s a genius.

Three participants expressed nostalgia towards the dentists they had in their youth, stating that dentists today are not like they used to be. Maria explained:

I remember when I was younger when I went to…there were some schools that had the dental clinics. I happened to go to Merit, which had the dental clinic. The kids were brought from around the area to go to that clinic to have their teeth cleaned and to check your teeth and have dental education. All of that was going on back in the ‘50s and ’60s. I don’t know when they stopped that, but they did have that back then which was good because it taught you as a child the importance of taking care of your teeth. I did for years till I got older. To have that in the school was really good because a lot of children took care of their teeth because of that, because they knew the value of that.

Liz agreed saying,

Back in the day the dentist took very good care of your mouth. There was a continuity. They know you. Now all of that is kind of vanished and changed. This question of liability and being under a group and all of those kinds of things have come into play… that many of us have had the best possible worlds as far as dentistry is concerned because my measurement of today’s dentists that I see is old Dr. Johnson who treated me when I was a child…9, 10, 11, 12 years old. His procedures and his approaches stand the test of time. I feel very fortunate to have had that experience because I find that today’s experience leaves a lot to be desired.

Only one participant explicitly stated that she had bad feelings towards her dentist. For her, it was not about pain, but about a lack of communication. Sarah said:

The problem I had with dentists …they’re not explaining what they’re supposed to be as far as I am concerned. Like she said, the dental bills are so high. If you’re going to pay all this money I want you to tell me this, this, this, and this. I am just not finding that I am getting there. I went to a dentist not too long ago and one dentist said you need this many teeth taken out, and then I go to another one and he said, well, you don’t need. I don’t know who to believe. I mean, do I need these four taken out or this dentist says you don’t need them. I don’t know what to do. I don’t know whether to have them removed or since he said, no. So, I am confused… they’re just not explaining. I mean, I had two teeth, he just pulled them. He didn’t say rinse, do blah, blah, blah, and charged me a lot of money. Even though I ask questions, it’s like they’re doing this and they’re rushing to get to the next person

**towards patients.** Within the subcategory of dentists’ attitudes towards their patients, the participants overwhelmingly expressed that their dentists treated them well. Five respondents said they were happy with how their dentist treats them, while another cited that her dentist treats her with respect. Two other participants said their dentist was gentle; one other pointed to kindness, and another pointed to friendliness. Adrienne said of her dentist: “Oh, nice. He has a good bedside manner… He’s gentle. He talks to me the whole time. He lets me know.” Another respondent pointed to strong communication, as well as citing gentleness, saying her dentist is “very gentle and understanding. He tells you everything.” Adrianne concurred: “My dentist treats me very nice…and he’s very, very gentle and kind.” Sarah explained, “Yeah, that’s nice, and she is very sweet, very kind, very nice doctor. She made me feel comfortable.” Karen said, “Mine, he almost acts like we’re friends.”

**Major theme 3. Behaviors**

There were two main subcategories within the major theme of behaviors – oral health routines and oral health pain.

**oral health routines.** Within the subcategory of oral health routine, participants discussed their daily routines, as well as how often they went to the dentist. Eight participants specifically pointed to daily brushing as their routine; three mentioned flossing; two cited brushing their tongue, and three cited the use of mouthwash. Moreover, five of the eight participants that discussed their daily routine cited multiple ways of keeping up their oral hygiene. As Cameron explained, “In my household its using a tongue brush to brush your tongue, brush your teeth, and if you wear dentures, to take your dentures out and not sleep in them and soak them and use everything you need to use to keep your mouth clean.” Sarah 3 concurred, saying, “We brush our teeth every day and we brush down on your gums and everything like that and floss your teeth and everything because your mouth carries so much bacteria from the food and stuff.” Liz pointed to “flossing after every meal, and then brushing.” Tracy, Karen and Alex cited the combination of brushing, flossing, and mouthwash. Tracy said, “The first thing I use when I go to the bathroom is use the mouthwash. Sometime I use the dental floss or whatnot, and then I brush my teeth.” Alex concurred, saying, “I floss in the morning, brush teeth, and use mouthwash. I also brush my tongue with the toothbrush.” There was an outlier, Carlos, who explained that his oral health routine was more a generalized health routine; he explained, “One of the most important parts of oral health in my household is washing your hands because your hands carry germs and bacteria. You don’t know if you serve somebody food all the bacteria can make the other person sick or if you work in a fast-food restaurant, your hands play a big part.”

The second part of oral health routines for participants was visiting the dentist. Eighteen participants said that they had a regular checkup from the dentist, while ten participants said that they did not receive regular checkups. Of those participants who said that they received regular check-ups, twelve said you should visit the dentist every six months, or twice a year, three participants said you should go every three months, and two participants said regular checkups are only needed once a year. A few participants explained why they did not receive regular checkups. Maria 2 said that “she’s too busy worrying about other people’s issues and not her own, so that’s why I haven’t.” Tracy explained that it gets harder to have checkups when you get older and do not have insurance:

I can tell you when I was working I used to go all the time…at least once a year. I had dental insurance or whatever. As you get older and you’re a senior and you’re not working anymore, it’s hard to get into a dental program where you can go at least once a year, six months, or whatever.

Adrienne said she is too busy with other things to go to the dentist regularly: “I use[d] to go every six months for a year, but I haven’t been in a while because I have a lot of other ailments and doctor appointments, so I just have made no appointments to go.”

However, a number of participants expressed how important they thought it was to go to the dentist and have a regular checkup. Sydney said, “I generally go to the dentist. I had gingivitis so I tend to go every certain amount of months. I always go to the teeth cleaning. I think dental work is very important… The dental cleaning twice a year.” Sarah 2 concurred, citing past personal experience as the reason she goes regularly: “I go to visit my dentist every six months…I know it’s very important to visit the doctor because I lost my root canal in my teeth from when I had the pain for almost 18 years.” A respondent from the second focus group also pointed to personal experience as necessitating regular checkups: “I go every three months for cleaning and examination – and when I went to see them last month, it seemed like when I would eat or drink something cold, it would kind of ache, so what they did was I had to go back and he had something that he put on the tooth, and that took care of it, so I don’t have that problem anymore.”

**oral health pain.** The second subcategory from the major theme of behaviors is oral health pain. This subcategory included missing work because of mouth pain, visiting the emergency room because of mouth pain, and self-treatment for mouth pain. Nine participants said that they had, at least once, missed work because of mouth pain, while seven others said they never had. Given the response rate of this question, the results were fairly evenly distributed between having missed and never having missed work because of mouth pain. Those who had missed work explained why. As Maria explicated:

I did years ago. Years ago when I had my first tooth extracted. The pain was woooo. I’ve had five children and that tooth pain was worse than having my babies. I am serious. It is terrible. I am not exaggerating. I did miss work because I had to go get that tooth taken care of.

Karen also shared her experience with missing work because of oral pain:

I did when I fell and knocked everything out, and I missed about a week’s work. I was on my way to work when I fell. I was running to catch the bus to go to work, and I tripped over a tree branch and that’s when I went face down. And I was out of work for about two weeks because the ambulance took me straight to Howard. That’s where they gave me the 22 stitches up there that night.

Sarah 2 said she missed work after pulling out one of her own molars: “When I pull[ed] out the molar, I used to continue bleeding all the time. Sometimes the next day, too. So I can’t go to work like that with the bleeding, even I put in the gauze every time.” Shawn also described an oral infection that kept him from work:

I had that one time when I was home. I was in such pain. This dentist was so…I called him and I was crying. What he did was he actually called in a prescription for me to ease the pain. I went and picked up this prescription. All this green stuff came out. It cleared it up. It cleared up between that time and the next time I had to go see him for my next appointment.

Within the subcategory of oral pain, three participants said they had ever gone to the emergency room because of pain, while ten explicitly stated that they had never been. Sarah explained her reason for going to the emergency room: “I did once. My jaw was hurting. It was hurting so bad I went and the doctor said it was a tooth. It was like at one o’clock at night.”

Finally, within the theme of behaviors, participants discussed their methods for self-medicating, rather than going to the doctor. Fourteen participants said they had self-medicated for oral pain before. The distribution of self-treatment methods can be found in Table 3 below.

Table 3.

*Self-Medication of Oral Pain*

| Type of Self-Medication | Number of Responses | Percentage of Participants |
| --- | --- | --- |
| Garlic | 2 | 15% |
| Acetaminophen | 2 | 15% |
| Orajel | 2 | 15% |
| Salt Water | 2 | 15% |
| Ambesol | 1 | 15% |
| Lemon | 1 | 7% |
| Tea bag | 1 | 7% |
| Peroxide | 1 | 7% |
| White Vinegar | 1 | 7% |

Tiffany specifically said she used self-medication so that she would not have to go to the emergency room: “I wanted to say I stopped it on my own so I didn’t have to go to the emergency room, because I had fresh piece of garlic, and I just stuck it up on the side of my gum and the it knocked out the pain right away.” Maria 2 said she has used lemon extract to help with pain in teeth, while Adrianne cited a tea bag to take away the pain. Others cited over-the-counter medications like Motrin, Bayer, Orajel and Ambesol. Participants said they self-medicated when they either could not get an appointment with their dentist or did not want to go to the emergency room.

**Major theme 4. Barriers**

Within the theme of barriers, there were three different categories that emerged: access, cost, fear/pain.

**access.** There were three subcategories that developed from the category of access: access to dentists, work policies and access, and transportation and access. Overall, participants said that it was easy to find dentists in their neighborhood. Nine participants said it was easy, two said it was somewhat easy, and five said it was difficult. For those who said it was easy, they could locate a dentist through a variety of methods. For Roxanne, she got hers “off of somebody’s Facebook page.” Another respondent goes “through 1-800-Dentist,” while another said she had a list. Liz said her insurance company sent her a list as well:

They send you a form in your mailbox. It’s a booklet that Medicaid gives you, you look up and you can find your own dentist. It’s like a pamphlet, but it’s like a book. And you could find dentists. Whatever you want, they give you options…I mean I haven’t had that problem, but I get it every year at my mail slot because I have Medicare and Medicaid.

Maria also thought it was fairly easy to find a dentist but conceded the issue of insurance could affect the ease with which someone could get a dentist:

Well, they do still have quite a few dentists out here that are like family dentists or whatever that people frequent a couple of times a year on a regular basis. They do have them out there. I mean, the question about insurance and everything is true, but it still stands the fact that they are available, they’re there. It’s not that they’re not there because they have family practices all over that you could go to. The issue does come up about the insurance and the cost and everything. I don’t think the problem is not having anywhere to go because there are…they’re out there, it’s just the problem of insurance or if you can afford it.

Pearl, who thought that it was difficult to find a doctor because she had trouble “knowing where to go. They keep changing” would like to have a list of dentists who are close by. Other participants highlighted the reasons they thought finding a dentist was hard. Liz explained:

I don’t think it’s easy at all. Many times you rely on word-of-mouth, a referral by someone else who has had dental work. In our community here we have any number of private dental operations spotted around in the community. We have one clinic that I know of. Further north, Howard University, has a dental clinic for older people that does provide services. By and large it is not easy.

For Cameron, it is an issue of insurance:

Some dentists don’t even take Medicaid. I find that kind of annoying because right now I am getting ready to switch over where I have Medicare and Kaiser. What purposes does it serve when you can’t even use it for the things you need to use it for?

This issue of insurance was echoed by Liz who noted, “Now that we’re drawing it out, it begins to look like the dental folk didn’t keep up with the times in so much as with this insurance business, which is very complicated.”

The second subcategory within this theme of barriers is work policy and access. Overall, work policies on leave for dental appointments was not a barrier for participants. Ten participants said they had a flexible work policy that allowed them to take leave to go to the dentist; only two participants said there was no such policy and that they had difficulty getting to dental appointments. Sarah explained her work’s policy in the government:

When I was in the government you took your leave and you went. Just happened the dentist I was going to, if my appointment was at eight, he took me at eight…I worked for the government and I had to take my leave. You got this set policy where you can go…not for the government.

Maria agreed, saying, “When I was working in the government that is what you had to do. You had to use your sick leave or annual leave if you wanted to make an appointment…go to an appointment for a doctor…any kind of doctor…dentist or whatever.” Cameron’s work also had a flexible leave plan:

Most of the time now you could use your Maxiflex…they give you options. You can take Maxiflex and use that time to go to the dentist or you could come back…not lunch…come back to work. I am on a no lunch schedule. When I’ve got to do something I go do it and just work an extra hour when you come back. They give you that option there that they didn’t give us before.

Another respondent explained her not-for-profit institution’s policy, “They don’t pay overtime. If you have to go to the dentist just say you have a dental appointment.”

The third subcategory within access is transportation. Once again, participants did not see transportation as a barrier. Twelve participants said that it was not a problem and was easy to get to their dentist appointments, while only one participant said it was hard. Five out of those twelve participants noted that it was so easy because their insurance paid for their transportation. As one respondent said, “Um hmm. Medicaid pays for this. I’m not scared yet from riding buses and subways. I’ll get on them and go where I want to go.” Another respondent agreed, noting, “They got Medicaid now, and Medicaid pay for all that, too.” Yet another respondent concurred, saying, “Yes, it’s free. Medicaid transportation is free, because my sister has Medicaid and her transportation is free. Just got to call…with most of the insurance you have to call at least seventy-two hours for your transportation.” Two other respondents did not specifically cite Medicaid, but they did say that their insurance pays for transportation. Angie said, “most health insurance provides transportation,” and another participant added, “They have transportation for the insurance card, too. You just have to call and find out.” In addition to those five, one other said she would ride the Metro or a bus, while two others said they would even take a taxi. Said one respondent, “And I’ll ride that $5 taxi, too.” The second respondent agreed, saying, “Amen, I do too.”

There was one outlier, who said she had difficulty getting to her appointment. As she explained, “For me it’s a problem in transportation to get from where I live to way up Northwest, almost to Silver Spring.”

In terms of how long it took participants to get to their dentist, there was also not much of a barrier. Five participants said it took them twenty to thirty minutes to get to their dentist, two said it was not far, and another two respondents said it was easy to get there. Only one participant said it was far for her to travel to the dentist, but she noted that it wasn’t a barrier: “I come from Maryland to D.C. to go to my dentist, and that’s a good ten miles. But like I said, because he’s so good and pain free, I don’t mind doing that, not at all.”

**cost.** The second category within the theme of barriers was cost. Unlike the previous category, many participants feel as if cost is a barrier to receiving dental care. The results are demonstrated in Table 4 below.

Table 4

*Cost as Barrier to Dental Visits*

| Themes | Number of Participants | Percentage of Participants |
| --- | --- | --- |
| Cost as a Barrier | 11 | 91% |
| Insurance Covers Cost/Cost is Not a Barrier | 1 | 9% |

Eleven participants said that they felt cost was a barrier, while only three participants said their insurance covered enough of their dental care to make cost not a problem. Participants who saw cost as a barrier, had very specific stories and challenges that related to the cost of dental care. Sarah explained:

[I] spent $50,000 on [my] mouth and didn’t have dental insurance. I paid it when I was working. I am older now and I have two grandkids that didn’t have a father. I tend to want them to have beautiful teeth so I sort of neglected myself…even though I have dental insurance. It was just that it is very expensive and I felt these kids being young. Before I used to go at least three or four times because I had gum disease. I put that off because of other things. I was running back to Detroit to see my mother, which I should have been taken care of. That was my problem. It wasn’t really the insurance because I do have the insurance, but I was paying for the kids out of pocket because they didn’t have the money.

Another respondent concurred, saying, “the insurance is not like it used to be. Trying to pay for the insurance. The dental is so high now. You have a lot of problems trying to find a dentist that’s reasonable that you can go do.” Liz said, “As the individual, the economics play such a big important part in whether you will or whether you won’t or whether you can or whether you can’t.” Adrienne said that her other appointments had been taking higher priority to your dental appointments, particularly, “these doctor bills. You name it, I have it.” Ellen also had a horror story as it related to insurance and cost:

I was on Bravo, too, and they waited until I was getting ready to have surgery and the people called me and told me I couldn’t have the surgery because Bravo wasn’t going to pay for it. So I’ve switched now to Cigna. But isn’t that something?

Sarah also had problems with insurance and payment:

Yesterday I had another surprise. I called to the New Hampshire Pharmacy for the supplies, because her primary doctor sent the paperwork to get the supplies. And when I called yesterday to see if they received the paperwork by fax, they said they received the paperwork but the problem is Bravo, they don’t pay for any supplies. Nothing. I can’t do nothing, because she has the lawyer, and she doesn’t get the care.

Karen said that she had had trouble with payment and insurance until she switched:

One time, two years ago, I was paying so much money, co-payments for going to the doctor, medicine. Medicine was costing anywhere from $30 to $50 for a prescription. And what my daughter did, she called down to the Medicare legal department and made an appointment for us to speak to a lawyer, and we went there and she and I explained to the man that was assigned to us the problem that I was having, that I was on a fixed income, and the medicine and the medicine and co-pays and things was so high. And he in turn connected me with a different insurance. I had Bravo, so he cut me off and I said take me off of that, and he put me on a different insurance, and after that when everything was squared away, I go to the doctor now and unless it’s a specialist, I pay no co-pay. I go to the drugstore to get my prescription and pay $6 or something like that, from $35 or $45.

Overall, cost was often prohibitive for dental care. As Sarah 2 said:

Maybe that’s why a lot of people don’t go to the dentist. They can’t afford it and it’s so expensive. For instance, you have a heart problem or some other problem that you see more pressing. You forget the down part and take care and say, well, this is going to save my life. I hear a lot of people saying the same thing…I put off the dentist because it is very expensive.

Liz pointed to the trouble of cost that happens in the community: “I remember reading something in the paper a while back…a young boy in D.C. The mother could not afford healthcare and he died. So, in some way the community needs to make healthcare more affordable.

Three participants were the outliers who said that their insurance covered dental care so that cost was not a barrier. Tracy explained, “I’ve been fortunate in having a dental plan through my employer so I spend very little money out of my pocket.” Alex concurred, saying:

I’ve got insurance and it covers a hundred percent of preventive maintenance for two times a year, et cetera. Anything that requires orthodontia I have healthcare that comes out of after tax money. You basically have to know…you have to have a separate savings account for that. I am sorry, it’s pretaxed.

Despite participants’ difficulties with cost, they overwhelmingly responded that they have had no troubles or problems having their insurance processed by the dental clinic or with their dentist accepting Medicaid. Fourteen respondents said that they have never had a problem, while only one respondent said that he had had some initially trouble with Medicaid, which had ultimately been resolved.

**fear/pain.** The final category of the major theme of barriers, is fear and pain. There were mixed responses to this category, with seven participants saying that fear and pain are barriers that prevent them from going to the dentist and four participants saying that fear and pain do not stop them from going to the dentist. These split responses indicate that fear and pain are a relatively subjective gauge to set as a universal barrier. Sarah explained why some people don’t go to the dentist:

Maybe that’s why a lot of people don’t go. The experience of the dentist…I know I need to go, but. I know those needles and I know this is going to hurt, so they keep putting it off and putting it off. Maybe that’s why a lot of people don’t.

Another respondent echoed this sentiment, saying, “ I’m scared of needles.” Yet another participant concurred, saying “a lot of people be scared. They don’t want to go back.” For a different participant, it was the pain of teeth cleaning:

But the man that was cleaning my teeth, I didn’t really like how he was cleaning my teeth. It hurt. I had to fake him out like I was really having more pains to make him hurry up and finish my mouth.

Of the four participants who said fear and pain were not a barrier to going to the dentist, two acknowledged that they did not like going, but went anyways. As one respondent said, “I don’t like it, but I go.” Another concurred, saying, “It’s just like anything else. You’re tired of going to the doctor, but you got to go.” There was, however, one significant outlier who said she not only did not fear going to the dentist but enjoyed it: “I love going to the doctor and the dentist.”

**Major theme 5. Needs**

The fifth major theme is needs. Within this theme, there are two main categories – dental qualifications and health communication.

**dental qualifications.** There are five subcategories within the category of dental qualifications: communication, bedside manner, trust, cleanliness, and experience. The distribution of these needs are listed below in Table 5.

Table 5.

*Needs for Dental Qualifications*

| Themes | Number of Participants | Percentage of Participants |
| --- | --- | --- |
| Communication | 9 | 37.5% |
| Trust | 6 | 25% |
| Bedside Manner | 3 | 12.5% |
| Cleanliness | 3 | 12.5% |
| Experience | 3 | 12.5% |

Overall, participants were satisfied with their dentists and felt as though their needs were being met. Thirteen participants said their needs were being met and four participants said that their needs were not being met. Liz said she has been satisfied with the same dentist for “20 years,” while Karen said her needs are met “100%”

Communication was the biggest need that participants cited when it comes to dental qualifications, with nine participants noting its importance. The type of communication participants wanted somewhat varied, ranging from in-depth explanations, more one-on-one time, to consistent opinions. Five out of the nine participants (55%) who cited communication as a need specifically pointed to the need for in-depth explanations. As Will noted:

Why is a priority different from dental work than it is for any other operation you might have? It’s always a preparatory type thing. Like, when I had a hernia, they prepped me. They told me what I had to do prior to, so I needed to get a consult. I needed to sit down with the doctor before the operation so I understand exactly what my limitations, do I agree with this, do I not, and then when the operation is over I am satisfied. But to go in blind and not with a consult, I don’t like that. I like to sit down and talk to the doctor prior to…or the technician.

Sydney agreed, saying, “I like ones that will explain what they’re doing in detail.” Sarah echoed those sentiments, noting, “The dentists don’t seem to have a lot of time to explain things to you anymore. If you’ve got a tooth that’s got to be pulled, he’s going to pull the tooth but he doesn’t say something else.” Maria emphasized the need to self-advocate for explanations from dentists:

If you insist on [it]…I say that about any doctor, dentist, whatever. A lot of people just will not ask questions and will not insist on answers. I am not going to let them to do anything to me unless they full answer all of my questions. You’ve got to tell me why and what benefit is it going to be as opposed to this or that. Don’t let anybody just do anything. If you’ve got a question that you really need to be answered, then they need to answer it. If they can’t answer it then you don’t need to go with them.

Sarah 2 also highlighted the importance of self-advocating for dentist’s explanations:

I tend to write things down throughout the year what’s going wrong with me from the last time I went. I went to my doctor and I had these four things that I wanted to ask him, which would take one minute. The next time I went he said how many do you have this time? I am very easy going. I said, wait a minute, you’re supposed to be my doctor. I need these four questions answered and if you don’t answer them I’ll find another doctor. She’s right. You have to take things into your own hands. You can’t just let the doctors get away with things.

Within these responses about in-depth explanation, respondents also alluded to another need they have – for one-on-one discussions with their doctor. One respondent said bluntly, “I am not getting one-on-one…I am not getting that contact.” For respondents like Marie and Sarah, one-on-one is a necessary pre-condition of in-depth explanations, so the two needs go hand-in-hand.

For three other participants, they wanted consistent communication between dentists. As with one-on-one communication, this subcategory linked to the need for in-depth explanations from dentists. As one respondent noted:

My dentist couldn’t take me so he referred me to another dentist. This is the one that says have four and the other one I asked, he says, well, I am going to send you over here to have these two. I said, well, do I need these four and he says, no. They weren’t my primary dentist. Now I’ve got two dentists saying two different things. Of course, I didn’t know what to do. He pulled the two, but I didn’t let him take the other four out because I didn’t know what to do. And they didn’t give any explanation. You know you want to ask questions and they want to get you out of here, you’re not my patient. He sent me over here…I pull these and you’re out of here.

Another respondent agreed, saying:

If you’re going to pay all this money I want you to tell me this, this, this, and this. I am just not finding that I am getting there. I went to a dentist not too long ago and one dentist said you need this many teeth taken out, and then I go to another one and he said, well, you don’t need. I don’t know who to believe. I mean, do I need these four taken out or this dentist says you don’t need them. I don’t know what to do. I don’t know whether to have them removed or since he said, no. So, I am confused.

The next subcategory was bedside manner. Three respondents said that they need a dentist who makes them feel comfortable and is gentle. Liz said that she needs a dentist who is “very patient and calm and calming.” Another respondent said she wants a dentist who is “very, very gentle and kind and he talks…he makes me comfortable. Adrienne agreed, noting that all she really needs is “a nice doctor.”

The third subcategory of dental qualifications is trust. As previously mentioned within a different theme, most participants already trust their dentist. However, these participants also emphasized the need for trust between a dentist and patient, with six participants expressly citing the need for trust. One respondent simply noted, “Trust is very important.” Sydney agreed, saying, “It is. I’ve had several instances where I have not trusted the dentist and got into a lot of serious trouble.” Karen noted that she has had the same dentist for 20 years and that she has that trust that she needs, while another respondent said the same thing of her dentist of 30 years.

The fourth subcategory within dental qualifications is the need for cleanliness. Three participants mentioned that cleanliness was an important need to fulfill. Adrianne said that she requires a dentist’s office to be “neat and clean, well taken care of, and they wash their hands and stuff like that. And brand new equipment, and not wash it and use the same thing in my mouth and in her mouth.” Another respondent agreed, saying she notices when and if “they pull it off the little plastic thing, and then they lay it on a little tray. And then they cover it up.”

The fifth and final subcategory for dental qualifications is experience. Three respondents noted the need for experience. As one respondent noted:

Yes, when I go to see the dentist, I like see how many years has experience. Make sure, because experience is very important. Because sometimes they open the clinic, you don’t know how many years has the experience, maybe it’s just graduate. I don’t have problem with doctors who just graduate, but it’s very important in somebody you could trust who has more experience. Reference is very important, too. The reference and the experience of other people.

Angie agreed, noting, “Look, I got to ask them, what kind of dental master degree. I want to know, before you even work on my mouth, because I don’t want you to be one of them students that you don’t know what you’re doing… Are you qualified to work in my mouth?

Another respondent agreed, saying, “yeah, sometimes they send medical students who don’t know what they’re doing.

**oral health communication.** The second category that emerged from the theme of needs was oral health communication. Within this category, respondents explained what kind of communication mediums and through which media that needed to receive oral health communications. Nine participants cited mail as their preferred method; four participants cited every method of communication and four said they preferred to get oral health communication from their dentist or doctor; three participants cited television and three others cited the internet; two participants cited visual presentations, two others cited brochures, two more said forums and discussions like these focus groups, and one said that they preferred mixed media efforts.

The two participants who cited visual presentations had different ideas for how information should be presented. Liz said, “I wish that there was some way that you could see actual…a visual presentation of the connection between bad oral care and how it affects you physically. See it.” She also suggested a film: “Howard University produced a fantastic film for children on dental health. It is fantastic. It was cosponsored by the Linx Organization. That might be something you might want to look into. That would be just wonderful even for adults to look at.” Another respondent said, “What about also having a dentist come in who could even have slides or something to show exactly what this oral stuff is…the cancer and what happens.”

Participants also explained why they believed the mail was the best way to receive oral health communication. One respondent noted: “I don’t give out a lot of my email, even though I have a computer because I am not one of those that’s going to sit at the computer every day…To keep receiving stuff online causes you to be at the computer so long. That’s not a thing I do.” Instead she said, she prefers the mail, “Then I can read it when I feel like it.” Pearl explained that she liked to have something tangible both to read and then also to give to other people, in order to advance knowledge about oral health: “Well, if they could send something to [me], they can send something to us; we can pass it on too.”

For another respondent, all different mediums were important to get the oral health message out:

I think that a mixture of media is helpful. People get tired of it. They will take a brochure and it comes in the mail and after a while they stop reading it. The same thing happens with magazines. If you can get a variety so that people can change around and exchange and share with somebody then I think you’ll read a larger number.

**Summary**

There were three categories within the major theme of knowledge: oral hygiene and health, prevention and oral health, and oral care and insurance. Within the category of oral hygiene and health, there were four subcategories: knowledge about hygiene, about oral health and other diseases, oral health and ageing, and risky behaviors for oral health. Within the subcategory of hygiene, nine participants characterized their knowledge as taking care of their mouth, including brushing, flossing, and gums; two participants described their oral hygiene knowledge as regular visits to the dentists, and one said its taking care of the total body. Within the subcategory of oral health and disease, there was primarily a lack of knowledge. Twelve participants noted that they had never talked with their dentist about diseases that could connect to oral health, such as HPV, cardiovascular disease, and diabetes; only seven participants said their dentist had ever even mentioned these diseases. Within the subcategory of oral health and ageing, most of participants did seem to have accurate knowledge. Ten of the participants said that losing teeth was *not* a normal part of getting older; two said it could be; and only three said that yes, losing teeth was just a part of getting older. The last category in this major theme of knowledge was risky behaviors for oral health. Within this category were three subthemes – food, beverages, and bad habits.

Within the second category of prevention and oral health in the first theme, there were two subcategories that emerged – communication about oral health and prevention and knowledge about food and beverages that helped with oral preventive care. Ten participants noted that they had received information about oral health and preventative care that was connected to other issues like oral care hygiene, smoking cessation, or dietary counseling, while only three participants said that they had never received such information. In addition to knowledge about oral health and prevention that had been communicated, participants were able to discuss their knowledge about food and beverages that were actually helpful in oral health care. Eight participants noted that water was essential for strong oral health and five participants said milk, with its calcium, was important to oral health. Three participants pointed to vegetables and two others to fruits.

The final category in the theme of knowledge is awareness of and about oral care and insurance. All but one participant said that they had the knowledge of a place they could go to if they had a dental emergency other than the dentist’s office*.* Twelve participants noted that they could go to the hospital, including the emergency room, or a clinic. The final aspect of knowledge was if participants knew what was covered by their dental insurance. Eight participants said they did know what their insurance covered, while three said they did not know.

Within the major theme of attitudes, there were three subcategories: attitudes towards oral health, attitudes towards dentists, and dentists’ attitudes towards their patients. Overall, all of the participants across all four focus groups agreed that oral health was just as important and just as serious as other health problems. Many participants correlated oral health with other general health. Within this subcategory of attitudes of dentists, there were four primary attitudes: problematic, nostalgia, pleasant, and fear. Eight participants said that they have a pleasant or good attitude towards their dentists; four of the respondents said that they were fearful of dentist; three participants expressed nostalgia towards the dentists they had in their youth, stating that dentists today are not like they used to be; and only one participant explicitly stated that she had bad feelings towards her dentist. Within the subcategory of dentists’ attitudes towards their patients, the participants overwhelmingly expressed that their dentists treated them well. Five respondents said they were happy with how their dentist treats them, while another cited that her dentist treats her with respect. Two other participants said their dentist was gentle; one other pointed to kindness, and another pointed to friendliness.

Within the third major theme, behaviors, there were two main subcategories within the major theme of behaviors – oral health routines and oral health pain. Within the subcategory of oral health routine, participants discussed their daily routines, as well as how often they went to the dentist. Eight participants specifically pointed to daily brushing as their routine; three mentioned flossing; two cited brushing their tongue, and three cited the use of mouthwash. The second part of oral health routines for participants was visiting the dentist. Eighteen participants said that they had a regular checkup from the dentist, while ten participants said that they did not receive regular checkups. The second subcategory from the major theme of behaviors is oral health pain. This subcategory included missing work because of mouth pain, visiting the emergency room because of mouth pain, and self-treatment for mouth pain. Nine participants said that they had, at least once, missed work because of mouth pain, while seven others said they never had. Within the subcategory of oral pain, three participants said they had ever gone to the emergency room because of pain, while ten explicitly stated that they had never been. Finally, within the theme of behaviors, participants discussed their methods for self-medicating, rather than going to the doctor. Fourteen participants said they had self-medicated for oral pain before.

Within the fourth theme of barriers, there were three different categories that emerged: access, cost, fear/pain. There were three subcategories that developed from the category of access: access to dentists, work policies and access, and transportation and access. Overall, participants said that it was easy to find dentists in their neighborhood. Nine participants said it was easy, two said it was somewhat easy, and five said it was difficult. The second subcategory within this theme of barriers is work policy and access. Overall, work policies on leave for dental appointments was not a barrier for participants. Ten participants said they had a flexible work policy that allowed them to take leave to go to the dentist; only two participants said there was no such policy and that they had difficulty getting to dental appointments. The third subcategory within access is transportation. Once again, participants did not see transportation as a barrier. Twelve participants said that it was not a problem and was easy to get to their dentist appointments, while only one participant said it was hard. In terms of how long it took participants to get to their dentist, there was also not much of a barrier. Five participants said it took them twenty to thirty minutes to get to their dentist, two said it was not far, and another two respondents said it was easy to get there.

The second category within the theme of barriers was cost. Unlike the previous category, many participants feel as if cost is a barrier to receiving dental care. Eleven participants said that they felt cost was a barrier, while only three participants said their insurance covered enough of their dental care to make cost not a problem.

The final category of the major theme of barriers, was fear and pain. There were mixed responses to this category, with seven participants saying that fear and pain are barriers that prevent them from going to the dentist and four participants saying that fear and pain do not stop them from going to the dentist.

The fifth major theme is needs. Within this theme, there are two main categories – dental qualifications and health communication. There are five subcategories within the category of dental qualifications: communication, bedside manner, trust, cleanliness, and experience. Overall, participants were satisfied with their dentists and felt as though their needs were being met. Thirteen participants said their needs were being met and four participants said that their needs were not being met. Communication was the biggest need that participants cited when it comes to dental qualifications, with nine participants noting its importance; the next subcategory was bedside manner where three respondents said that they need a dentist who makes them feel comfortable and is gentle. The third subcategory of dental qualifications is trust. As previously mentioned within a different theme, most participants already trust their dentist. However, these participants also emphasized the need for trust between a dentist and patient, with six participants expressly citing the need for trust. The fourth subcategory within dental qualifications was the need for cleanliness. Three participants mentioned that cleanliness was an important need to fulfill. The fifth and final subcategory for dental qualifications was experience. Three respondents noted the need for experience.

The second and final category that emerged from the theme of needs was oral health communication. Within this category, respondents explained what kind of communication mediums and through which media that needed to receive oral health communications. Nine participants cited mail as their preferred method; four participants cited every method of communication and four said they preferred to get oral health communication from their dentist or doctor; three participants cited television and three others cited the internet; two participants cited visual presentations, two others cited brochures, two more said forums and discussions like these focus groups, and one said that they preferred mixed media efforts.
