# Supplementary material for: A qualitative study of oral health knowledge among African Americans
Source: PLoS One. 2019 Jul 10;14(7):e0219426. doi: 10.1371/journal.pone.0219426 (PMC6619789; doi:10.1371/journal.pone.0219426)
Supplement: S2 Text — This is the Church 1 focus group transcription. (DOCX) [file pone.0219426.s002.docx]

**GEORGETOWN-LOMBARDI HEALTH DISPARITIES INITIATIVE**

**Oral Health Focus Group Transcription– Church 1**

**March XX, 2016**

**Project #GGT0421-16**

**ICE BREAKER**

M: To start off I’d like for us to go around the table, say our pseudo name and one thing that you enjoy to do as a hobby. We’ll start with Sydney.

R: My name is Sydney. I love to crochet. It’s something that I am just learning and I have found I just really love it.

R: Will. Working with my hands.

R: I am Sarah. I love decorating.

R: I am Adrienne. I love to cook.

R: I am Maria. I love to play cards and eat.

R: I am Sasha. I like cards and board games.

R: I am Liz Taylor and I love to act. My favorite picture was where I was riding the horse. I can’t remember the name of the picture, but there was a black horse in the picture.

R: Black Beauty.

R: I am Tracy. I love to play games on the computer or the tablet.

R: I am Carlos. I love going to the casinos.

R: I am James. I love to work with my hands.

**ORAL HEALTH**

M: Thank you everyone. We’ll get started. Could you tell us what comes to mind when you hear the term oral health care?

R: Oral health care, to me, is taking care of your teeth, your mouth, your gums.

M: Thank you, Maria.

R: This is Maria. I’m sorry. Oral health care to me is taking care of your mouth, your teeth, your gums.

M: Thank you, Maria. Anyone else?

R: My name is Sydney. Oral health to me means brushing…taking care of your teeth, doing the things that you need to do, visiting the dentist, and fear and panic.

M: For many people. Thank you, Sydney.

R: I am Tracy. Oral health care to me is going to the dentist at least once a year to get your teeth checked, make sure you brush them every day.

M: Anyone else?

R: My name is Liz. Oral healthcare has changed over the years. Thirty, forty, years ago it would have been one thing, but now that I am an older person it means really being diligent about good hygiene, oral hygiene.

M: Thank you.

R: My name is Adrienne. Oral healthcare means to…well, to make sure you keep your teeth clean and like going to the dentist at least…but some people go six…within six months…because you do have infection under your gums. It means to make sure you stay with the dentist and keep it checked.

M: Thank you, Adrienne. Do you think that oral health problems are as serious as other healthcare problems, and please tell me when you answer why or why not.

R: This is Adrienne. I think because it will stir up other things in your body; where your teeth is bad other things will go bad in your body…your heart or with different things will go bad.

R: Would you ask the question again?

M: Sure. Do you think that oral health problems are a serious as other healthcare problems, and please tell us why or why not.

R: Well, this is Maria. I never did think that oral health problems were that serious until recently when I’ve heard…just as you were saying earlier, but I heard before…that having an infection or whatever in your mouth can cause other problems with other organs and even I heard not long ago that a child had passed because of his bad dental care. That made me think more about it, but before I didn’t think very much about it.

R: My name is Cameron and I think it’s…they kind of go hand-in-hand because when you’ve got diabetes it affects your oral hygiene. When you have high blood pressure it also affects your oral hygiene. So, one effects the other. If you have high blood pressure, it can affect you, if you have bad teeth it can affect you…they go hand-in-hand.

M: Any other thoughts on that?

R: I think oral health is very important. My name is Will. The pain that goes with when you have oral problems is tremendous for me. I am having a lot of things being done now…more or less preventive maintenance to have it checked periodically so you know exactly where you are…your status. It’s very painful to me. I don’t know about anybody else.

R: Some say it can be as bad as child birth.

R: Is that right? I wouldn’t know.

R: Cameron, again. I think it also messes with your mental…your oral hygiene can mess with your mental as well because sometimes you can have so much pain until mentally it can give you a headache or it could cause you to have a problem.

R: This is Liz. I wish that there was some way that you could see actual…a visual presentation of the connection between bad oral care and how it affects you physically. See it.

M: Thanks Liz, we are working towards that so we can show the connections and help to decreases disparities. What is the importance of oral care in your household?

R: Cameron. In my household is using a tongue brush to brush your tongue, brush your teeth, and if you wear dentures, to take your dentures out and not sleep in them and soak them and use everything you need to use to keep your mouth clean.

M: Anyone else?

R: Liz. Flossing after every meal, and then brushing.

R: This is Maria. For me in my household brushing, rinsing, all of that is important to me, which I do a couple of times a day. Going to the dentist is not.

M: Why not?

R: Well, it’s just the fear. It’s the fear. Now I have been to the dentist. I’ve had a tooth extracted over the years….a couple of teeth, as a matter of fact. I had a filling. The last time that I went it was devastating to me. I have a fear of the dentist. That last time I went it was it for me. I haven’t been back.

R: Tracy. Trying to get to a dentist. The insurance is not like it used to be. Trying to pay for the insurance. The dental is so high now. You have a lot of problems trying to find a dentist that’s reasonable that you can go do.

M: These are good concerns that are being raised. Anyone else?

R: Say the question again because I was thinking about something. I am Sarah.

M: Sarah, what is the importance of oral care in your household?

R: I suppose to have had a good dentist, but the problem I had with dentists…it’s not in the household…they’re not explaining what they’re supposed to be as far as I am concerned. Like she said, the dental bills are so high. If you’re going to pay all this money I want you to tell me this, this, this, and this. I am just not finding that I am getting there. I went to a dentist not too long ago and one dentist said you need this many teeth taken out, and then I go to another one and he said, well, you don’t need. I don’t know who to believe. I mean, do I need these four taken out or this dentist says you don’t need them. I don’t know what to do. I don’t know whether to have them removed or since he said, no. So, I am confused.

M: That’s a big decision to make.

R: That’s right. I am confused. I don’t know whether to take them out. I don’t want to take them out. Do I take them out because of health sake? They’re just not explaining. I mean, I had two teeth, he just pulled them. He didn’t say rinse, do blah, blah, blah, and charged me a lot of money. Even though I ask questions, it’s like they’re doing this and they’re rushing to get to the next person.

R: If they don’t bother you why take them out?

M: Well, it depends on what the reasons are for the dentist suggesting that they be pulled. That’s something should tell you. However, you can also get a second opinion from another dentist to be sure that it is needed. In many cases other treatments be done so you can save your teeth rather than having to pull them, but it depends on each individual situation.

R: That’s exactly what I wanted to hear. I am saying to myself, okay, if I’ve got a gum disease or something, can you tell me what to do without pulling out my teeth right now? No, these got to come out. The other dentists said no. So, I am in between. I don’t know what to do.

M: I understand.

R: This is Will. Why is a priority different from dental work than it is for any other operation you might have? It’s always a preparatory type thing. Like, when I had a hernia, they prepped me. They told me what I had to do prior to, so I needed to get a consult. I needed to sit down with the doctor before the operation so I understand exactly what my limitations, do I agree with this, do I not, and then when the operation is over I am satisfied. But to go in blind and not with a consult, I don’t like that. I like to sit down and talk to the doctor prior to…or the technician.

M: Will, do you feel that your dentist does not explain?

R: I am not getting one-on-one…I am not getting that contact.

R: I get it from the technician or the receptionist.

M: How often do you and your family have dental appointments and please tell me why.

R: Tracey. I can tell you when I was working I used to go all the time…at least once a year. I had dental insurance or whatever. As you get older and you’re a senior and you’re not working anymore, it’s hard to get into a dental program where you can go at least once a year, six months, or whatever. You have a toothache you have to get money together to go have your tooth pulled. It’s $200 or $100 or whatever. When you’re working and you’ve got insurance, you just go. It’s just harder.

M: I see that Cameron and Sydney agree with you.

R: I am Sarah. Sarah spent $50,000 on her mouth and didn’t have dental insurance. I paid it when I was working. I am older now and I have two grandkids that didn’t have a father. I tend to want them to have beautiful teeth so I sort of neglected myself…even though I have dental insurance. It was just that it is very expensive and I felt these kids being young. Before I used to go at least three or four times because I had gum disease. I put that off because of other things. I was running back to Detroit to see my mother, which I should have been taken care of. That was my problem. It wasn’t really the insurance because I do have the insurance, but I was paying for the kids out of pocket because they didn’t have the money.

M: I understand. Thank you, Sarah.

R: Cameron. I normally used to go like twice a year because I had a calcium deficiency as a child. Didn’t have anything done with it until I got old enough to go to work myself. Coming up at my age they didn’t do a lot for me as far as dental was concerned in certain areas of neighborhoods. If you didn’t have any money you were short. I can say in the last five years I’ve spent about $7000 but I don’t see where they’re actually doing anything because it’s a think of drinking milk. They were giving me a calcium shot at one time, but that ain’t doing good, and it didn’t do any good for my other health. When you don’t see the results…most of these dentist are taking your money and not providing good work.

R: They’re not providing information.

R: My name is Sydney. I am fortunate because I’ve had very good experiences. I’ve had very good dentists. One dentist I went to for 20 some years. He passed. All of them have been really good with sitting down and explaining things. I find that…I would say that my dentist, except for the dentist I had, my dentists fire me because I tend to be so uptight when I go to the dentist. I finally found a dentist who said I can’t treat you with anything unless I put you to sleep. That’s helped me a great deal.

M: Thank you, Sydney. Liz wanted to say something?

R: Yes. Liz. Would you phrase the question once more?

M: Sure. How often do you and your family members have dental appointments and why?

R: Thank you. Okay. Liz, again. After you get to be an older person, then…how would you say it…it could be family or it could be…family meaning children, grandchildren greatgrands, or it just could be the individual themselves. It’s hard to answer your question when you think in terms of those factors. As the individual, the economics play such a big important part in whether you will or whether you won’t or whether you can or whether you can’t. Let’s put it like that.

M: I understand. Financially, sometimes you end up having to choose.

R: Yes. My name again is Sydney. I generally go to the dentist. I had gingivitis so I tend to go every certain amount of months. I always go to the teeth cleaning. I think dental work is very important.

R: My name is Adrienne. I do have dental insurance. I use to go every six months for a year, but I haven’t been in a while because I have a lot of other ailments and doctor appointments, so I just have made no appointments to go. I guess I can start going.

M: Adrienne, would you say that your other appointments have been taking higher priority to your dental appointments?

R: Yes, these doctor bills. You name it, I have it.

M: Okay. Do you have access to a dentist or a dental clinic during hours when you’re free?

R: Yes.

M: Sydney, yes. Adrienne, yes.

R: Yes, when we are free.

R: Yes, I do.

M: Will, yes. Cameron, yes. Sarah, yes. Okay. Where do you generally go for dental health services?

R: I have a private dentist. Are you talking about a clinic?

M: Yes, either a clinic or private office.

R: Yes, private doctor.

R: I am Adrienne. I do have a separate dentist with Blue Cross Blue Shield…the Blue Cross Blue Shield dental.

M: It’s a private dentist office. Adrienne goes to a private dentist office. Sarah goes to private.

R: I am Adrienne. I don’t have everything on that. I have like cleaning and extractions…something like that…but just real expensive work, I don’t have that on there.

M: So that’s not covered.

R: When I go there I have to take a pill two days before or three days before for my pressure because I have pressure. I have to take that before I go to him for two days before.

M: Okay. Thank you, Adrienne.

R: Cameron. I have a private dentist but I have MetLife Dental Insurance, which doesn’t cover much of nothing. You’re actually going in your pocket for teeth extraction. They don’t cover anything. Kaiser doesn’t have a dentist.

R: Sarah. Maybe that’s why a lot of people don’t go to the dentist. They can’t afford it and it’s so expensive. For instance, you have a heart problem or some other problem that you see more pressing. You forget the down part and take care and say, well, this is going to save my life. I hear a lot of people saying the same thing…I put off the dentist because it is very expensive.

R: I have Kaiser Comp Insurance and Kaiser doesn’t have dental.

R: Yes, they do.

R: They’ve got dental.

R: Kaiser got dental.

R: When did they start dental because they tell me they don’t have that.

R: I have it.

R: I guess it’s according to what…how your insurance is.

(Everybody talking)

M: So that is something to look into…what is actually covered and what’s not. How easy do you feel that it is to find dentists in your neighborhood?

R: Liz. I don’t think it’s easy at all. Many times you rely on word-of-mouth, a referral by someone else who has had dental work. In our community here we have any number of private dental operations spotted around in the community. We have one clinic that I know of. Further north, Howard University, has a dental clinic for older people that does provide services. By and large it is not easy.

R: Sydney. Right around me there is a dental clinic right on the next block which I’ve been to. It’s a dentist. Right down on Benning Road there are two dental clinics, I guess you would call them now. There’s a wonderful dentist that’s right there on Benning Road, as well.

R: They’re for children, a couple of them.

R: Tracy. Those are all around you, but it’s your insurance, that you have to pay to go into them…see how much they charge. You can’t just walk in because you want to go to the dentist.

M: That’s another thing that we wonder about. Maybe there are resources here, but what types of insurance are actually accepted? Is quality dental care actually accessible to the patients who are trying to receive it.

R: Liz. Now that we’re drawing it out, it begins to look like the dental folk didn’t keep up with the times in so much as with this insurance business, which is very complicated.

R: It’s always been like that.

R: No it hasn’t always been like that. When I was a kid growing up in our community, we had dentists and they were wonderful people, and they wanted to heal you and they would give you…they’d provide the services five dollars, ten dollars, 20 dollars, but not the thousands.

R: This is Adrienne. Dentists used to be free, too.

(Everybody talking)

R: Maria. I remember when I was younger when I went to…there were some schools that had the dental clinics. I happened to go to Merit(?), which had the dental clinic. The kids were brought from around the area to go to that clinic to have their teeth cleaned and to check your teeth and have dental education. All of that was going on back in the ‘50s and ’60s. I don’t know when they stopped that, but they did have that back then which was good because it taught you as a child the importance of taking care of your teeth. I did for years till I got older. To have that in the school was really good because a lot of children took care of their teeth because of that, because they knew the value of that.

R: They learned.

R: Cameron. They did have it in school when I went to school. The only thing is if you had a serious dental problem, like mine…calcium deficiency…they would treat your teeth and put the little red thing in your mouth, but when it came down to serious problems, they didn’t handle those.

M: Okay.

R: That’s where the money came.

R: Cameron. That’s why when you get older if you didn’t get that done as a child now you have to deal with that. That’s where a lot of older people suffer because times changed and it either got to the point where you couldn’t afford it or the government didn’t want to pay for it.

R: My name is Adrienne. I have two filling now. I was about eight or nine years‘ old through the school when they took care of your teeth. They’re still in there. That’s been a long time ago.

R: My name is Will. I understand the liability for most of your dentists. They don’t want to take that…they don’t want…they want to be under a group of doctors. They want to group their liabilities. It’s like medical doctors. That’s why you have Kaiser and Cigna and all these other companies because they’re not going to take just individual responsibility. If they mess your mouth up they don’t want that responsibility be laid on them. They want it to be laid on Kaiser in the group and not the individual.

R: I am Sarah. Why Kaiser if they’re the dentist? They know your record. If you’re going to them on a regular basis, why not put the responsibility on them? I mean, if you’ve got a heart problem and you’re going to get a wisdom tooth pulled and they’ve got to give you some anesthesia, it should be on their part to say, look, we can’t do that because it might affect your heart. Why not it be on them, not Kaiser? The reason I said that is because I just had a neighbor…I’ll get off that because it has nothing to do with dental. She had a stroke. They found out she had a fractured hip. They didn’t know whether to do the operation for the fractured hip because of the stroke. Would we cause her to have another stroke, do we give her some other kind of something to get this hip done? I am thinking if the doctor knows you’ve got a certain problem they’re not going to give you a certain something to do the dental work. I am not sure it should be Kaiser. I think it should be the dentist.

R: Liz. I agree with you because back in the day the dentist took very good care of your mouth. There was a continuity. They know you. Now all of that is kind of vanished and changed. This question of liability and being under a group and all of those kinds of things have come into play.

M: Overall I am hearing that perhaps for the dental services that are received now days are not necessarily meeting your needs.

R: Right. Some of them.

R: Maria. Well, they do still have quite a few dentists out here that are like family dentists or whatever that people frequent a couple of times a year on a regular basis. They do have them out there. I mean, the question about insurance and everything is true, but it still stands the fact that they are available, they’re there. It’s not that they’re not there because they have family practices all over that you could go to. The issue does come up about the insurance and the cost and everything. I don’t think the problem is not having anywhere to go because there are…they’re out there, it’s just the problem of insurance or if you can afford it.

M: Thank you, Maria. Sydney, you agree?

R: Yes, I do. I think it’s a lot financial. I have been very fortunate to have good dentists.

M: That’s a blessing. It is. What other places could you go to if you had a dental emergency, aside from the dentist’s office? Do you know any other places that you could go to for dental emergencies?

R: That’s a good question.

R: My name is Will. I have one option. I am a Veteran and I can go to VA hospital.

R: This is Maria. When DC General was there, you could always go over there for dental emergency at no cost. Howard at one time did that, but now I think you have to pay a fee. You can get emergency treatment at Howard. At least it used to be because it’s been years.

M: Sarah?

R: I was saying are you talking about anytime? If my teeth hurt me so bad or my tooth hurt me so bad at ten what do I do until?

M: Right. That’s a dental emergency, when you’re having the pain for example, or a broken tooth, etc.

R: Sarah. Right. Nobody is open at ten o’clock at night. What do you do? Just take an aspirin or something and wait until the morning.

M: Some people go to the emergency room. Looking at what options are truly out there, especially for those who maybe have to work the next day and can’t get off of work to go and see about it. Some people aren’t able to do that. That’s something we want to look into, what resources are in place even after hours. Tracy, you said something earlier I don’t think we caught. Do you remember what it was?

R: No.

M: Okay. Now we’re going to talk a bit about insurance and affordability. We’ve touched on it a little bit. I am going to skip around here. I would like to have us go around the table. I want to know, do you have dental insurance?

R: Yes, but if you have insurance you have to find out if you have dental insurance on that because some of them don’t call it…don’t carry a dental plan on their insurance. You have to find out. A lot you have to go through. You’ve got to make this call or call them…then it’s according to how much they’re going to charge. If you’re on a fixed income or whatever you may not have the money.

R: Liz. No, I do not.

R: I have a separate one from the government and it’s high option. She did a thing for me and they will pay most of everything.

M: Okay. That’s very good.

R: Maria. I have minimal dental coverage. It’s only going to cover like cleaning and minimal stuff, it’s not going to cover anything like if you have to have implants or stuff like that. It’s not going to cover that.

R: That’s my problem. Cameron.

M: Do you receive any insurance benefits through your employer? I know a few of us touched on this already. For anyone who didn’t have a chance to answer the question, do you have insurance benefits through your employer?

R: When I was working had it and I used to go to the dentist all the time.

R: Did you have to pay for it?

R: When I was working I had dental insurance then.

M: Some employers will actually pay a portion of your insurance premium.

R: This is Maria. I am retired now but I still have through my government retirement my insurance, which has the minimal dental.

R: Tracy. When I left a job the insurance stopped. I picked up other insurance. Now I have to check into that and see. When I was working I had dental insurance.

R: Medicaid don’t cover nothing.

M: James said when they have Medicaid they don’t cover anything.

R: No.

M: Is there a policy in place at work for taking time off to go to dental appointments?

R: I can speak. When I was in the government you took your leave and you went. Just happened the dentist I was going to, if my appointment was at eight, he took me at eight. He had a lot of people, but I was really impressed if they gave you an eight o’clock appointment you didn’t sit there until 8:30 or 8:45. I worked for the government and I had to take my leave. You got this set policy where you can go…not for the government.

R: This is Maria. I agree with Sarah. When I was working in the government that is what you had to do. You had to use your sick leave or annual leave if you wanted to make an appointment…go to an appointment for a doctor…any kind of doctor…dentist or whatever.

R: Sarah, again. Most dentists weren’t open on the weekend. I think they are now beginning to, but on the weekend you couldn’t even go to the dentist so you had to take your leave to go to the dentist through the week.

R: Cameron. Most of the time now you could use your Maxiflex(?)…they give you options. You can take Maxiflex and use that time to go to the dentist or you could come back…not lunch…come back to work. I am on a no lunch schedule. When I’ve got to do something I go do it and just work an extra hour when you come back. They give you that option there that they didn’t give us before.

R: I am at a not-for-profit institution. They don’t pay overtime. If you have to go to the dentist just say you have a dental appointment.

M: For those of you who do have dental insurance, are you satisfied with your current dental insurance plans?

R: No.

M: Cameron, no. Anyone else?

R: Sydney, no.

R: Well, I am because when I had these two teeth pulled…this is Sarah. It cost a thousand dollars and they paid 800 of it. You’ve got to be satisfied with that.

M: Sarah is satisfied.

R: Yes.

R: This is Will. I am in the process of changing companies, so I am going through the process of waiting for everything to fall into place. I know they do have a listing on all dentists. You asked that question before. There is a listing they’re going to give you of the dentists of 20019 zip code. It makes it convenient. I am satisfied at this point.

M: Good. Will says it’s easy because they give him options. For everyone else, do you find that it’s easy or difficult to find a dental provider?

R: I have a list.

R: It’s difficult.

M: Tracy thinks it’s difficult. Anyone else?

R: Adrienne. I like the one I have because he’s a nice doctor. He’s way up northwest, but he’s pretty good. I just need more dental...for doing more things.

M: More coverage?

R: Yes, more coverage. Just need that.

R: Cameron. Some dentists don’t even take Medicaid. I find that kind of annoying because right now I am getting ready to switch over where I have Medicare and Kaiser. What purposes does it serve when you can’t even use it for the things you need to use it for.

M: Good point.

R: I am Sarah. I did say mine was okay because she did a follow-up, a write-up. I think it was going to come to like $7500 and the insurance was going to pay $6800, so that’s pretty good.

M: Okay. Yes, that is.

R: Sydney. I found my first dentist through word-of-mouth. There was a young lady working in his office who became a dentist, so I followed her. I haven’t had a problem finding a dentist.

M: Okay. How did you find your dental insurance for those who are insured?

R: I got mine through the government.

M: Sarah got hers through the government.

R: Cameron. Mine through the government.

R: Mine as well. Adrienne. My son got mine through the government, too, through Blue Cross Blue Shield.

R: Maria, through the government.

M: Please describe what your experience has been like with having your insurance processed by the dental clinic. Everything was okay, Cameron?

R: Yes, mine was okay.

M: Does anyone have any experiences to share about what it’s been like to have your claims processed directly by you instead of the dental clinic? Has anyone had to do that? No. Okay. Has anyone encountered any problems with providers who do not accept Medicaid and the Children’s Health Insurance program? No issues with providers who don’t accept Medicaid?

R: Providers who do not accept Medicaid? Yes.

M: Okay. Do you want to describe a little bit what the problem is?

R: Well, before I got too deep into it I was trying to get some dental work done. I got some advisement from a physician who told me because I was just going into the Medicare area, do not allow them to make Medicare your primary insurance. My employer was trying to do was make Medicare my primary. I would have to pay the difference.

(Everybody talking)

M: What qualities do you look for in a dentist?

R: Sydney. I like ones that will explain what they’re doing in detail.

M: Sarah agrees?

R: Yes.

M: Anyone else?

R: Liz. I like one that’s very patient and calm and calming.

M: Do you have a good enough relationship with your dentist that you trust when he or she advises you about what your dental care plan should be?

R: Sydney. Yes.

R: Trust is very important.

R: Sydney. It is. I’ve had several instances where I have not trusted the dentist and got into a lot of serious trouble.

M: How about you, Carlos?

R: Can you repeat the question.

M: Sure. Do you have a good enough relationship with your dentist that you trust when he or she advises you about what your dental care plan should be? Sarah?

R: My dentist couldn’t take me so he referred me to another dentist. This is the one that says have four and the other one I asked, he says, well, I am going to send you over here to have these two. I said, well, do I need these four and he says, no. They weren’t my primary dentist. Now I’ve got two dentists saying two different things. Of course, I didn’t know what to do. He pulled the two, but I didn’t let him take the other four out because I didn’t know what to do.

M: I understand.

R: And they didn’t give any explanation. You know you want to ask questions and they want to get you out of here, you’re not my patient. He sent me over here…I pull these and you’re out of here.

M: How many of you are currently doing some type of preventative maintenance or dental care?

R: Sydney. The dental cleaning twice a year.

R: Liz. Dental cleaning twice a year.

R: Well, I was doing it three times a year but got behind, so I just need to get back to that.

M: Has your dentist ever talked with you about diseases such as cancer or HPV, cardiovascular disease, diabetes?

R: Mine didn’t. I am Sarah and not a single one of those did a dentist ever explain about your heart, your health…your mouth, but you’re saying your heart and all those other things…cancer…no.

M: Cameron?

R: Cameron. I found out through my regular residence doctor about the problems that your teeth could cause you if you don’t have the care. Also, through my sister. She got a little older and her teeth began to fall out of her mouth because she wasn’t getting property care. Just by people in general. A lot of times when I go to my dentist he’s already talking on me about paying this money to get this implant and doing this and doing that. To me it’s nerve racking, so I take care of my own teeth. I am just paying the insurance right now, which tees me off because I wanted to cancel the insurance but I forgot about it. In the government it goes to the end of the fiscal year and once it starts you can’t do anything about it.

R: When I had a dentist they wanted to know what kind of medication you’re on. As a matter of fact they told me to bring the medicine, they wanted to see it.

M: It’s important to know.

R: Liz. One of the things that I found is that…I guess in a sense I kind of am my own dentist because I start analyzing and looking at what’s going on in my mouth. One of the things I said to the dental technician is…I which I could remember…something about how the acids in the food break down the enamel in the teeth which causes the cavities, which I had picked up not from the dentist but from my own analyzing what was going on between what I was eating and what was happening inside of my mouth.

M: That’s an important connection to make.

R: I am Sarah. The dentists don’t seem to have a lot of time to explain things to you anymore. If you’ve got a tooth that’s got to be pulled, he’s going to pull the tooth but he doesn’t say something else.

R: Maria. Not if you don’t allow it. If you insist on…I say that about any doctor, dentist, whatever. A lot of people just will not ask questions and will not insist on answers. I am not going to let them to do anything to me unless they full answer all of my questions. You’ve got to tell me why and what benefit is it going to be as opposed to this or that. Don’t let anybody just do anything. If you’ve got a question that you really need to be answered, then they need to answer it. If they can’t answer it then you don’t need to go with them.

R: I am Sarah. I agree with you about the dentist and the doctor because I tend to write things down throughout the year what’s going wrong with me from the last time I went. I went to my doctor and I had these four things that I wanted to ask him, which would take one minute. The next time I went he said how many do you have this time? I am very easy going. I said, wait a minute, you’re supposed to be my doctor. I need these four questions answered and if you don’t answer them I’ll find another doctor. She’s right. You have to take things into your own hands. You can’t just let the doctors get away with things.

M: Definitely, it’s good to be your own advocate.

R: My name is Will. Medications you’re taking…I have in my pocket right now the list of medications that I take every day. They’re in my pocket. If anybody asks me what am I taking, it’s in my pocket. If I get in an accident or whatever, it’s in my pocket. I have that information. Another thing, too, like Sarah was saying, you have choices. You have choices in this country. You don’t have to settle for nothing. You can make choices. You can say I don’t want to be with you. I can change doctors. I can change companies. I am paying. You don’t give me nothing. I am going to get charged. It don’t go in reverse. Be active.

R: Proactive.

R: I am Sarah. Doctors now will tell you…my doctor told me he can only take 15 minutes to give me a complete physical. Still when I looked at my bill, it was pretty big.

R: This is Maria. If you can do everything that I need to had done in 15 minutes, that’s fine with me, but you’re going to do everything that needs to be done. If you’re having a physical and you know there are certain things that they are required to do for a physical exam…whether dental or whatever…with dental they do the initial exams to see what’s needed to be done or whatever…whatever it is, dental or medical or whatever, not matter how much time, as long as you know that they’ve done everything they’re supposed to be doing, that’s fine. You can complain if you feel like they’re not doing and you ask them to do this or that, that’s required to be done and they’re not doing it, then you can go over them and say, well, why can’t I get this done. Believe me, you’ll get it done. It will get done. Yes, they do have those…they do that now, they limit their time that they have with the patients now, and it’s crazy.

R: They’re off to the next patient.

R: Maria. Yes, to do that, but you can still speak up though.

R: Sarah. I just got another doctor when he told me he can only give me 15 minutes. This doctor is different. You’re right, you need to speak up.

R: Maria. Yes, you’ve got to speak up.

M: Have you ever missed work before because of mouth pain?

R: Sydney. Yes.

R: Maria. I did years ago. Years ago when I had my first tooth extracted. The pain was woo. I’ve had five children and that tooth pain was worse than having my babies. I am serious. It is terrible. I am not exaggerating. I did miss work because I had to go get that tooth taken care of.

R: Are you talking about one that’s hurting you or just you miss work because you had a dental appointment?

M: One that’s actually hurting you, hurting so bad that you didn’t go to work.

R: Maria. Yes, indeed. I’ve had that.

R: The wisdom tooth hurts real bad.

M: Tracy says the wisdom tooth. Have you ever gone to the emergency room because of mouth pain?

R: I am Sarah. I think I did once. My jaw was hurting. It was hurting so bad I went and the doctor said it was a tooth. It was like at one o’clock at night. I said to the doctor…when the doctor walked in, he just started working on me…I said…I think this is the reason why I got this response…you didn’t wash your hands. I mean, he just came right in and he said how do you know that I didn’t wash my hands. I said there is the sink and I didn’t see you wash your hands. When I left I was still hurting at one o’clock…1:30 at the time…and I said can’t you just give me an aspirin or something until in the morning. They said, no, you have to wait till the morning. I had to wait until the next day. I think I got that response because I told the doctor he didn’t wash his hands.

R: Howard is like that. If you go to the emergency room at Howard they would send you to the dental school.

R: He just gave me a prescription and said they couldn’t give me anything, but I almost suspected because I told the doctor he didn’t wash his hands. I am sorry. You just came right in and started working on me and there was a sink and I didn’t see you do anything.

R: Liz. Just a comment. As I am listening to all my fellow parishioners, I am thinking that…this is a comment now...that many of us have had the best possible worlds as far as dentistry is concerned because my measurement of today’s dentists that I see is old Dr. Johnson who treated me when I was a child…9, 10, 11, 12 years old. His procedures and his approaches stand the test of time. I feel very fortunate to have had that experience because I find that today’s experience leaves a lot to be desired.

M: Anyone have anything to add.

R: Yes, you’re right. Here I go again.

M: Sarah agrees.

R: Yes. I had some dental work done. I won’t name the dentist. It’s been some time ago. I got the laughing gas. A friend of mine’s niece died under that laughing gas. To test my own self…this might sound funny, but this is what I did…I just moved my leg. You can hear the radio and everything that’s going on, but you don’t feel the pain. Under this laughing gas…I was under there too long. I called out my dentist, you got me under here too long, come and get me. What he was doing was going up one, one, one, one, one. I could tell it. At that particular time I told him I am in here, I am your patient now, and I want you to work on me now. I was under that gas too long and thinking about this person that had passed. That just kind of stuck with me.

M: Have you or any of your family members ever lost any teeth?

R: Liz. Yes.

R: Lost any? Yes, I have a granddaughter that’s having a cap put on it…

R: A crown.

R: …yeah, root canal, and she’s only 13.

R: What do you mean lost any teeth?

M: Has it ever fallen out or you had to have it extracted?

R: This is Maria. Yes, I’ve had teeth extracted and I’ve had one that came out.

R: I had one fall out.

M: Carlos had one fall out. Anyone else?

R: Did you say family members?

M: Yes, you or any family members.

R: Liz. Yes. My granddaughter unfortunately has had to have about four teeth removed because her orthodontist said that hereditarily on her father’s side there’s some sort of a weakness or something that means that the teeth don’t grow right. In order to make room they have to take out some teeth.

R: They did that my son like that when he was younger. They had to pull his baby tooth out to make room because his gums…his teeth didn’t grow right in his mouth of something and they had to move some for the regular teeth to come in and the put braces on. It turned out okay.

M: Okay. Tracy said the dentist had to remove some of her grandson’s teeth in order for the other ones to grow in properly.

R: I am Adrienne. My grandson had to have that done. He had the laughing gas twice. That is expensive. His mom had to pay $200 before he could have it done both times. It’s expensive.

M: Do you believe that loosing teeth is a normal part of betting older.

R: Adrienne. It could be.

R: I am Sarah. From what I’ve heard that if you had good dental care it shouldn’t have anything to do with age.

R: Liz. I think I agree with you, Sarah, because I think when my grandfather died he died with all of his teeth, and he was almost 90 years old.

M: What risky behaviors do you think may help to cause poor oral health?

R: Maria. Probably bad choices in our foods that we eat. I think that may help to contribute to poor oral health…things we drink and eat.

R: Liz. I think so. Like drinking sodas and eating excessive sweets.

R: Alcohol.

R: Smoking.

R: Not seeing your dentist.

M: How about sexual activity?

R: Yes.

R: Probably.

M: That’s one that isn’t often thought about, but there’s a virus called the human papillomavirus, also known as HPV.

R: Yes, I’ve heard of it.

M: For those who have HPV it can actually lead to oral cancer in the mouth. Not everyone who has HPV develops oral cancer, but over time, many have. Do you feel that it’s important to eat a healthy diet in order to have good oral health?

R: Yes.

R: Yes.

R: Yes.

R: I think it contributes.

M: Everyone pretty much says yes. Okay. What kinds of foods do you think may increase the risk of poor oral health? I remember Liz told us like sugary sweetened beverages, like sodas, and candy. Anyone have any other ones to add?

R: My name is Will. Overseas there’s something they eat called a Betel Nut. It stains your teeth. It comes out like a tar. They eat it. It’s a plant. It leaves a stain on their mouth. Eventually all those teeth will come out. It gets them high, too.

R: Maria. Now we know why they were eaten it. I was wondering why they would eat it.

(Everybody talking)

R: I think certain food dyes as well as cigarettes, and tobacco, especially will contribute to poor oral health and cancer.

M: These are all really important ones to know…the sugar sweetened beverages, candies, sticky foods. The sugar isn’t good for our teeth. Also, sticky foods can help to trap bacteria and food particles that can end up contributing to poor oral health. Another thing that people don’t think about too often is chewing on ice. Over time this can chip and wear down the enamel and can cause cracks in teeth.

R: Sweetened chewing gum.

M: Yes. That’s why the sugar free version is better in terms of oral health because you don’t want your teeth saturated with sugar. Bacteria also feed on this and plaque can start to form. Liz?

R: Liz. In my self-discoveries I have found recently that brushing my teeth with warm water is…seems to be better for my mouth than cold water because I notice that more refuse or whatever you want to call it comes out of my teeth with the warm water than with the cold.

M: That’s an interesting point.

R: I tend to agree.

R: I agree, too. It’s much better for me to use warm water than cold water…and I have dentures.

R: I agree.

M: Okay. So, that warm water helps to get things off easier than cold water?

R: To break it down.

M: Okay. What kinds of foods or beverages do you think may be helpful in maintaining good oral health?

R: Water.

R: Water.

R: Water.

R: Vegetables.

R: Fruit.

R: Sarah. One thing I heard is that…I like a lot of juices and I was paying attention to the milligrams on the back. When I bought this cranberry juice that says 26 milligrams, I thought that’s not a lot, but that’s 26 milligrams per glass. What I started to do since I liked the cranberry juice, I just mix it half and half. That takes some of the…you still got a little sweet, the color, the juice. You got water and cranberry juice now, but those juices are very…got a lot of sugar in them.

R: My name is Will. That’s a good point. The class I took over at the hospital, they teach you how to read those labels. Reading the labels on cans and bottles is very important…per serving, not the whole bottle.

M: One thing, too, while we’re talking about beverages, sometimes it’s not so much that you should not drink juice or you cannot have any sodas, but it’s important, to brush or at least rinse your mouth out with water afterwards. That can be really helpful for reducing the amount of sugar that’s still in your mouth after eating until you’re able to brush your teeth. That’s also a good tip to help. Are any of you afraid to go to the dentist, and if so, why?

R: Maria. Yes. Don’t want the needle. Terrified of the needle. And then they get to scraping, and uh, I feel nauseous and feel like I want to vomit. I have a terrible experience when I go to dentists. I understand now that they’re putting you to sleep.

R: Sarah. I have had so much dental work. I am not afraid of the needle. In fact, I can just about take the needle. It’s just that since I haven’t been like I should have I am sort of embarrassed. That’s all. I am not afraid of the dentist. I’ve had a lot of dental work so the needle doesn’t even bother me anymore.

R: Liz. Recently when I saw the dentist we talked about the four crevices that I have in my mouth and what we’re going to do about them. He wanted to push implants. I said, okay, I’ll talk with you later, I want to research it myself. I found out that with my physical condition I did not need to have any folks going in my head ‘cause I had had strokes. That just rules that out. I am not afraid to go to the dentist and I am not afraid to pursue other options.

M: Okay. That’s very good. Anyone else?

R: Well, it’s not the needle so much, but one of the later intent to do with the wisdom tooth. I’ve had the needle. It was numb, but when he went to pull it, he pulled me up out of the chair. I want you to know that I can’t go back to him. The pain was that bad. The nerve went all the way up in my head, down my neck.

M: Has that experience and the pain you felt prevented you from being able to go back?

R: Well, by that time I was quite well along. I had some problem with another medical condition. What I want to say about that is that sometimes once dentists find out you may have one medical condition, everything that they talk about has to do with that medical condition because you have agreed to it. I thought what does that have to do with my tooth? Now I have a health professional.

M: All right. Now let me ask you a question. Did that visit prevent you from going back to that specific dentist?

R: Yes, not all dentists, but that specific dentist.

M: To that one. Okay.

R: I am Sarah. Maybe that’s why a lot of people don’t go. The experience of the dentist…I know I need to go, but. I know those needles and I know this is going to hurt, so they keep putting it off and putting it off. Maybe that’s why a lot of people don’t.

M: Does anyone here feel that fear or stress prevented them from going to the dentist?

R: Yes.

M: Maria, yes.

R: Maria. Yes, for real.

M: Is it easy or difficult for you to find transportation to get to the dentist?

R: For me, yes. I am Asian. For me it’s a problem in transportation to get from where I live to way up Northwest, almost to Silver Spring.

R: Liz. I just wanted to share information about the transportation. The Metro Access is very good.

R: Yes, and I ride that. Even I have…what’s the other one?

R: Okay. Good service.

R: What’s the other one you go to?

R: The bus you all use to go?

R: The Episcopal…Seabury.

R: Yes, that’s free. You can ride that free. Seabury, yes.

R: Got another one, too.

R: And there’s WAITS(?)…there’s always…WAITS has been around a longtime.

R: I don’t know about that one.

R: That’s the one that comes a lot through UPO. They have good records for transporting and aides to help you.

R: I need to get that number.

M: Okay. We’re learning extra information here, too, for transportation. That’s good.

**HEALTH COMMUNICATION**

M: Have you ever received information about oral health and preventative care that was connected to other issues like oral care hygiene, smoking cessation, or dietary counseling?

R: Will. Smoking cessation.

M: Okay. Anyone else? No.

R: General nutrition, number one. Number Two…in fact, all of those that you named is included in a lot of health information that goes out to patients and those of us who go out and counsel or have health fairs.

M: Thank you. How do you like to receive information about health? In what ways do you like to receive health information?

R: Sarah. I read a lot and I find out a lot. Some people don’t look at the TV shows. The doctors on TV. There is a lot of health information that comes from those doctors and I read a lot about health.

M: Thank you, Sarah. Anyone else?

R: Liz. I enjoy this forum very much because with the sharing and listening to other people’s experiences and being guided as we move along.

M: Discussion. Exchange of information.

R: Maria. I just take to the internet. When I want to find out about something in particular I hit that internet.

M: You go to different web pages? Do you like Twitter?

R: If it’s something I want to find out about…if someone said you have a possibility of having hyperglycemia. They could explain it to you but you still feel like you want to look it up. Then I’ll go to the internet and put in hyperglycemia and look it up to see what it’s all about. I think those TV shows…I watch them sometimes and I listen to what they’re talking about…different stuff…and I learn a lot looking at those shows, also.

R: Sarah. You don’t even have to have a computer, you can just Google on your cell phone and find an answer.

R: Maria. If you have that type of cell phone.

R: Will. You just listen to Oprah…Oprah, Oprah, Oprah.

R: Dr. .Oz.

R: And Dr. Oz.

R: Tracy. A group like this. You get information like this, that’s helpful.

M: Group discussion. How about you, Carlos?

R: I am still sleeping. Whatever she’s been saying is the same with me. She makes the dinner planning and all that, so I just go along with her.

R: Maria. That’s a husband and wife.

(Everybody talking)

R: About the dentist…she makes the plans…she makes all the plans and I just go by them.

M: She makes the plans and you go.

R: That’s right.

M: Do you ever use more than one communication channel at a time? For instance, television, radio, magazines, newspaper, internet?

R: Will. The way the social media is set up today it’s very informative and it’s 24 hours a day nonstop. What I look at is structure in a lot of these programs so that at the end of the program what have I gotten out of it. When they first start there’s a question, at the end what’s the answer. I am looking for a solution. I think it’s very informative. You have multi choices.

R: Yes, because I had GERD and I was watching one of the television programs and they were telling you which side to lay on to help the GERD. That did help. That was television.

M: GERD is gastroesophageal reflux disease.

R: Sometimes you have to look at who it is you’re talking to and about. One of the things we found that is very informative is you can find out in a neighborhood who is the person that everybody goes to and set up meetings with them. Personally, I have a large family. I could talk to them, they wouldn’t do anything. For prostate cancer and another type of…five different types of cancer, I just made appointments and say you have an appointment and I would take them in groups, and they all showed up. All of the guys got their prostates checked on time, and another time the five types that the hospital used to do, everybody showed up and got their tests. We did that with AIDs and Rural Community.

M: Thank you. In our daily media use do you remember hearing or seeing anything about oral health, whether on television, newspapers, internet?

R: Yes, this is Maria. Television. Also, they send out in the newspaper flyers. They send out cards with dental information for dental offices around the area or whatever, so you do get information in through the mail. Also, the media you see a commercial about a dental office or something. So, you do get the information. It may not be the one you want or an area that you want, but you do get the information.

M: Okay. When you receive this information or when you see it do you pay attention to it or do you ignore it and why?

R: This is Maria. I am not really going to answer because you always know what I am going to say. I get it.

M: Anyone else?

R: I am Sarah. A lot of the advertisements…sometimes they’ll explain about the dental stuff when they’re advertising.

R: This is Hannah. I went to a dentist because I saw the flyer in the area, so therefore I was able to go to the dentist which is where I go now. It worked for me. It may not work for others. I also know I need to go.

M: Flyers, word-of-mouth, television, and the internet. Thank you. Please describe the media message that you’ve seen or heard that have encouraged you to maintain good oral health.

R: Liz. What you’ve done, what you’ve made available to the church is a good example off that…excellent example of that. That’s why I am here.

R: Sarah. I take bottom line…the Bottom Line magazine…and one of the articles they did a three‑page spread on oral cancer which was in depth. That was good. The reading media.

M: How often have you seen any oral related messages that have encouraged good oral health? Have you see it often? Is it sparse? How often do you actually see or hear these messages?

R: On television it’s a lot. You know when they’re advertising their Sensodyne and all that kind of stuff…and in a magazine.

M: Okay. Will?

R: When they advertise toothbrushes, toothpaste, mouthwash. Many of the commercials have it all. It’s a regular thing. It’s constant.

R: Adrienne. They advertise a lot in the stores, different stores.

R: Tracy. Often when you go the doctor and you have a doctor appointment and they recommend you go to an eye exam and stuff. Why don’t they recommend dental.

M: That’s a good point. So often it’s seen as separate, but your oral health can affect every aspect of your health. It’s just as important as any other. That’s a good point, Tracy. We have come to the close of our questions. I want to know, would you be interested in receiving more information about oral dental health for the future?

R: Yes.

R: Yes.

R: This is wonderful.

M: Okay. In which way would you like to receive it? Would you prefer to have more setting like this or would you like to just have a flyer or website?

M: (Everett) A brochure would be helpful?

R: Do you have brochures?

M: We would develop them.

M: (Everett) Well, we can develop our materials. We’re looking at things that if you would really be interested in seeing, so brochures would be an option, basic flyers, something that you really feel is something that would be very beneficial to you.

R: I think brochures.

R: I think that a mixture of media is helpful. People get tired of it. They will take a brochure and it comes in the mail and after a while they stop reading it. The same thing happens with magazines. If you can get a variety so that people can change around and exchange and share with somebody then I think you’ll read a larger number.

R: Liz. Howard University produced a fantastic film for children on dental health. It is fantastic. It was cosponsored by the Linx Organization. That might be something you might want to look into. That would be just wonderful even for adults to look at.

M: Thank you.

R: More communication is good because what got me was when I found out it was Georgetown University. Then I knew it was bona fide.

M: The source is important.

R: Source is important.

R: What about also having a dentist come in who could even have slides or something to show exactly what this oral stuff is…the cancer and what happens. I know when I go to the dentist she’s always probing and looking to make sure that everything is in order. I think that hands-on is very, very important and that you see what’s out there and how you think something is small, just a simple cavity, what can it erupts into.

M: We actually have two dentists on the ground. This portion of the study right now is getting the information from you, but what we really want to do is have those extra resources in place to benefit the community. Right now we’re benefiting from getting this information from you so we can tailor those programs.

R: It’s a grant that you...is only for adults?

M: Yes, at this time the focus is adults ages 18 to 74.

R: I belong to a prostate survey for nine years. That’s how I met him to select. They’re still sending me information. What they had (inaudible) do was what you see in multiple vitamins called Selenium. That’s still going on, but that does nothing for the prostate. That’s why they closed the survey down. They’re still sending me information on it and I appreciate it because it really helps…any information that you get that’s going to be beneficial to you. That’s what I found.

M: Knowledge is power.

R: Yes.

M: We thank you so much for coming to talk with us today. We truly appreciate all the feedback, the discussion. It will definitely help us to tailor a program and get resources in place that will help the community.

// end of recording //

Beverly
