# Supplementary material for: A qualitative study of oral health knowledge among African Americans
Source: PLoS One. 2019 Jul 10;14(7):e0219426. doi: 10.1371/journal.pone.0219426 (PMC6619789; doi:10.1371/journal.pone.0219426)
Supplement: S3 Text — This is the Church 2 focus group transcription. (DOC) [file pone.0219426.s003.doc]

**GEORGETOWN-LOMBARDI HEALTH DISPARITIES INITIATIVE**

**Oral Health Focus Group Transcription– Church 2**

**April XX, 2016**

**Project #GGT0421-16**

**ICE BREAKER**

M: Ok, let’s started. I’d like for us to go around…please use your pseudo names that we handed out earlier and tell us one thing that you like to do as a hobby. We’ll start with Tracy.

R: My name is Tracy and as a hobby I like knitting and sewing.

R: My name is Sarah and as a hobby I like making jewelry, sewing…all kinds of arts and crafts.

R: My name is Tiffany. My hobby is sewing.

R: My name is Adrienne. I like to solve puzzles as my hobby.

R: I am Ellen. I like reading and music.

R: I am Karen. I like shopping and traveling.

R: My name is Carlos and I like (inaudible).

R: My name is Anita. I like running, fitness.

R: Jordan. I kind of hate saying it. It’s a man’s name.

R: No, a lady can be Jordan.

R: Okay. I like music and solving puzzles.

R: My name is Shawn. I like making money.

R: That’s a hobby?

R: That’s a hobby. That’s my hobby.

R: My name is Liz and I like to dance.

R: My name is Anthony. I love sports and music.

R: My name is Susan and I like word puzzles.

R: My name is Alex and I like golf and focus groups.

M: We’re going to be contacting you. (laughter)

**ORAL HEALTH**

M: My first question to you, can you tell me what comes to your mind when you hear the term oral health? What do you think about when you hear that?

R: Cancer. That’s my first…oral cancer.

R: Flossing.

R: And then dental health.

R: Taking care of your teeth.

M: Thanks, and so we know what you’re saying, before you answer, please use your pseudo name. Carlos?

R: Making sure you take pride in your body and everything…take good care of both.

R: I am Tracy. When I hear oral health the first thing I think about is your mouth, completely. Your complete dental care.

R: My name is Jordon. I think about care, care of your teeth and gums.

M: Thank you. Do you think that oral health problems are as serious as other health problems?

R: Jordan.

Rs: Yes. (multiple)

M: We seem to have consensus. Karen, you were saying something, too?

R: Yes. I am Karen. I think so. I think it’s just as important.

R: Tiffany. I think so.

R: My name is Tracy. On TV there was an ad with in going to the doctor the first thing the guy was told to do was first get his dental health taken care of because that effects a lot within his body.

R: Right.

R: Yes, it does.

M: And that’s the thing. Many people don’t realize that because for so long dental and oral health has been seen as its own thing, separate from the rest of the body, but it’s really not. You guys are on point.

R: Carlos. I think what you don’t know and what you don’t check on it could kill you. I think it’s always best to be concerned.

M: Great point, Carlos. Do you know of any connections to other health problems that can come from having oral health issues?

R: Alex. No. And to the previous question, I agree, oral health is important. I’ve never heard a stage four blossom oral…not paying attention to oral health. I’ve heard of stage four…tons of cancers, but never connected to oral. Lot of things are important. Is it as important as the other one, I don’t know.

M: Lots of things. We don’t really hear about it as often.

R: Right.

R: Right.

R: My name’s Tracy. It affected a lot of other parts of your body because if you don’t chew your food properly it affects your digestive system.

R: Alex. Right. I think that’s the point I was trying to…maybe there could be better communications from the medical environment to say this is why oral health is important for these other reasons.

M: We have agreement with Alex on that point from several people. Carlos?

R: I think your kidneys are the one of the most important organs in your body because you have to drink a lot of water. If you don’t take advantage of water it can damage your kidneys.

M: That’s true. In terms of connections with other health problems and oral health, you can end up having an infection that goes to your heart because of issues in your mouth. If you have an infection in your mouth, it can also spread to your brain. A few years ago there was a young boy named Deamonte Driver who had a dental infection and abscess that wasn’t treated and he was having a lot of pain. Over time the infection actually spread to his brain and he died. It’s most certainly connected. There was also a young girl in Texas…14 months old…who was being treated for cavities and she died after the procedure was over. It’s not certain at his point what caused her death. At 14 months old she had cavities that needed to be filled. She hadn’t had teeth for very long. Oral health can have huge implications on your health in general and your life regardless of your age.

R: Hi, I am Liz. I remember reading something in the paper a while back…a young boy in D.C. The mother could not afford healthcare and he died. So, in some way the community needs to make healthcare more affordable.

M: Yes. It can be really expensive even with insurance.

M: What is the importance of oral healthcare in your household?

R: I am Sarah. We brush our teeth every day and we brush down on your gums and everything like that and floss your teeth and everything because your mouth carries so much bacteria from the food and stuff. If you don’t get your teeth scaled at least once a year that bacteria is building up and sometimes you can actually smell it. Sometimes people open their mouth and you can smell it from their breath. That’s the bad stuff building up in their body.

M: Thank you. Carlos?

R: One of the most important parts of oral health in my household is washing your hands because your hands carry germs and bacteria. You don’t know if you serve somebody food all the bacteria can make the other person sick or if you work in a fast-food restaurant, your hands play a big part.

M: Ok.

R: My name is Adrienne. I think the best thing is cleansing. Take care of everything. No halfway dirty, all the way clean. Make a habit of it.

R: Shawn. There’s a commercial. You guys seen that commercial where the man’s in the bathroom and he comes out of the bathroom and he’s tucking down his thing and he walks over to his friend, pats him on the back and says have a good day. That makes you think.

M: It is surprising sometimes what people do.

R: Or don’t do.

M: What kind of oral care regimen do you have in your household? What’s your routine regarding oral healthcare?

R: The first thing I do is make my bed up when I get out of it. I got my little spray I spray in the bathroom. I disinfect the whole bathroom when I finish.

M: Ok. What about your oral care regimen?

R: I brush my teeth.

M: Okay. Anyone else?

R: My name is Tracy. The first thing I use when I go to the bathroom is use the mouthwash. Sometime I use the dental floss or whatnot, and then I brush my teeth. You know you’re supposed to brush for so long, and that’s what I do.

R: I am Karen. I was going to say the same thing. I keep a big bottle of Listerine and I use it.

R: I am Sarah and I have what you call a tongue cleaner.

M: A lot of people don’t use those. Those are important.

R: That’s a good thing. Yes…a tongue cleaner.

R: Sarah. I clean my tongue. Your tongue holds a lot of…even down to a baby. Have you ever looked at a baby’s tongue? The doctors tell you, clean that stuff off the tongue.

M: Anyone else?

R: Yes. Alex. I floss in the morning, brush teeth, and use mouthwash. I also brush my tongue with the toothbrush.

R: It’s important to use the mouthwash after you eat your breakfast because you still have that stuff in your mouth.

M: How often should you and your family members have dental appointments and why?

R: I am Susan, and I think your dental appointments should be six months.

R: Once a year.

R: Some people are every six months.

R: Shawn. Unfortunately a lot of people don’t think to go to the dentist unless they have an infection or need to have their teeth drawn or something like that. Otherwise, they just ignore it completely until they have an emergency.

R: I am Liz. A lot of people do not go because they simply cannot afford it.

R: Some people don’t go because they are afraid of the dentist.

M: Do you have access to a dentist or a dental clinic during hours when you’re free?

R: I do, yes.

M: Shawn does, Jordon does, Liz, yes, Anthony, yes, Alex, yes, Anita, yes. Shawn?

R: The good thing about it I have a dentist now…when you were younger you were afraid to go to the dentist because they would stick you before they pulled your tooth, and that hurt. Now I have a dentist who is pain free. Everything is absolutely pain free. He can pull your tooth and you don’t even know he’s done it. The man’s a genius.

R: I have never met one of them.

M: I would like to get that information.

R: Shawn. You know when I was young you’d go to the dentist and he’d stick in the needle and that thing hurt, and then when he pulled the tooth it hurt, but now when you go he does something, you go in, he waits a minute, he comes back, he gives you five or six needles, you don’t even feel them, pull your tooth, and he says it’s done. I even walk out of his office pain free.

R: They numb you first. They give you that pink stuff.

R: That blue stuff.

(Everybody talking)

M: Where do you generally go for dental health services?

R: My personal dentist.

R: Your private dentist.

M: Private dentist. Sarah, private dentist. Ellen, private dentist. Liz?

R: I tried Howard University Dentist School, Medical Dentist School, at one time. It was great.

R: I did, too, at Howard University.

M: Jordan, also? Private dentist.

R: Shawn. Private dentist.

M: Shawn, private dentist.

R: Shawn. Then I got out of the phone book. Best thing that ever happened.

M: Gives you a lot more options, right, where to go? How easy it is to find dentists or dental clinics in your neighborhood?

R: Easy.

R: It’s easy.

R: It’s not too hard.

M: Anyone else?

R: Susan. I prefer a reference because I don’t trust everybody.

R: I am Sarah. We work for the government so you know they have the healthcare thing so you have a choice of going through the whole system. My job would be to pick your own dentists and doctors and stuff.

M: How far do you have to go for dental health services? What distance do you have to travel?

R: I come from Maryland to D.C. to go to my dentist, and that’s a good ten miles. But like I said, because he’s so good and pain free, I don’t mind doing that, not at all.

M: How about everyone else? How far do you have to travel?

R: Not far.

R: Twenty minutes.

R: I have to travel all the way to Maryland…the Langley Park Borough. It’s not that far. It’s in bus route and I catch the Metro.

M: So, it’s easy?

R: It’s easy.

R: I was at the dentist yesterday. I am getting a new set of teeth. I didn’t like it at all.

R: At least she can chew up all her food now.

M: That’s great. For those of you who have children, do you and your children go to the same dentist?

R: Anita. Yes.

M: That’s Anita and Alex, yes? How well does the dental clinic where you go meet your needs? Do you feel that your needs are met when you go to the dentist?

R: Oh, yes.

R: Alex. Very well.

R: Anthony. I do.

M: We have a pretty good consensus, yes, the dentist meets your needs. Do you know of any other places where you could go if you had a dental emergency other than your dentist office?

R: No.

R: The hospital.

M: Tiffany says the hospital. Anyone else? We have a few people agreeing with Tiffany.

R: Yes, Emergency Room.

R: Shawn. I’ve even had my dentist make an appointment for me on Sunday. He said you can come it, it will just be me and you, we can pull your tooth, take pictures, or whatever, on a Sunday. He took me.

M: That’s great. A lot of dental clinics are closed on weekends. I would like to have your dentists information. Do you and your family have dental insurance?

R: Shawn. I don’t.

R: Yes.

R: Yes.

R: No.

R: No. Liz. I just heard about a dental insurance, that we could get it through the federal government. I think I have to do it during open season. The next open season in November I am going to get dental insurance this time around.

R: Susan. I have dental insurance through my health insurance…an Advantage plan, and dental insurance is in it.

M: Okay. Are you currently working?, and if so, full-time or part-time?

R: I am Sarah. I work full-time.

M: Full-time, Sarah.

R: Part-time.

M: Anthony, part-time.

R: Full-time.

M: Carlos, full-time.

R: Susan’s retired.

R: Ellen, retired.

R: Shawn’s retired.

R: Liz is retired.

R: Jordon’s retired.

M: Retired, Jordan. I know we touched on this a little bit but do you receive any insurance benefits through your employer?

R: Yes. Alex.

R: Oh, yeah.

R: Anita, yes.

M: Okay.

R: Shawn. I’ve been retired two years, but we have this plan for retirees. They give you $1000 a year towards whatever you use it for, for medical. If you don’t use it, it goes on to the next year. They give it to you each year. That’s how I pay for my dental. I can go to a doctor or a dentist and use that card and it will pay for it. It’s something they give you as a retiree.

M: That’s great. It actually rolls over. Is there a policy at work for taking time off to have dental appointments? Carlos?

R: We use our leave.

R: I use that too.

R: Alex. We have flextime.

M: I know some employers have a policy where you can take time off, but other people are not able to. It ends up being a big issue, so we wanted to know about that.

M: How do you feel about the money that you would have to spend out of pocket, whether at the dentist’s office or a dental clinic? Any experiences with that, the amount of money that you have to pay out-of-pocket?

R: No, my insurance covers that money.

M: That’s good, Anthony. Nothing comes out of your pocket.

R: I am Tracy. I’ve been fortunate in having a dental plan through my employer so I spend very little money out of my pocket.

R: I am Tiffany. I don’t have dental insurance, so when I go I have to pay quite a bit of money.

M: How do you feel about that?

R: I don’t.

M: Anyone else?

R: Alex. I’ve got insurance and it covers a hundred percent of preventive maintenance for two times a year, et cetera. Anything that requires orthodontia I have healthcare that comes out of after tax money. You basically have to know…you have to have a separate savings account for that. I am sorry, it’s pretaxed.

R: I don’t pay for nothing but my medicine, but they’re checking on that now. I might get that free.

M: That would be good.

R: It should be. I was in there long enough and paid enough taxes.

M: Now do you know what’s covered by your dental insurance? Alex says yes.

R: Yes. They give you all that at the health fair thing.

R: Susan. What’s covered is your cleaning, X-rays, and probably extractions, but if you have to get something else done you might have to pay.

R: You don’t pay the full amount…should pay half and you pay the rest.

M: Okay. Are you satisfied by your current dental insurance plan?

R: Yeah I’m satisfied.

M: Anthony is satisfied. Is everyone else satisfied by their current dental insurance plan?

R: I don’t have a dental insurance plan but I am satisfied with my dentist.

R: That’s important. I am satisfied with Kaiser.

M: Okay. Was it easy or difficult for you to find dental insurance?

R: Liz. I think it would be easy once I go through my…I am with Kaiser. Once I go through the book I am sure I can find a dentist who is union the Kaiser plan.

M: Thank you. Anyone else?

R: George Washington University has a consulting office for medical care. They will send you a list of dentists or dermatologists, which I have got from them.

M: Okay. Carlos?

R: It was easy for me.

M: Please describe what your experience has been with having your insurance processed by the dental clinic or your private dentist.

R: No.

M: No. Okay. Good. Has anyone encountered any problems with providers who do not accept Medicaid? No?

R: What do you mean? I don’t touch Medicaid because I don’t like Medicaid. Medicaid in the long run is tricky. They want your land or your house or something.

M: Really? I haven't heard about that.

(Everybody talking)

M: What qualities do you look for in a dentist?

R: My name is Tracy. I look for a dentist who has all of the modern equipment because I do not want to be hurt.

R: I am Sarah. I look for a clean dentist.

M: Carlos?

R: I look for a dentist that’s concerned more about you than a dollar bill.

R: I like my dentist. They relax you and they’ve actually got a TV screen on top of where they do your dental...

M: It’s a nice distraction, right? Anyone else? What qualities do you look for in a dentist?

R: Susan. I like when I get there for them to be ready for you. I’ve been to dentist where I sat for an hour before she was able to come, and that’s inconvenient.

R: Shawn. They ask you to make the appointment…to come at four…and they don’t get you to you till 5:30.

M: You want a dentist that is on time?

R: Exactly.

R: I was in one yesterday and it was beautiful. Everything was clockwork.

M: Is it easy for you to schedule a dental appointment?

R: Yes.

M: Ellen?

R: Yes, and they call you the day before you are to come to remind you of your appointment.

M: That’s really helpful.

R: Yes (multiple)

M: Do you have a good enough relationship with your dentist that you feel you can trust when he or she advises you on treatments or health plans?

R: I do.

M: Shawn does. Tiffany does. Liz does. Susan does.

R: Yes, I do.

M: Everyone pretty much is able to trust their dentist. Do you feel that it is easy or difficult to talk to your dentist about your care?

R: Yes. Liz.

R: Yes. It’s easy

R: Yes.

R: Shawn. Mine’s easy.

R: Mine’s easy.

M: Okay. Jordon’s is easy, too.

M: After each visit do you feel that you know better how to take care of your teeth and gums?

R: Yes.

R: Yes.

M: Okay. We have consensus on that.

M: What has your dentist ever told you about things that you can do to have good oral health?

R: Adrienne. Have good teeth.

R: No, oral health.

M: What has your dentist every told you about things you can do to have good oral health or good dental health.

R: Things that take care of your teeth.

R: My name is Tracy. One of the things that my dentist does is he usually gives out the toothbrushes and other things that you can use to take care of your mouth or whatnot.

R: Ellen. They tell you what toothbrush to use, tell you what part of your mouth where you should brush more than the rest of your mouth because tartar will build up there. They’ve told me that.

M: Thank you.

R: Liz. My doctor mentioned flossing every day.

M: Okay. Jordan?

R: The last time that I went he gave me a little brush which I had never had before. I’ve had the long brush with the little brush on the end. This was a real short one I could carry in my pocketbook. I have one. I can use that easier than I can flossing. I have a hard time with flossing. They’ve showed me how to do it. The toothpicks with the little floss on it, I can use that better. I just can’t operate it, so find the little brush does. I meant to ask him the next time I go if it’s all right if I don’t floss and use the brush instead.

M: Has your dentist talked to you about pulling versus saving teeth?

R: Yes.

M: Anthony, yes.

R: Susan. Most dentists prefer to save your teeth but the method that they use I don’t agree with. I don’t believe in root canals. I am not a doctor. I think I prefer pulling, but they try to save your teeth…most of them.

R: I agree with you, get it over with.

M: Carlos?

R: Some dentists tell you what they eat and what they don’t eat that will attack your teeth.

M: When you’ve been to the dentist how do you feel that the dentist treats you?

R: Oh, nice. He has a good bedside manner.

M: Adrienne feels the dentist treats you well.

R: All of them…the doctors, the nurses.

R: Shawn. I do, too. Yes, it’s good.

M: Everyone is agreeing. Okay.

R: At Howard University you have a student the whole time with him, so of course he’s like your private doctor.

M: Has your dentist ever talked with you about cancers, HPV, cardiovascular disease? Any of these?

R: Yes.

M: Your dentist has talked to you about it?

R: No.

R: Sarah. Yes.

M: Anyone else? Has your dentist talked to you about any of these other health problems?

R: Only one time.

M: One time, Adrienne.

R: I had a toothache and I drank some liquor.

(Everybody talking) (laughter)

M: Anyone else? Has your dentist talked with you about these other health problems, whether it be cancers, HPV, or cardiovascular disease?

R: No, never.

M: No, never.

R: My name is Tracy. He has talked to me about because usually before I could have any dental treatment I have to take off medicine.

M: Okay. Jordon, Shawn, Liz, and Anthony said no, never. Your dentist has never talked to you about any of these other health problems?

R: No.

R: Shawn. The only thing my dentist did was when I was seeing him one time when he went to take my blood pressure. My blood pressure was high. I can’t pull your tooth. You have to get your pressure down. This tooth can’t come out with your pressure as high as it is.

M: Liz?

R: Yes. My doctor did mention something about cancer, but it didn’t really…he wasn’t really referring to me, was just making a statement of the relationship between cancer and your teeth.

M: Okay.

R: Ellen. If you have a cold they tell you not to come.

M: Okay.

M: Have you ever been screened for oral cancer or HPV? HPV is human papillomavirus.

R: Yes. I think we get the oral screening here at St. Martin’s when Georgetown…Howard University come in. We get it here. We get the oral screening. They just swab the mouth. I believe that’s part of oral screening.

R: Shawn. Which is about once a year they come.

R: Yes, we get that here at St. Martin’s.

R: Shawn. Yes, once a year.

R: When we have a health fair.

R: Shawn. The health fair once a year they come and they do that, and they almost insist that you have it done while they’re here.

R: I’ve never had it done. I have to remember to do it next time. I’ve never had that.

R: The HIV test where somebody puts a Q-tip on your mouth.

M: Okay. You’ve had HIV screening, not HPV.

M: Has anyone here ever missed work because of mouth pain?

R: No.

M: No one’s missed any work because of pain in the mouth? Okay. I will skip over these next couple of questions.

R: Shawn. I had that one time when I was home. I was in such pain. This dentist was so…I called him and I was crying. What he did was he actually called in a prescription for me to ease the pain. I went and picked up this prescription. All this green stuff came out. It cleared it up. It cleared up between that time and the next time I had to go see him for my next appointment.

M: Has anyone ever been to the Emergency Room because of mouth pain?

R: No. (multiple)

M: Okay. Have you or any of your family members ever lost any teeth?

R: Yes. Jordon, Susan, Shawn, and Liz and Anthony.

R: I lost teeth on a piece of candy. I was chewing on my own teeth.

M: Carlos?

R: My wisdom teeth.

M: Okay. Anyone else? Karen?

R: Oh, yes, I have.

M: Yes. Now has losing teeth affected your chewing or digestion?

R: Yeah.

M: Jordon says yes. Anyone else?

R: Adrienne says yes.

R: Tiffany, yes.

M: Do you believe that losing teeth is a normal part of getting older?

R: Everything is because of getting older.

R: Tiffany, yes.

R: I lost most of my teeth…the ones that are gone…in my younger years, so I don’t think it’s getting older.

M: Anyone else? Any other thoughts on that?

R: They do say that women during pregnancy, someone can lose their teeth.

R: I had some back teeth that just falled out when I had chewed.

R: When you’re older you lose your teeth.

M: Ok. You think it comes along with getting older?

R: Yes.

M: Do you believe that there are any risky behaviors that can contribute to poor oral health?

R: Could be.

R: Tracy. Yes. If you don’t take care of your teeth.

R: Sarah. I know when I have sinus problems real bad my teeth would ache so bad I can’t see nothing. I had a blacken out from it. I had to go to the dentist and he had to put something to numb the gums so I don’t feel the pain.

M: The maxillary sinus sits right above your teeth so when you do have sinus pressure sometimes it can press on the nerve of your teeth and feel pain.

R: I couldn’t lay my head this way. I couldn’t lay my head that way. The next thing I know I was walking to work and I fell right on the street. He said it was from my sinuses.

R: You name it, I’ve had it.

M: What about smoking? Do you think that smoking is a risky behavior that can contribute to poor oral health?

R: Yes.

R: Yes, it does. Smoking and caffeine together.

R: I can’t say that because when I was smoking my teeth used to stop hurting.

M: We have a lot of yeses. How about drinking?

R: Drinking, too.

M: Can drinking contribute to poor oral health?

R: Sarah. Yes, drinking can, too.

R: Shawn. Yes, because it stains your teeth.

M: Drinking and smoking can both contribute to poor oral health…especially together. They can cause oral cancer over time.

How about sexual activity? This one doesn’t seem obvious. With sexual activity if someone becomes infected with the HPV during sexual activity the HPV infection could actually cause you to have oral cancer over time. Not everyone who has HPV will get oral cancer, but some people do, along with other cancers…cervical, anal…

R: My husband had cancer of the mouth, of the tongue, and his teeth were…it was just sad, period.

M: I’m very sorry. It’s a very difficult situation to be in.

R: It’s bad.

M: I want to note that there are many different causes of cancer.

R: Sarah. They had a program one day. Two girls, elementary school age. They got sick and their parents took them to the doctor. Come to find out they had gonorrhea in their mouth. They wanted to know how they got gonorrhea in their mouth. These girls in this school were doing oral sex.

M: Definitely sexual activity is a risky behavior that can cause oral health problems. Do you feel that it’s important to have a healthy diet to maintain good oral health?

R: Yes.

R: Yes.

M: We have lots of yeses. What kinds of foods do you feel may help you to maintain good oral health? Carlos?

R: Junk food.

M: That would be for poor oral health.

R: Carlos. Oh, you said good health. I’m sorry about that. Salad. Wheat bread. Fruits and vegetables.

M: Sure, defintely.. Getting that fiber.

R: Right. And protein.

M: Yes, lean protein.

R: Yes.

M: Anyone else?

R: Calcium.

M: Yes, calcium. Very good for your teeth. Can anyone think of a beverage that might be good for maintaining good oral health?

R: Water.

R: Susan. Milk.

M: Water and milk. Yes.

R: Cranberry juice. Very important.

M: Ok. We’re going to get into foods and beverages that might cause poor oral health. Sugar sweetened beverages are a part of that. Juices often fall into that category because of the acid. It doesn’t mean that the juice itself is bad, but we shouldn’t let the acid or the sugar sit on the teeth because over time that can ruin the enamel. In addition, bacteria feeds on sugar. Once they start colonizing, they help to cause plaque and tartar, and can also affect the breath.

M: Any ides on other foods or beverages that may cause poor oral health?

R: Susan. I know that candy can do that, but as far as food goes some fruits can do that too…fruits that have acid, such as oranges and lemons and so forth.

M: That’s the thing. Even though fruits and vegetables are good for our health in general, we have to be careful after having…whether it’s fruit, fruit juice, things like that…anything really after eating…we should make sure they don’t remain on the teeth because the sugar and acid can damage your teeth and gums. What other foods and beverages do you think may contribute to poor oral health? Carlos?

R: A milkshake. That can contribute because it has a lot of sugar and it’s fattening.

R: Too many bananas. I am Karen.

M: Do you feel that you or your family, in general, have access to enough of the kinds of foods and beverages that would help you to maintain good oral health?

R: Yes, I do.

R: I think so.

M: Everyone is saying yes. Okay. Do you currently have regular dental checkups?

R: Yes.

R: No, I don’t.

R: I do.

R: No, I don’t.

M: Anthony, Liz, Jordon. Okay.

R: Susan. Not like I should.

R: Shawn. No, not like I should either.

R: Need to.

M: Okay. Ellen does. Okay. Are you afraid to go to the dentist?

R: Yeah.

R: Shawn. I used to be but not anymore.

M: Shawn used to be but not anymore. Ellen, no.

R: Sarah, no.

M: Has anxiety or stress ever prevented you from going to the dentist?

R: No.

M: No. Okay. Is it easy for you to find transportation to get to your dentist?

R: Yes.

M: Everyone says yes on that. Is it easy or difficult for you to get time off from work in order to go to your dental appointment?

R: Easy.

R: We wouldn’t get no time, we just took it off.

M: I’ll skip this one.

**HEALTH COMMUNICATION**

M: Have you ever received information about oral health and preventative care that was connected to other issues like hygiene, dietary counseling, fluoride applications?

R: No. (multiple)

M: How do you like to receive health information? Options could be like the TV, email, flyers, pamphlets, going to websites, text messaging.

R: Anywhere.

M: How would you like to receive oral health information?

R: In the mail.

M: In the mail, like pamphlets. Jordon says in the mail. Carlos, in the mail. Sarah, in the mail.

R: Group meetings.

R: I agree in the mail.

M: Karen says group meetings.

R: Susan. I agree with the mail.

R: I don’t give out a lot of my email, even though I have a computer because I am not one of those that’s going to sit at the computer every day. Well, I do every day but just for a short length of time. To keep receiving stuff online causes you to be at the computer so long. That’s not a thing I do.

M: Okay. You’d rather have that in the mail.

R: In the mail. Then I can read it when I feel like it.

M: What types of communication do you pay attention to the most?

R: All kinds.

R: TV, pamphlets.

R: Shawn. Television.

M: Shawn says television.

R: Pamphlets.

R: Pamphlets, TV. Anyway I can get it.

R: Susan says pamphlets.

R: Adrienne. Unless it hits me in my body, I never worry about it on the TV.

M: Unless it affects you directly.

R: You don’t think about it. Sometimes the mind brings on a lot just looking and listening.

M: That’s true. But then knowledge is power, too. We need to get the information.

R: You’ve got to have a mind…just like smoking. If you want to give up, you give it up. When I think something is going to bother me, I give it up. I don’t care what it is. I don’t touch it.

M: Finding that strength.

R: You have to have the will power.

M: Ok.

R: Yes, because you’re shortening your own days. Nobody ain’t doing it but you. I smoked when I was…I don’t know…when it started getting high price I said it’s time to quit.

M: In your daily media use, whether through the TV or pamphlets, text messaging, do you recall seeing or hearing anything about oral health?

R: Adrienne. Every day.

R: Yes.

M: Every day.

R: They’re selling something.

R: Shawn. The TV media because they always talking about ProNamel.

R: About smoking.

M: Carlos says about smoking. Ellen?

R: They send you pamphlets about where you can buy this kind of teeth…discounts.

M: Those promotional pamphlets in the mail. How about Tiffany?

R: No.

R: Different things on TV. On Channel 26 when they have health things and different stuff like that.

R: Tracy. There’s an ad that’s on the radio. These dentists up on Fenton(?) Street advertise that they’re open Saturday, Sunday, all these different hours, and all you have to do is call and come up there…up in Silver Spring. They say Saturday they have late…Sunday, whatever.

M: Fenton Street.

M: When you see messages about oral health do you pay attention to them or do you ignore them?

R: Pay attention.

M: Liz pays attention, Shawn pays attention, Jordan pays attention…Carlos.

R: I always look at something.

M: Does anyone here ignore messages that they see or hear about oral health?

R: I do.

R: You can’t ignore it.

R: Certain advertisements when they talk about clear choice and different things for your teeth. If you don’t feel like you need it, you don’t pay much attention to it.

M: For those who have seen or heard messages about oral health, can you describe some of the messages you’ve seen that encourage you to maintain good oral health? Any messages that stick in your head that you remember that encouraged you to maintain good oral health? Liz?

R: Yes. Whenever they speak of people with sensitive teeth and they recommend different types of toothpaste to use. I am all ears on that.

M: Anyone else? Carlos?

R: Mouthwash to freshen up your breath.

M: Okay. Anyone else? Do you recall any messages that you’ve seen or heard that encourage you to maintain good oral health?

R: Quite a bit, yes.

M: Do you remember any of them? Sarah?

R: I remember one where they showed the proper way to brush your teeth and everything. They went through the whole program about it. And then, they showed how some people lose their teeth, especially those who put tobacco down in here. They’re talking about the whole dental oral thing, about your mouth and stuff, what caused bad dental health. They showed about smoking and drinking, and then especially about the baseball players…

M: Chewing tobacco.

R: Yes…the gums and stuff…how to get cancer.

M: A lot of people didn’t know about that. People were doing it for years.

R: Shawn. And snuff.

R: Yeah, snuff years ago.

R: They’ve got something new out now. I think some kids know about it.

M: I’ve heard about a new chewing tobacco product too.

R: It looks like candy. It messes up your mouth. It’s almost like that. They say it might have like a nicotine or something in it. I don’t know where the kids get it. They know more about it.

M: Yes, I’ve heard of it. I don’t know exactly what it is.

R: It almost looks like candy.

M: It’s like a new age snuff?

R: It’s a new thing, yes.

M: We have come to the end of the focus group itself. Would you like to receive information from us about oral health, like in a pamphlet or a brochure? Would you be interested in that?

R: Yes.

R: Sure.

R: Liz. Yes.

M: Okay.

R: Shawn. I would, too. Because sometime they may ask you if you want to participate in a free study that they could help you that you don’t have to pay for. I am all for it.

M: Okay. And then also on that note, would you be interested in us contacting you about future studies? In the Office of Minority Health at Georgetown University we also have studies on breast health. There’s the Capital Breast Care Center that offers breast screening, mammograms, things like that, regardless of a person’s ability to pay. There are also other studies that have to do with prostate health, nutrition, and exercise and fitness. We have a variety of studies. If you’re interested in not only those but future studies that might apply to you, would you like for us to contact you?

R: I would. That’s why I gave my email address.

R: Liz. I did not give my email address but I would like to…especially fitness and exercise.

R: I get all that information already.

M: Great. We’ll pull your form back. Anyone else would like for us to pull your form back so you can add your information, we can do that. We thank you so much for your time and participation. This might seem like just a simple discussion, but your thoughts, opinions and feedback are extremely valuable to us. We want to ensure that resources are available within our communities so that everyone has access to quality dental and oral care. Thank you.

// end of recording //

Beverly
