# Supplementary material for: A qualitative study of oral health knowledge among African Americans
Source: PLoS One. 2019 Jul 10;14(7):e0219426. doi: 10.1371/journal.pone.0219426 (PMC6619789; doi:10.1371/journal.pone.0219426)
Supplement: S4 Text — This is the Housing focus group transcription. (DOC) [file pone.0219426.s004.doc]

**GEORGETOWN-LOMBARDI HEALTH DISPARITIES INITIATIVE**

**Oral Health Focus Group Transcription– Housing**

**April XX, 2016**

**Project #GGT0421-16**

**ICE BREAKER**

M: We’ll start off by getting to know each other a little bit. Let’s go around the table and have everyone give their pseudo-name and please tell us one thing that you like to do as a hobby.

R: Phyllis (inaudible).

M: Everything that you say is very important to us. We’re going to have to speak up a little louder to make sure we are able to hear.

R: (Inaudible)

M: Thanks, so Phyllis likes to spend time with her kids.

R: (Inaudible)

R: My name is Pearl. I like working with kids (inaudible).

R: My name is Lakisha. I like to go walking.

R: My name is Phyllis and I just like dealing with kids and (inaudible).

R: My name is Patricia and I like dealing with my grandkids and I love to cook.

R: My name is Angie and I’m a security guard.

R: My name is Maria and (inaudible).

M: That’s great. Thank you all for sharing.

**ORAL HEALTH**

M: So, with that, we will get started. What do you think about when you hear the term oral health?

R: Surgery; with me, I say surgery.

R: My name is Pearl and to me, it means surgery, dealing with surgery.

R: Yes, I’m the same.

R: I agree with that. I agree.

M: So Roxanne and Patricia also think about surgery. Anyone else? What other thoughts come to your mind when you hear the term oral health?

R: Angie. Brushing teeth

R: Yes. Yes

R: Yes.

R: Yes.

M: Do you think that oral health problems are as serious as other health problems?

R: Yes.

R: Yes.

R: Yes.

R: Yes.

R: Yes, it’s Pearl.

R: Yes, Patricia.

R: Yes, Angie.

R: Lakisha. Yes.

M: Ok, everyone agrees. Why do you think oral health problems are as important as other health problems?

R: I’m going to go. My name is Pearl. Because everything is going inside your stomach, I mean going into your body parts, all over. You can die from oral health (?) if you don’t really take care of it.

R: I heard that, a disease (inaudible).

M: Ok.

R: Yes, I’m Patricia and I believe that as well. It can cause problems all over.

M: That’s something a lot of people don’t realize.

R: Yes.

M: Many people think that oral health is just about the mouth or teeth. And that’s so not true.

R: Yes.

R: Yes.

R: It’ll hit your ear…cause pain

R: And then that, then when you get that…what do you call that, that stuff that’s in your stomach, that sourness comes through. And then when you belch (?), ooh; I experienced that pain. Whenever I eat, I eat a lot of…I think I do too much eating of a lot of food that has acid, and frying bacon (?). It do not agree with me and I will not learn my lesson. I will not learn about nothing about the greasy food, I guess until they put me in a hospital. But, I experienced something that really got me the other day. I ate a whole chicken (inaudible) and you would not believe the whole thing came right back up. It was the skin, the whole piece. I’m looking at it in the toilet because it was, just that one piece of meat just bloated me and it just…it comes up. When you get bloated, everything comes up. And a lot of that stuff that’s in your stomach, it comes through (inaudible). You’re trying not to let nobody hear when you belch. That’s something. Oh my god, that’s some nasty stuff. Pearl, that’s some nasty stuff. (Laughing)

M: I think everyone agrees with you, Pearl.

R: Yes.

M: So tell me, what is the importance of oral health in your household?

R: Pearl. In my household, well and I got enough toothpaste to last me…because (inaudible) One thing I want to ask about toothbrushes, now, I’m particular about tooth…I don’t like the soft toothbrushes they got now. I do not like them, because I guess growing up with using a regular, hard toothbrush that would always, I would always say that would keep my teeth clean better than them soft. Because the soft ones, like once you put them in, they wear out so fast because…and then, and I’m noticing that I got one of them and it’s wearing out (?), like oh my god, I’ve got to get me, I’ve got to find me a hard…and I can’t find a hard toothbrush, because they say they don’t sell them no more because they damage (?).

M: It’s recommended to use a soft toothbrush to avoid damaging your gums.

R: Pearl. They don’t like to use the hard. They don’t really make it easy, so I said I’ve got to get a good oral toothbrush that I know that won’t wear out as fast as I brush. I don’t want it to wear out.

M: It’s important to have a toothbrush that will clean your teeth properly, but won’t be too harsh. You don’t want one that’s too hard; it could damage your gums.

R: Yes.

R: Yes.

M: So you have to have that balance

R: Yes.

R: Or you can get (inaudible).

M: Yes, they have soft, medium and firm. It’s harder to find the firm ones now, and they are not recommended.

R: Angie. Well, to me, I think the toothbrushes still would…even the soft ones still would damage the gum, because the gums are so tender. And then it’s according, well, how much food you got in between your teeth.

R: Maria. That’s why you floss also.

R: You floss too.

R: Yes.

M: Flossing is very important to clean between teeth where a toothbrush cannot reach. What is the importance of oral health in your household? Aside from brushing and flossing, any other ideas?

R: Maria. Mouthwash.

R: That’s my (inaudible).

R: Patricia. Mouthwash.

M: Ok. What kind of oral care routine do you or your family have?

R: We brush our teeth in the morning, before we eat and probably…and brush our teeth like after we eat something. But obviously when we’re out and about…my son…brushing his teeth or whatever. But yes, we brush in the morning and like after we eat, like when you’re home.

M: That’s good, many people don’t do that.

R: Yes; they just mouthwash and (inaudible).

M: That’s really important.

R: And I saw a lot of people (inaudible).

R: But I say sometimes…I’m Maria, a lot of people sometimes….eating when you’re not at home, so of course you’re not going to brush your teeth when you’re out and about.

R: Yes.

R: But like, you know like me, I know sometimes, which I (inaudible) but you know you carry dental floss. So with dental floss you can still get the food out of your teeth, even when you’re out, so that way you know. And you still get it because you know you go out on the street, you might go to the store to get you a steak and cheese. You’ve got all of these onions on there, so of course you know your breath is going to smell like onions and stuff. So you’re not, you know ain’t nobody going to (inaudible) carry a toothbrush with them, so.

R: Right.

R: Yes.

M: How often do you feel you and your family members should have dental appointments?

R: Pearl. About every six months.

R: Patricia. Yes.

M: So Pearl said every six months and Patricia, every six months.

R: Sasha. I agree.

R: Maria says sometimes every three months.

M: Thanks Maria, why?

R: Maria. Because from the understanding that when I take my grandbaby to the dentist, you’re supposed to…and now the reason why I’m saying three months, because you’re supposed to change your toothbrush at least every three months.

R: Yes.

M: Yes, your toothbrush should be changed every three months. Also, it’s important to note that your dentist may recommend that you come in at different times depending on what your needs are.

R: Yes.

M: Ok. Here’s a big question. Do you have access to a dentist or a dental clinic that is open during hours when you’re free?

R: Sasha. Yes.

R: Maria. Yes.

R: Yes.

R: Roxanne. Yes.

R: Yes.

R: Pearl. I really don’t know because…I don’t know. I can’t really say, because the last clinic I went to I got mad with them people and I ain’t been back since. Because it took them six months before I could be able to get my teeth looked at. And I did not like that, so. And this was at Dental Smiles, down the street and I ain’t been back to the dentist since, but. The way this Medicaid going, I don’t even know what…I’m scared to go in to the dentist. So, I ain’t going to ask that because I had a hassle.

M: So Pearl, when you called, did you have to wait six months to get the first available appointment?

R: Pearl. When it happened, I think what they did with Medicaid this year; I’m going to say it quick, they kind of…with the dental you’re supposed to have your own private dentist. Yes, you do.

R: I go to the clinic (?).

R: Pearl. When I got…I got caught up in that change and they sent me a paper a couple of years ago about my own dentist through Medicaid. When I called to get that appointment, they said that office didn’t exist. So I said I would take a chance, listening to people saying you can go to any dental. But, the more private dentals like Dental Smile and all of them, I wouldn’t go. I mean you could go, but I don’t trust them. Them people that I do not trust, because I went to Smiles down on whatever the street is, down by the (inaudible) and I mean, with that six months. And I’m like, when I went down there, every time and I think what had happened, when they put the paperwork in, some kind of way something there happened. So I called Medicaid. It was there, but Medicaid was telling me, yes, you’ve got to wait for this person. We got to wait. So it took so long. Like I said, six months, and then when I did finally got in, I still had to wait for a month appointment. So I said I’m going to go ahead because my teeth was messed up real bad. And they keep saying I didn’t have no cavities, so I had to get a cleaning.

M: Sometimes teeth can be sensitive or painful.

R: Then they still said I didn’t have no cavities. I got cavities and they’re telling me that I don’t have it. I feel them. So they’re saying that they did and they checked all of the teeth. I went back again to the same people. But still, I said I won’t go to them. So then when I thought about it, I said well, I’ll go to clinic…I didn’t know my Medicaid would let me get, go to the dentist there. So I haven’t thought about trying them. I don’t know. I know they’ll sit up there and tell you, you can’t get this done, you can’t get that done. And it makes me mad, ok, like if I need a filling, send it to Medicaid. It’s going to be so many days before you get it.

M: It’s good that you still get regular check-ups and get a second opinion to be sure.

R: What kind of Medicaid you have?

R: I have straight Medicaid.

R: I have straight Medicaid and I know you can go to (inaudible).

R: Go to who?

R: But see, I’m saying…then they move to D.C. general so that’s where I be doing it, was doing all of my stuff (inaudible). I mean because my clinic is over at Kenilworth. Yes and the dentist over there, that’s why I like them but I don’t want to get up in there, and if I have to have something filled and they tell me I have to come back, make a two-month appointment before Medicaid says so (inaudible).

R: Is that a referral? They got to give you a referral to go to the dentist, you know.

R: No, when you go, well you don’t get the referral; you go and make an appointment. Now, if you need something else done, if you’re just coming there and getting a thorough checkup, they’ll say you need this or you need that. Then they’ll send the paper to Medicaid to get approved for them to do it.

(Inaudible conversation)

R: But then they, but I don’t understand.

R: Six months, so.

R: But I just don’t understand why. Why did they make you wait like that? And they be saying it’s Medicaid. I say why am I going through this?

M: So, what I’m hearing Pearl, is that you’re having a hard time with finding a regular dentist and getting dental appointments within a decent amount of time to get the care that you need?

R: That you can be comfortable with.

M: That you can be comfortable with, accepts your insurance, and who will actually give you an appointment in a decent amount of time? That’s what I’m hearing.

R: Because the last dentist I had was what, a couple of…this was years, like when I was in my twenties. I was going up to northwest. And I had dental work done, right there. I never had to go nowhere else. I had my appointments. I was still going, like you say you go every three months, I was still going. But when I moved and the clinic changed, so it’s under…it’s still the same name, but they got new doctors, so I didn’t go there. So everything, I didn’t go nowhere else. So when I moved over here in Ward 8, I’m thinking I can just go in the clinic to make the appointment. But I be seeing people getting their teeth, getting teeth (inaudible). And I say, good god almighty, Medicaid don’t want me to have this; don’t want me to have that.

M: Now what about everybody else? How easy is it for you to find dentists or dental clinics in your neighborhood?

R: Roxanne. Very good…off of somebody’s Facebook page.

R: Yes, I know about one…maybe I need to go through 1-800-Dentist.

M: Thanks. Let’s be sure to say our names before answering.

R: Maria.

M: So Maria says very easy. Patricia, you also said it’s very easy.

R: Patricia. Very easy.

M: Anyone else?

R: Lakisha, I said it’s very easy too.

R: I think so.

(Inaudible-talking at same time)

R: Because I just took my son to the new clinic up here on…the other day, but he had to have some type of surgery so he can be put to sleep, so they gave me a referral. And his appointment is May 2^nd^ and I think that that was quick.

R: That was quick, yes.

R: Yes.

R: That was good because you get them…see there, for kids, they’ll probably do it better for kids than they would do for adults, maybe.

R: It might be; it might have something to do with…

R: (Inaudible) pulled seven teeth and three…less than two months, Maria had her top grill and (inaudible).

R: See, that’s what I’m saying.

(Inaudible-talking at same time)

R: That’s like a special, like special (inaudible) so they can just do all of them (inaudible).

R: Sasha says they’re good and it’s not hard to find a dentist in my neighborhood. If I go I can make an appointment for my son, most of them, they are family dental centers. So I can go in and make an appointment for my children.

M: Ok.

R: But it’s always again like things done to our mouth, we haven’t had any serious issues I guess of how we go about, how long you actually take…I know it doesn’t take long (inaudible) appointment.

M: How far do you have to go to get to the dentist?

R: Sasha. About thirty minutes.

R: And you can go to the dental clinic by bus, same way.

R: About the same time as mine. Thirty minutes.

M: Ok.

R: Which center?

(Inaudible-talking at same time)

R: Where the Radio Shack used to be.

R: Yes.

R: Oh yes.

M: Now, for those of you who were going to a dentist clinic, how well do you feel that the clinic or the dentist meets your needs?

R: Good.

M: Do you feel that your needs are being met?

R: Roxanne. Yes.

R: Sasha. I love it.

M: Angie does.

R: Sasha does.

R: Maria does.

M: Ok, so you seem to be pretty satisfied?

R: Yes. The only thing Sasha doesn’t like is when we…appointment (inaudible).

R: I mean it’s not the same. Pearl. It’s not just saying that I don’t go, but I’m not particular about…I don’t like to travel through southeast. And then that…Gentle Smiles and all that, I thought they can be used…everybody else can use them. That’s why. And I guess, and I mean I’ve never had a permanent place where I can go straight to a dentist, never did. I just go in certain, into a dental place…

R: And stick with one then.

R: But Pearl, at this point.

R: I’m going to go over to where I go to clinic at.

R: That’s why I don’t mess with them.

R: (Inaudible) losing teeth right here in my mouth.

R: Pearl. But my thing is I know if I go on in, they’re going to tell me something and because they asked me, did I want to pull the teeth out? No, I don’t want you to pull no teeth. If I don’t have nothing to pull, don’t pull them out. But I want them cavities filled, because I’m saying now I got to the point…I’m using a what-you-call-it, dental floss. I’m using them too much so I’m irritating my gums. I use them every day, anytime I’m home, I got me a little thing…so now I just feel like nothing, it’s clean but it just feels like something is coming…you know how between your teeth when it’s clean. And it’s just you do it, constantly every day, every day and that’s when…keeps me going to the dentist, because I don’t, only have…but I know I got some cavities.

M: So Pearl, would it be helpful if you had a list of dentists and dental clinics where you know you can go to receive care?

R: Yes, well I know that’s the problem. Knowing where to go. They keep changing.

R: Maria. So first…with your Medicaid, they didn’t give you a book of providers and dental people?

R: Yes.

R: That’s what I’m saying.

R: Ok, hold on. To make the conversation a little bit shorter, like Maria said, when I found my dentist, I call 1-800-Dentist and they will give you a list of who you can go to. Give them your zip code and they would tell you the nearest dentist to you.

R: Oh, that’s good.

R: What information that you had…what Medicaid, your insurance information and they can tell you all of this and dentist that’s near you. They’ll accept your insurance.

R: They gave me a few I could go to.

M: If you had a dental emergency, do you know of any other places that you could to?

R: Go to the emergency room.

R: Angie. Howard.

M: Thanks, anyone else?

R: Sasha said.You go to the emergency room.

R: The emergency room is not going to do anything.

R: Right, that’s what I’m saying, so you say Howard?

R: Howard Dentist.

R: But I’m saying if your tooth is bothering you, if you have excruciating pain, at 1:00 in the morning you can go to Howard Dentist?

R: No. They’re not open then.

M: So you could go to the emergency room, they would help to treat the pain and if there’s an infection, start an antibiotic.

R: Yes.

R: That’s the only thing the emergency room is going to do.

M: In that urgent situation, they can make sure there isn’t an infection and they can help to take care of the pain. If there is an infection, they can start treating it and prescribe an antibiotic. And then they would most likely refer you to see a dentist for follow-up care of the situation.

R: To a dentist, yes.

R: Oh, yes, thank you.

M: You’re welcome. Do you and your family have dental insurance?

R: Yes.

R: Yes.

M: Thanks Roxanne, let’s make sure we’re saying our names.

R: Make sure I know what ya’ll are talking about.

M: Ok, anyone else? Do you and your family have dental insurance?

R: Yes.

R: Yes.

R: What’s the difference?

R: Yes, for just for me, not anybody else.

M: What’s the difference in whether or not you have dental insurance?

R: Yes, what’s the difference between health and dental?

R: It’s the same.

R: Oh.

R: It’s the same.

R: I was trying…that’s why I was quiet; I got quiet because I was trying to put a question.

M: Well, oftentimes health insurance is separate from dental insurance. For example, health insurance may cover your heart, skin, lungs, etc, but if you need a cavity to be filled that would be covered by dental insurance.

R: Yes, I guess so. I’m not sure.

M: Some health insurance plans will cover everything related to your health…others may cover most things but not dental or vision. For example, under Medicaid coverage however, dental and vision are included.

R: Right.

M: So we specifically ask; do you have dental insurance to make sure that you have coverage for dental care

R: Ok.

R: Wait a minute, excuse me, Pearl. What you mean the dental insurance? I though it’s just regular Medicaid.

M: Yes, Medicaid covers dental, vision and general health. Some people, depending on what insurance they have, may not actually have dental coverage. So it’s important to know what type of insurance we have and what is covered.

R: Pearl. Not all of them. I need to get…if I had a bill I would show it to you. I don’t believe that. I mean I noticed that-Pearl-I notice that everybody, you know (inaudible) and I’m like, I would never think about it, but I’m just noticing how …everybody is getting their teeth done. New glasses, new teeth…whoa! I need to see what I have.

R: Yes.

R: Because my son had to get, what-you-call-it (inaudible) whatever you call it.

M: Oh, an extraction.

R: Yes. And he went to the same dentist and it’s still bothering him…I’ve been trying to ask him about that dentist because we’ve been going to that dentist for some years.

R: The same?

R: Yes and he paid for it out of his pocket, even though he works. And so he had dental insurance, but he pays. He faithfully pays to get his teeth done. And I mean he is…very funny about his teeth. And it was just after he told me about his (inaudible) was giving him headaches. Ok, because I know your teeth are here, so the pain is going up.

M: Yes, and since there are nerves there, it can cause headaches, sinus pain, etc. He should follow-up with a dentist about the pain he’s feeling.

(Inaudible-talking at same time)

R: Every day he would have this painful headache (inaudible) extraction, so whatever. But now he feels so much better, he don’t have it, you know.

M: That’s good.

M: Are you currently working and if so, fulltime or part-time?

R: Pearl, part-time.

R: Angie, part-time.

R: Sasha is part-time.

R: No.

R: No.

M: No?

R: No.

M: For those of you who are currently working, are you receiving any insurance benefits through your employer?

R: No.

R: Sasha says no.

R: Angie. No.

R: Pearl, no.

M: Ok. Is there a policy at work that’s in place that will allow you to take time off for medical or dental appointments?

R: Angie, no.

R: Sasha. Yes, I’ve been lucky.

R: Can you repeat the question?

M: Sure. At your job, anybody have a policy where they’ll let you go in and take off time if you need to go for a medical or dental appointment?

R: Angie. Yes, we can request days off.

R: And before they do the schedule.

R: They don’t want you to, but I told them it’s my health.

R: I know.

R: Sasha. I do all of my stuff in the mornings, so I don’t have to worry about it.

R: You’re working the evenings, aren’t you?

R: Sasha. Yes, so I can do everything in the mornings, so I’m good.

M: How do you feel about the amount of money that you have had to spend out-of-pocket at a dental appointment?

R: Good god. (Sighing)

R: Well, Sasha has a lot of friends that work fulltime that I (inaudible). (Laughing) Get Medicaid through, I mean you know healthcare through their job and they complain a lot. It’s a lot, so I’m kind of blessed to get the free care I can get.

R: Maria. But we are saying it costs you nothing but transportation fare to get there (?).

R: I know that’s right.

R: Angie. (Inaudible) transportation and most health insurance provides transportation, so.

M: That’s a good point too, because you can actually call and schedule a ride through some health insurance plans.

R: Yes.

R: Yes, because and it’s like $2…I think it was $2 for the Medicaid transportation.

R: Metro (inaudible)?

R: No, Medicaid.

R: Huh?

R: Insurance can have transportation. Yes, they got.

R: They got transportation.

R: It’s free.

R: Yes, it’s free. Medicaid transportation is free, because my sister has Medicaid and her transportation is free. Just got to call…with most of the insurance you have to call at least seventy-two hours for your transportation.

M: Do you know what’s covered by your dental insurance?

R: Teeth cleaning.

R: Yes

R: Yes

R: Maria says yes, because most of the time, when you get there, they’ll tell you what’s covered and what’s not covered.

M: Ok. Are you satisfied by your current dental insurance plan? Why and why not?

R: Maria says yes. They cover what I need

R: Sasha says yes.

R: Yes.

R: Angie. I say yes.

R: Phyllis says it’s ok.

M: Phyllis, you don’t sound so sure?

R: Phyllis. I haven’t used it that much.

M: Ok.

R: Patricia says yes.

M: How did you find your dental insurance?

R: Patricia found hers when I drove past (inaudible) and I saw a dental the sign for the dentist (inaudible).

M: Ok.

R: Maria found hers on Facebook.

R: Pearl. What?

R: Maria. My dental plan. I found that on Facebook.

R: Phyllis. She said the dentist is on Facebook?

R: Maria. Yes, but that’s how I found it, on Facebook, and under 1-800-Dentist.

M: Now please describe what your experience has been having your insurance processed by the dentist or dental clinic. Has anyone here had any experiences with that?

R: No.

M: Have there been any problems with the dentist or dental clinic processing your insurance?

R: No.

R: No.

R: No.

R: No.

R: Maria says no.

R: Angie says no.

R: Sasha says no.

M: Has anyone had any issues with providers or dental offices that don’t accept Medicaid?

R: No.

R: Angie says no.

R: Sasha is no.

R: Patricia says no.

R: Pearl. Yes.

M: Ok. What qualities do you look for in a dentist?

R: Angie say the best…quality is the best.

M: Thanks Angie. What is the best?

R: Patricia. They don’t mess up.

R: Sahsa. Good work.

M: Ok.

R: Angie. Look, I got to ask them, what kind of dental (inaudible) master degree. I want to know, before you even work on my mouth, because I don’t want you to be one of them students that you don’t know what you’re doing.

R: Yes.

M: So Angie, you want to know what their qualifications are?

R: Angie. Yes.

M: Angie. Are you qualified to work in my mouth?

R: (Inaudible) yeah, sometimes they send medical students who don’t know what they’re doing.

M: How about anyone else? What do you look for in a dentist?

R: Maria says good work, because when I went to the dentist and they said I had to get seven, possible, teeth pulled. Once they numb you up and then it’s like, boom, boom, boom…says I’m finished. I’m like, oh, well you can pull seven teeth that quick? Bing, bing, bing, when she pulled and all you get (inaudible). So five minutes, seven teeth was out and I was like, wow. And then I got home, I had a whole lot of pain so I had to get the pain pills. It was like, oh, well now I feel pretty good. (Laughing)

R: Oh, for real.

R: This was a female, I mean dentist you got?

R: Maria. A female.

R: Well, I mean where is this located at?

R: Maria. Same place it’s always been, right down there in the shops (inaudible).

M: What other qualities do you look for in a dentist?

R: Good provider (?).

R: Yes, good provider.

M: Good dental work?

R: Patricia. Yes I agree with Maria.

M: How long does it generally take you to schedule an appointment?

R: Pearl. Now that’s going to be a problem. That’s the problem. That’s why I’m not going to (inaudible-talking at same time).

R: Angie. It’s based on you or it’s based on your doctor…a little bit quicker, but you might, got things to do so you might, it might just slip your mind to make an appointment. So you might, you know…but see, the other thing is some dentists, they spend like a phone reminders home saying like you should get your teeth cleaned.

R: Yes.

R: Sahsa. Reminder are good.

R: Yes.

R: Maria says if you call them, they’ll say, well, how soon for the appointment? If I tell them I can come in today often they can see me the same day.

R: I didn’t know they do that.

R: Yes.

(Inaudible-talking at same time)

M: Ok. Do you have a good enough relationship with your dentist or dental health provider that you feel you can trust the decisions or advice that they give you?

R: Maria says yes.

R: Sasha. Yes.

R: Roxanne says yes.

M: So Phyllis is yes?

R: Phyllis. Yes.

M: Ok. Do you feel that it’s easy or difficult to talk to your dentist about your or your family members’ care?

R: Roxanne says yes, it’s easy.

R: I say yes, Patricia.

M: So is it easy or difficult?

R: Patricia. Easy.

R: Sasha. It’s easy.

R: It’s easy.

R: Maria says yes.

R: Maria. It’s easy.

R: Sasha. When you build a relationship with them, sometimes if I go in, they can squeeze me in if I have a question.

R: Yes.

R: Sasha. And you know so you could talk to them about, you know, certain things.

R: Angie. Right, yes.

R: Just outside the dental relationship.

M: Ok. After each visit, do you feel that you know better how to care for your teeth and gums?

R: Yes.

R: Sasha says yes.

R: Patricia says yes.

R: Angie says yes.

R: Maria says yes.

R: Same thing.

R: Danielle (?) says yes.

M: What has your dentist ever told you about things that you can do to have good oral health?

R: Brush twice a day.

R: Yes, brush.

R: Brush, floss, mouthwash.

R: Eat right.

R: Yes.

R: Yes, eat right.

R: Maria says eat right.

R: And if you get them (inaudible) make sure you take care of those.

M: When you’ve been to the dentist, how do you feel that the dentist treats you?

R: Good.

R: Roxanne says good.

R: Angie says nice.

R: Sasha says with respect.

M: And that’s important.

R: Yes.

M: Very important.

R: Patricia says good.

M: Ok. Has your dentist ever talked to you about pulling versus saving teeth?

R: Yes.

R: Angie says yes.

R: Sasha says no.

M: Ok. Now, for those whose dentists have talked to them about pulling versus saving teeth, what has the dentist told you?

R: Well, Angie, they have told me all mine was bad and they need to pull them. They couldn’t save none of them.

R: Yes.

R: Maria, they told me all of mine were broke off so they were pieces of teeth, so they had to pull them.

M: Anyone else?

R: Yes, I have bad teeth and couldn’t save them so they pulled mine too. Patricia.

**HEALTH COMMUNICATION**

M: Are you currently doing any kind of preventative maintenance for dental care?

R: Aside from getting your teeth cleaned?

M: Yes, aside from brushing, and flossing on your own. What else are you doing in terms of preventative or maintenance care?

R: Chew gum.

M: Ok, what kind of gum?

R: Like Orbit or something like that.

M: Ok, the sugar-free gum?

R: Yes.

M: Ok. Has your dentist ever talked to you about diseases such as cancers, HPV, cardiovascular disease, or diabetes?

R: Sasha says no. And you said your dentist, right?

M: Yes. Your dentist.

R: No. My dentist never told me anything about those.

R: No.

R: Maria says yes.

R: Patricia says no.

R: Angie says no.

M: All right, so we have mostly no for that question, but Maria, you said yes?

R: Maria. Yes.

M: So which of these has your dentist talked to you about?

R: Maria. Oral cancer.

M: Ok, thanks Maria. Have you ever been screened for oral cancer?

R: No. Maria says no.

M: Anyone else? Have you been screened for oral cancer?

R: Roxanne says no.

R: No.

R: Sasha says no.

M: Ok. How about HPV?

R: No.

R: No.

R: Angie says no.

R: No.

R: Not at the dentist; that’s crazy. That’s a good question.

M: Have you ever missed work before because of mouth pain?

R: Sasha says no.

R: Roxanne says no.

R: Angie says yes.

R: Pearl, no.

R: Maria says yes, when I was working.

R: Yes.

M: Have you ever gone to the emergency room because of mouth pain?

R: Sasha says no.

R: Maria says yes.

R: Pearl says no.

R: Patricia says no.

R: Roxanne says yes.

M: Ok. Have you ever tried to treat yourself for mouth or tooth pain?

R: Yes.

R: Patricia, yes.

M: What did you do?

R: I used Motrin, take Motrin.

M: How about you, Maria?

R: Maria has used lemon extract to help with pain in teeth.

M: I haven’t heard of that before.

R: Angie used Baby Orajel.

R: Yes. Me too.

M: Ok.

R: Salt and water with me; Pearl, salt and water.

M: Ok, Salt and water.

R: Oh yes. Me too.

M: For all who have treated yourself for mouth or oral pain, why didn’t you go to a dental health provider?

R: Can you repeat that?

M: Sure, for those who have treated themselves for mouth or oral pain, why didn’t you go to a dental health provider, the dentist or dental clinic?

R: Angie, I thought I could stop it myself.

R: Maria. It was afterhours and I couldn’t get to the dentist.

R: Same thing.

M: For those who have lost teeth, do you feel that it has affected your chewing or digestion?

R: Angie says yes.

R: Pearl. Yes.

R: Maria says yes.

M: Do you feel that losing teeth is a normal part of getting older?

R: No, I don’t think. Pearl.

R: Maria says no.

R: No.

R: Angie says no.

M: Do you feel that losing teeth is a normal part of getting older?

R: Patricia says no.

R: Sasha says she doesn’t; but it seems like everyone that’s older than me always have fake teeth (?). I don’t know.

M: Do you know of any risky behaviors that can contribute to poor oral health?

R: Sasha. No, but I know…you know being pregnant can cause it.

R: Maria says not taking care of your teeth.

R: Angie. Yes. I agree with Maria.

M: Now, what about smoking?

R: Oh, yes.

M: Do you think it can contribute?

R: Yes.

R: Angie says yes.

R: Yes.

R: Maria says yes.

R: Pearl says yes.

M: Everyone’s saying yes.

R: Patricia says yes.

R: I was going to say coffee too.

M: Coffee, ok.

R: Caffeine.

M: Now, what about drinking alcoholic beverages. Do you think this could possibly contribute to poor oral health?

R: Oh, I’m not sure.

R: No.

R: Yes, that might.

R: You never know.

M: How about sexual activity?

R: With oral, yes.

R: Oh yes.

R: Yes.

M: A lot of people don’t think about that.

R: Yes.

R: Maria just wants to say I’m saying yes, because I was looking on Facebook yesterday and they had a picture of a man’s private area, but he must have oral sex with somebody and it had all of these bumps (?) on it…oral sex with somebody and (inaudible).

M: And that’s something a lot of people don’t realize.

R: Yes.

M: That sexual activity can affect your oral health. It can open you up to infections, including STDs like the human papillomavirus, also known as HPV.

R: Yes.

M: And HPV can lead to oral cancer.

R: Yes.

M: Do you feel that it’s important to eat a healthy diet in order to have good oral health?

R: Yes.

R: Yes.

R: Patricia, yes.

R: Pearl, yes.

M: Ok. We touched on this topic a little bit earlier, but what kinds of foods do you think may increase your risk of having poor oral health?

R: Pearl. Sugar.

R: Roxanne said sugar.

R: Your teeth, not enough calcium.

R: I would say maybe caffeine.

M: Caffeine?

R: Soda (?).

R: Oh yes.

M: Yes, it often contains a high amount of sugar and acid

R: Yes.

M: Also, sugar-sweetened beverages like juices. That can do it too. And it’s not so much the juice itself. It doesn’t mean you can’t have juice. But it’s not good to have the sugar and acid from juice sitting on teeth. Over time can wear down your enamel.

R: Yes.

M: You also could end up with cavities and even tooth loss, over long periods of time. So it’s really important.

R: That’s why Maria said you have to brush your teeth like say at nighttime, so you won’t go to sleep with all of that sugar on your teeth.

M: Right. And even with children, a lot of people think, oh, well children, they’re going to lose their first set of teeth anyway.

R: Yes.

M: But it’s important to make sure that their teeth are clean too.

R: Oh no, I know kids that have teeth lot’s of cavities. Two to four years old.

M: And the thing is, the way that they start out with their oral healthcare helps to guide how their oral care will be in the future.

R: Yes.

M: So it’s important to start them off the right way. We often don’t realize that oral health problems can actually cause death. A few years ago there was a young boy named Deamonte Driver who had an untreated dental infection that formed an abscess. It went to his brain and by the time he received medical care, it was too late. He died.

R: That’s why I say all of this affects you. I believe it.

M: There’s also a fourteen-month-old girl who, last week in Texas, she was at the dentist getting cavities filled, and she died due to an undetermined complication.

R: Fourteen-months-old, come on.

(Inaudible-talking at same time)

R: The juice, like sugar.

R: The nipples on the bottle.

R: Yes, that does it too.

R: It can make their teeth grow out crooked and all of that…pacifier stuff.

R: Oh.

R: Damn.

R: Wow.

M: It’s something that many people don’t think about. Oral health problems can cause great harm.

R: Right.

M: And it can affect your cardiovascular health.

R: Yes.

M: It can affect your brain health.

R: Your heart, all of that.

M: It’s all connected. We have already talked a bit about foods that may contribute to poor oral health. What foods and beverages do you think are good for your oral health?

R: Water.

R: Yes. Water.

M: Yes, that’s one of the most important ones.

R: Drink a lot of water.

R: Yes.

R: Because that keeps the gums healthy.

R: And Angie says milk.

M: Yes, milk is very good.

R: Milk.

R: Pearl, what about if you got lactose, so milk is not good, what do you drink?

R: They got lactose.

R: They got regular lactose milk.

M: Yes, you can get milk without lactose if you are lactose-intolerant.

R: Pearl. Because of my stomach.

R: It’s not as sweet; it’s good to me.

R: Pearl. I don’t know; I just can’t drink it. It really don’t agree with me because of (inaudible).

R: You try a different type (inaudible).

(Inaudible-talking at same time)

R: Soy.

R: A lot of people like almond, but I was on soy milk.

R: I mean I would rather prefer to have (inaudible) and I know that can mess my gums up, but I just cannot eat a soft apple. I will not eat a soft apple.

M: And apples are good for your oral. There is that saying, “An apple a day keeps the doctor away.” Of course, after eating you’ll want to brush the teeth or at least rinse out your mouth so the food, acid, and sugar are not sitting on teeth.

R: Yes.

R: Pearl. But I have to eliminate the milk. I done tried all of it. I don’t even like the 2%. I just won’t. My stomach will not agree. I thought the lactose was going to do it and I kept telling my doctor. I say don’t do nothing but just…I just don’t have good…my stomach just can’t take it.

M: That’s why it’s important to know yourself. You know what works for you. If it doesn’t agree with you, then you know to avoid it. Everyone is different.

M: Do you have regular dental checkups?

R: Yes.

R: Yes.

R: Well Sasha and her son gets regular checkups.

R: I’m going to say I haven’t had a regular checkup. Pearl.

R: I haven’t but I need to go.

R: Well, Maria says she’s too busy worrying about other people’s issues and not her own, so that’s why I haven’t.

R: I have regular checkups.

R: I need to go.

R: And Angie don’t have regular checkups.

M: You don’t?

R: No.

M: Are you afraid to go to the dentist?

R: No.

R: Sasha says no.

R: Angie’s going to say…

R: Pearl. I have five teeth.

R: I have six teeth.

R: What’s the name of it?

R: Four wisdom and two.

R: They messed up my mouth.

R: I guess they’re my jaw teeth (?).

(Inaudible-talking at same time)

R: No, you have to get it out.

R: I don’t know what they said was wrong, but they keep, every time I go to the dentist…if it’s not paining me…it never bothers me. But I know it needs to be pulled.

M: Has fear or stress ever prevented you from going to the dentist?

R: No.

R: Patricia, no.

R: Yes, Pearl.

M: Ok.

R: Fear.

M: Roxanne says no.

R: I was.

M: What was that?

R: I was but I’m still a little bit kind of fear.

R: Maria says she just scared of needles, but when I got to go, I don’t like the pain, I go.

M: For those who have children, how easy is it for you to find childcare when you need to go to the dentist for yourself?

R: Sasha says it’s easy, because her child is in school.

M: Ok.

R: 7:00 to 6:00.

R: But Maria says easy because all of her children are grown.

R: Mine are grown too.

R: Angie says the same thing that Maria said, all grown. They got their own.

R: Yes.

M: Ok. Have you ever received information about oral health or preventative care that was connected with other issues, like oral hygiene, smoking cessation or dietary counseling?

R: Patricia, yes.

R: Yes.

R: Roxanne says no.

R: Angie, smoking.

R: Maria says yes.

R: So yes, I agree with the smoking.

R: Yes.

R: Because we did for many years.

R: That the dentist told you?

R: No, we had (inaudible) last year, I mean this year. They came and did the dental work.

R: Passing out all of the…

R: Yes, we had somebody from (inaudible) came and do the dental work for the kids. Some of the kids got dental work and then they (inaudible) the little teeth, showing how to brush, toothbrush.

R: Oh yes.

R: How to open it up and show them how you go to the back. And then they had them doing it too…toothbrush, had a little mirror where they can see how the teeth look when they brush them. So it ain’t like this (inaudible) don’t have, but we do have somebody come do that.

R: Like resources.

R: Yes.

M: Now, how do you like to receive information about health?

R: About?

M: About health. For example, do you like to receive it like on the television, the news, email?

R: Either way, Pearl, email, television or mail.

R: Roxanne, internet?

R: Yes.

R: Facebook?

R: Out on the street.

R: Everything.

R: TV, out on the streets.

R: Everything.

R: You can receive information any way.

R: I say everything.

R: Maria says brochures, when people come out and pass out brochures.

R: Flyers.

M: What about text messages?

R: Yes.

R: Yes.

R: Well, if they could send something to…Pearl, they can send something to us; we can pass it on too.

R: To the next.

R: To the next person, yes.

M: About how much time do you spend each day with these different kinds of communication, with the TV, text messaging, internet?

R: Always on the internet.

R: Every day.

R: I’m always on TV.

R: All of that.

R: Pearl, TV, it’d be all of them, but mostly I’m on TV.

M: Ok, out of all of these different modes of communication, which do you pay the most attention to?

R: TV.

M: TV?

R: Yes.

R: Yes, Roxanne, TV.

R: Facebook.

R: Facebook and TV.

R: Yes.

R: Pearl, TV.

M: And out of all of these, which do you feel will be the best way to reach you with information about oral health?

R: Maria, email.

R: Roxanne, email.

R: Phyllis, email and telephone.

R: TV.

M: In your daily media use, do you remember seeing or hearing anything about oral health?

R: Yes.

R: What did you say?

M: In your daily media use, do you remember seeing or hearing anything about oral health?

R: Yes.

R: I think about oral health on TV.

R: Yes.

R: They say…so much.

M: What messages have you heard?

R: The different advertisement; then you got some…Pearl, you got some dentists that are private and they are advertised, their services.

R: Facebook; might be on Facebook now.

R: I wouldn’t be surprised. I would not be surprised. Because it just seems like everything.

R: Pops up on Facebook, yes, everything.

R: And especially if you’re a new person, you’ll see stuff. I didn’t put that up there.

R: Maria says it’s (inaudible) sometimes people share stuff, so that way, the other person can see. Like, Maria might know about one doctor. And I can share that with some people; say oh, that’s what happens to you (inaudible).

M: Anyone else? Please describe the message that you’ve seen or heard that has encouraged you to maintain good oral health. Any messages that pop into your head that you remember that encouraged you to maintain good oral health?

R: Maria says when you see a picture of a person with cancer or damage to them.

R: Lord have mercy.

R: Person not taking care of it.

M: And they have a lot of those.

R: Yes.

M: Anyone else?

R: They should be talking about how rank (?) your teeth be (inaudible).

R: And also Maria says with, if you’re not taking care of your oral health, you know they’ll tell you about not taking care of oral health causes you to have odor.

R: Yes.

R: They call that, what they call that? What is it?

R: Gingivitis?

M: Halitosis?

R: Yes.

R: Yes.

R: Yes.

M: Would you be interested in receiving information about oral health and preventative care from us?

R: Yes.

R: Maria says yes.

R: Yes.

R: Pearl says yes.

R: Anything, yes.

R: Pearl says yes.

M: Well, we’ve come to the end of our discussion. We thank you so much for spending the time with us. We know that you didn’t have to come and we appreciate everything that you had to say. We don’t want to just kind of keep it here, just take notes. We actually want to develop programs and put additional resources in our community. So we thank you for your contribution. And as a token of our appreciation for your time and your contribution to the discussion, we want to give you a $25 gift card.

R: Oh, thank you. We didn’t know that.

R: Thank you.

M: Now, you do have a choice. Either Safeway or CVS, so think about it and I’m going to come around with the list.

R: Maria says Safeway.

R: Safeway.

R: Pearl says Safeway.

R: Yes.

R: We’re all saying Safeway.

// end of recording //

Nocona
