# Supplementary material for: A qualitative study of oral health knowledge among African Americans
Source: PLoS One. 2019 Jul 10;14(7):e0219426. doi: 10.1371/journal.pone.0219426 (PMC6619789; doi:10.1371/journal.pone.0219426)
Supplement: S5 Text — This is the Senior Recreation Center focus group transcription. (DOC) [file pone.0219426.s005.doc]

GEORGETOWN-LOMBARDI HEALTH DISPARITIES INITIATIVE

Oral Health Focus Group Transcription– Senior Recreation Center

March XX, 2016

Project GGT0421-16

**ICE BREAKER**

M: Hi again everyone. Thank you for coming to speak with us today. Okay, is everyone ready? To get started, we’re going to go around the table. Everyone please say your pseudo name and mention one thing that you like to do as a hobby.

R: My name is Karen, and as a hobby I like bingo and like crib. I do crib once a week.

M: Okay, thank you, Karen.

R: I’m Liz. I like bingo, I like being around the seniors, and I like having fun.

M: Thank you, Liz. Maria?

R: I’m Maria. I like bingo and I like to do decorations inside the house. I like changing things.

M: Thank you, Maria. Tracy?

R: My name is Tracy. I like to go to bingo, I like to go to church – out of the heat into the shade, and the tea tray stuff.

M: Thank you. We’ll go to Anita.

R: My name is Louise…

R: No. Anita.

R: Alright. My name is Anita.

M: And please name one thing that you like to do as a hobby.

R: I like to play bingo.

M: Thank you. Okay, Sarah?

R: My name is Sarah, and I like to play cards, I like to play bingo, and I like to play dominoes.

M: We’ll go back to Adrianne.

R: My name is Adrianne and I like to make jewelry and I like to be with my grandkids.

M: Thank you. And then Michelle?

R: Michelle. I like being around the seniors and also I like being around my grandkids and I like playing bingo and cards. I don’t live in this building, but I come here every day, and I enjoy coming here and being around the seniors.

M: Thank you.

R: My name is Sandra. I like playing computer games.

M: Okay. Helen?

R: My name is Helen, and I enjoy being around the seniors and around my grandchildren, and I like doing the puzzles they give us in our building. And I like to play cards and I love music. So when I’m alone, I listen to my music.

M: Thank you, Ellen. Donna?

R: Yeah. My name is Donna and I love to do exercise, play cards, and also I love my friends. I participate with them and we do a lot of things together. So I just love to do a lot of stuff with everybody.

M: Great.

R: My name is Tiffany. I love to sing, I love to travel, and I love being around the seniors and playing bingo.

M: We have a lot of talent in this room! Thanks. We’ll start with the first question. Before each answer, please say your pseudo-name. For example, I’m Sarah, in my experience ….

**ORAL HEALTH**

**Knowledge**

M: What comes to mind when you hear the term “oral health”? What do you think of? Adrianne?

R: My name is Adrianne. My teeth come to mind and the dentist.

M: Thank you. Anyone else? We can all just kind of jump in.

R: My name is Leeanna and I think about my body all over the whole…

M: That’s a good one. Thank you. Anyone else?

R: My name is Liz. I like going to the doctor because I’m concerned about my health, at 63 years old.

M: And we all should be. Thank you, Liz. Anyone else? Okay. Do you think that oral health problems are as serious as other health problems?

R: Adrianne. I take it very seriously because if you don’t get your teeth and stuff taken care of – I know a lot of people who passed away by having bad teeth in their mouth, and they passed away behind that, by not getting proper dentists or not going back and forth to the dentist.

M: Does anyone have anything to add?

R: My name is Liz. Because you can get gangrene (?) with your teeth. You’ve got to keep your hygiene up, your teeth up, and with the gum and to the point you won’t have no teeth.

M: Does everyone think that oral health problems are as serious as other health problems?

Rs: Yes. (multiple)

R: It’s going into your body, yes.

M: And that’s something a lot of people don’t realize. Oral health sometimes seems to be separate, but it’s really not.

R: Yes.

M: Do you know of any connections between oral health and there rest of your body? I know Liz mentioned some. Does anyone else know of any connections between oral health and other health problems in your body?

R: Well, what about diabetic?

M: Yes, diabetes can affect oral health.

R: And also kidney problems, and all of that. I go to George Washington for at least four doctors. Eyes. That’s another problem. I was over there yesterday. My kidney, and diabetic, my eyes. I’ve been having problems. I take 10 pills a day, 4 insulin a day, and I have to take one in the morning, one at lunchtime and two before bedtime. And you know, I get so tired, but this is my health and if I don’t do it nobody’s going to do it for me.

M: Yes.

R: Um hmm. That’s true.

R: Like I got a problem, when I first got rheumatoid arthritis, they showed me a film, showing how to inject myself three times a day. It was nervous. I reached a point, I went to stand up, I didn’t do it proper, I start bleeding, I get frustrated and crying, and do the other side all over. The doctor said if you can’t do it, nobody else is going to do it for you. So the Lord took the fear out of me and I did it for five years. To a point it stopped and I had to get the ambulance to my house because the (unintelligible) wasn’t working anymore.

M: Okay.

R: Then they put me on something else. That worked for four years. Then they put me on something this year that lasted nine months and now that’s not working. So now I’m leaving it in the Lord’s hands.

M: And it’s good that you keep going to maintain your health.

R: I’ve been going to GW over 20 years and I’m 63.

R: Yeah, a lot of people give up.

M: A lot of people do give up, but it’s important not to.

R: I’m scared of needles.

M: And the thing is, if you don’t go, then you’re not able to get the help that you need. And we all need help. So it’s important to keep going, get your checkups, follow up with your general medical, and also dental. It’s very important. If at any time you feel you need a second opinion from another doctor, you can do that too.

R: But you know, since I first developed diabetes, they put you in the hospital, and you would have therapy with orange peels, where they taught you how to stick it and inject into the oranges, and you stayed with it until you learned how to stick that orange.

M: So Karen brings up an interesting point. With diabetes, you mentioned being taught in the hospital how to do the needle stick– Now, in terms of oral care, do you feel that when you go to the dentist or dental clinic you come away knowing how to better care for your teeth, your gums?

R: Yes (multiple).

R: They told me to floss three times a day.

M: Okay.

R: Keep your appointments up, keep your teeth together so you won’t get pyuria. And that’s what I been doing. They told me to change my toothbrush every three months.

M: That’s very important.

R: Put a cap on it so it won’t get bacteria. You have to change your toothbrush, use it for three months and then throw it away. A lot of people don’t know that. A lot of people use the toothbrush a whole year. It’s bacteria. First you wash it off with hot water, then you start your procedures going in your mouth.

R: I fell about 10 years ago, and I knocked everything out. I had to have 22 stitches in my gum, and all of this, they’re implants. They’re not plates, they’re implants. But when I was going through that, the doctor worked with me about different things that you have to do, so I actually had schooling on what I had to do to take care of my mouth. And I don’t have any problems. A couple of weeks ago – I go every three months for cleaning and examination – and when I went to see them last month, it seemed like when I would eat or drink something cold, it would kind of ache, so what they did was I had to go back and he had something that he put on the tooth, and that took care of it, so I don’t have that problem anymore.

M: And so you actually have been going in for checkups and cleanings.

R: Every three months, for about the last five or six years.

M: Ok, so how about everyone else? What is your oral care routine?

R: I have to go in every six months. I had a root canal. Everybody’s scared about the root canal – this is the last talk. Talking about the root canal, it made me nervous. I got there, I was calm. He said he was finished. I just thought he was just starting. And he was finished.

M: Wow.

R: Yeah, they don’t want that root canal.

R: That was five years ago.

M: That’s good. Maria, did you want to add something?

R: I go every three months.

M: Okay, every three months. That’s good. Adrianne?

R: Yes, my name is Adrianne, and I go to the dentist. I get fillings and I get checked also. But my twin passed away from cancer, and I believe that she had, like Miss Karen had all hers knocked out. My twin had all hers taken out, and put more in, like Miss Karen. Implants, right. And I believe that the implants poisoned her system, because my twin passed away and she had got that done and she wasn’t well after that at all, from that time. But back in the day, they were just learning how to do that, but now, when Miss Karen got hers done, they…

R: It’s been a good 10 or 15 years since I got this done.

R: Right. My twin got hers a long time ago. We’re 62 now, so it was when she was in her 40s.

M: Okay.

R: And I believe that my twin passed away from her teeth poisoning her system. Because I’ve been checked and checked, and I get checked for cancer and I don’t have what my twin had. So I believe that that infected my twin and made my twin pass away through her gums.

M: Oral health is very serious. My deepest condolences for your loss.

R: Thank you so much.

M: Tracy?

R: I went to the dentist, and they take my teeth out. (unintelligible) So they had to take my back teeth out of my mouth and gave me false teeth. He had to hold my hand and when they took it out I thought it would hurt. I went home that day, my mouth was sore, and they gave me some pills. And I thank god they gave me some teeth, I could take it out and put it back in. I wait for five years to get another.

M: It’s really important to know if there’s a problem with your teeth. Some people figure, well, I have bad teeth, there’s nothing I can do. But in truth, there is a lot that can be done. So it’s important to get check-ups and preventative care.

R: My name is Tiffany. I had a bad tooth up here, and it caused my ear to act up. Like I had an ear infection.

M: Yes, it can cause pain and problems in other places.

R: But now I need to get to the dentist right now.

R: Yeah, you could lose your hearing behind that.

M: You can. It can go to your brain, too. So it’s important. There was a young boy named Deamonte Driver who unfortunately lost his life due to an untreated tooth abscess. The infection spread to his brain and he died.

**Accessibility/Availability**

M: Do you have access to a dentist or a dental clinic during the hours when you’re free?

Rs: Yes.

M: Okay. Where do you generally go for dental health services?

R: I go to 3333 14^th^ St, Liz. I’ve been going there for 20 years, and I continue to keep going. I’m very pleased with them. And they take Medicaid and Medicare.

M: Is that a dental clinic or is it a private dentist’s office?

R: It’s a private dentist, but they take Medicaid and Medicare.

M: Okay, that’s good to know.

R: And I’m Karen, and I go to 2141 K Street to a private dentist. And I’ve been going to him over 20 years.

M: Wow.

R: Adrianne. I go to Dr. Lee, and he’s on Pennsylvania Avenue, and I’ve been going there almost 30 years. He’s with my insurance for my job. I retired, and I still can go there. Sunshine is on my policy, so both of us are still going to him. She can go until she’s 26 years old.

M: Sunshine is your daughter?

R: Yes.

M: And you can go to the same dentist?

R: We’ve been going to the same dentist ever since – for me, ever since I’ve been 30 years, and ever since Sunshine has been born, we’ve been going to Dr. Lee, Pennsylvania Avenue.

M: Is Dr. Lee’s office a private dentist’s office?

R: Private. Yeah, he’s right there by the Library of Congress, across the street.

M: Okay. Anyone else? Thank you.

R: I am Sarah, and I go to visit my dentist every six months. My private doctor is located in Columbia Road and 17^th^ Street. I know it’s very important to visit the doctor because I lost my root canal in my teeth from when I had the pain for almost 18 years. I lost last year when I was getting the lunch, I felt my tooth come out, and now I am doing the implant. And it’s so important to have all the teeth because when you try to do the implant it costs a lot of money. And it’s almost more than $3000. Only for one. And I have to pay from my pocket because the Medicare, they don’t cover it. So that’s why I know it’s very important because – right now I could eat better because the doctor already started trimming last month since January and I have to wait another six months to finish all the trimming. But it’s getting so expensive. And the people they don’t care. They care, which I do, is clean inside, with your teeth.

M: Yes, it can be very expensive. Problems can build up over time. Even small problems.

R: I’m Karen, and when I went to the dentist the last time, he gave me a medical toothpaste. You still use your regular toothpaste, but then you brush at night with this toothpaste. It’s mentholated, and I don’t know what it’s supposed to do, but I had called him this morning to call in a prescription for it. It’s something that you have to get with a prescription. And I’ve never gotten it before. They always gave it to me, so I don’t know what the cost is. They’ve got to call it in to the pharmacist.

M: I hope it doesn’t cost much. I’ll by praying for you. Liz?

R: Yeah. If you have good insurance, you better use it because it will be taken away from you. You better use it. Everybody can’t get what we have. They’d love to have the insurance we have, so you better use it.

M: Yeah. Many people have dental insurance and don’t use it.

R: This is Liz talking. They don’t care.

M: Tracy, you were saying something?

R: When you call them, they don’t take Medicaid A and B. My momma couldn’t get her teeth (unintelligible). Me and my sister and my brother – we had to get also teeth from our mouth. We had to pay for it. She don’t wear it no more.

M: And that’s because they didn’t accept Medicaid?

R: Um hmm.

R: I’ve got Medicaid and Medicare.

M: Okay, so you can use either.

R: Yeah, I’m in the bracket.

M: Okay.

R: My name is Ellen. I was going to the Unity Clinic down the street, 1850 Delaware Avenue, for years, since 1949, when they opened. And I went there to the dental clinic and I had a tooth that had the silver filling, you know they used to put in your tooth when you had a cavity? Well, that had turned dark, and I wanted it taken out, so the dental surgeon said no, they didn’t want to take it out because it would impair my health when he bring the tooth out. So I went to DC General, and they pulled the tooth, because it had gotten an abscess on it. So they pulled that tooth. I was glad of that. I didn’t listen to that one down at the clinic. They pulled it out. I haven’t had any problems so far.

M: Ellen, it’s good that you went for a second opinion. The preference is to save teeth if possible, but sometimes that may not be possible.

R: Well that’s what I got, a second opinion.

R: My name is Donna, and we have a dentist coming over to our building. So he seemed to be pretty good, so he’s making me some dentures, so we’ll see how that goes.

M: Good. So the dentist actually comes to your building?

R: Yes. They just started across the street.

M: Adrianne has a question.

R: Miss Adrianne is asking Miss Donna a question. Does anybody else come there?

R: Yeah, you come over and talk to Miss Linda.

R: Okay.

R: And advise her of that, and she’ll tell you exactly what to do.

R: Okay.

M: What’s your dentist’s name?

R: I can’t remember.

R: It’s called Community or Environment – where they come out and help the seniors. I was wondering, what do they do for people who are not in your buildings? This service that they give, they give quite a few services in the building, because I’ve been on there when they got them. But I was wondering what’s going to happen – the clinic is going to close. And that is the only thing I know that’s close to free service, because if you don’t have money they still take you. So I don’t know what is going to happen.

M: That’s something that we want to work on, too, is making sure that resources are in place in the community. And that whatever resources are here, that we know about it and the community knows about it, too.

R: Well see, the ones that’s coming to her building are not around here. They’re outside. They come to the building and introduce themselves. Like if I had space, I would give more of it, too. Which I use here, as space. And a health lady came once, I brought her in here. There are different ones.

M: That’s good to know.

R: Yeah, they go to your house.

R: Ellen. I go to the same dental technician that she’s talking about. I went in there and they were supposed to make me some dentures, and because I didn’t have Medicaid, I had Medicare, they said no, they couldn’t do it. But they said now, we can do it, we can make your teeth, but you have to pay for them. And that’s why I haven’t been back.

R: See, that’s the difference between this outside service coming in. So that they even had a beauty parlor come over there.

M: Really?

R: Oh yeah.

R: She did nails and then they had a long tractor-trailer, because if they wouldn’t accept them in the building, so they had to do it on the van. You just walk in and they could do everything.

R: Beautician, yeah.

M: There are dental vans, too, that go around to the community sometimes.

Rs: Yeah.

M: How easy is it for you to find dentists or dental clinics in your neighborhood?

R: It’s not easy.

R; It ain’t easy.

R: This is Miss Liz talking. They send you a form in your mailbox. It’s a booklet that Medicaid gives you, you look up and you can find your own dentist. It’s like a pamphlet, but it’s like a book. And you could find dentists. Whatever you want, they give you options.

M: So it makes it easy for you to find.

R: I mean I haven’t had that problem, but I get it every year at my mail slot because I have Medicare and Medicaid. So they say I might want to switch. So they give you the thing in your mail slot once a year. Even for your feet – anything, even for your eyes – anything having to do with your body. It’s very easy, because you get this thing in your mail slot once a year.

M: How about everyone else?

(multiple voices)

R: I don’t get what Liz get. If I did get it, I don’t have the insurance yet. I have Medicare A and B, but I don’t have Medicaid. They’ve not giving me nothing.

M: So for Adrianne it’s not easy.

R: Adrienne. No, it’s not easy.

R: Sandra. I see on TV, and I have seen it more than once, and it has Medicaid (unintelligible) 10. And it says sign up and check and see how far your Medicaid can go. A lot of people ignore it. Like me, I ignored it, too.

R: My name is Ellen. Like I told you, when I went to the dentist and they told me if I didn’t have the dentist in our building, if I didn’t have Medicaid they couldn’t make the teeth for me. She said, well, why don’t you go there and get the straight-out Medicaid? But I have QMB. That comes on the Medicaid. And they pay my premium of my check I get every month, so I’m not going down there worrying about getting straight Medicaid, you know. So I just have to find another dentist and see what they charge.

R: This is Liz talking again. We have a senior citizen banking (?) thing you do once a month. And they gave us a third insurance, and these are the two that I have right now. My third one is coming to my mail slot next week. They helped us last Friday – there were like 300 seniors there, and they help you with a third insurance, because a lot of people don’t have this.

(multiple voices)

R: I almost had it but I’m going to talk about Mrs. Anita.

M: Okay.

R: Because she doesn’t want to talk, but Mrs. Anita is having problems. She took out all her teeth last year. She was doing the trimming at the Howard University dental clinic. But all the time she go they sent the students to work for Mrs. Anita. So they never finished the work. And then I went to – she says she had, Mrs. Anita had another dentist at the Howard University, Dr. King. But he was working when she was younger for Mrs. Anita. Later they sent her to the dental school because the students can finish the work. And then every time they send a different student. And now they don’t finish the job. And then they told me, I have to look for the dentist in the Medicaid card. She also has Medicaid. Now I don’t know what happened, but now the dentist, they don’t want to continue working no more for Mrs. Anita.

R: I know why.

R: It’s so hard for me. I want to help Miss Anita, to help her teeth, because she is worrying too much. She can’t eat very good. Sometimes she gets so nervous because she has appetite to eat, but she can’t chew it.

M: So she needs to find another dentist.

R: Yes, somebody can pay insurance, please. I don’t know if you could be helping me.

R: You know the lady that was in here the last time? The lady that I brought in here? That’s what she was here for. Does she have both cards, Blue Shield, Blue Cross?

R: No. She had the Bravo Insurance, and they say they don’t take it.

R: Bravo’s no good.

R: Yeah.

R: Let me tell you what happens. A lot of times they go in these senior buildings, and these seniors don’t have a good understanding of what they’re signing up for. And they sign up for things that they shouldn’t. They should keep what they got. Now she’s got to switch back in order to get service.

R: She didn’t do anything. The court lawyer is in charge for Mrs. Anita. And she may order the (unintelligible). I don’t know what the program, but…

R: She’d got to switch back in order to get that service. (multiple)

R: See, some insurance, they run short-term, long-term, and some of the insurance don’t even do surgery.

R: She has already one surgery. And the doctor said she doesn’t want to do no more. She sent me to another doctor, and the doctor say before they was taking the insurance, but later they said they don’t want to work no more with that insurance.

M: It can be frustrating when there’s a lot of back and forth with insurance and dentists.

R: And we try to get them help, but like Miss Walker, she approached me. She said she lost her health aide because of that. It’s how you go about these things.

R: That’s sad.

M: Maria?

R: My name is Maria. Now because Miss Walker lost her aide because she had to get a form filled out from her doctor, and because the aide’s got to get paid, because they did me the same way. But now, I called, they called CBS said I should not never cut off my insurance. I should not cut off, because they jeopardize my life – my medicine is important. I have a new kidney transplant in me that I could lose that if I don’t take my medicine.

M: That’s serious.

R: That is serious.

R: Karen. One time, two years ago, I was paying so much money, co-payments for going to the doctor, medicine. Medicine was costing anywhere from $30 to $50 for a prescription. And what my daughter did, she called down to the Medicare legal department and made an appointment for us to speak to a lawyer, and we went there and she and I explained to the man that was assigned to us the problem that I was having, that I was on a fixed income, and the medicine and the medicine and co-pays and things was so high. And he in turn connected me with a different insurance. I had Bravo, so he cut me off and I said take me off of that, and he put me on a different insurance, and after that when everything was squared away, I go to the doctor now and unless it’s a specialist, I pay no co-pay. I go to the drugstore to get my prescription and pay $6 or something like that, from $35 or $45.

R: What kind of insurance do you have now?

R: I can’t think of the name of it.

R: But you had to switch in order to…

R: Yeah.

M: So you were on Bravo and when you switched to this other one, now…

R: Yeah. I’ve still got the red, white, and blue card.

R: My name is Ellen. I was on Bravo, too, and they waited until I was getting ready to have surgery and the people called me and told me I couldn’t have the surgery because Bravo wasn’t going to pay for it. So I’ve switched now to Cigna. But isn’t that something?

R: I am Sarah. Yesterday I had another surprise. I called to the New Hampshire Pharmacy for the supplies, because her primary doctor sent the paperwork to get the supplies. And when I called yesterday to see if they received the paperwork by fax, they said they received the paperwork but the problem is Bravo, they don’t pay for any supplies. Nothing. I can’t do nothing, because she has the lawyer, and she doesn’t get the care.

R: Fire the lawyer.

R: Legal aid. Nobody can do nothing because Miss Anita can do nothing. She forgot about everything.

(multiple voices)

M: At the end of the session, if you don’t mind I want to take your information and see if we can help.

R: Please, I would be very glad give it.

M: We’ll see if we can point you in the right direction with that. And that brings me to a question.

R: I really appreciate it.

R: We all need help.

M: Yes, we do, every single one of us. I want to know for all of us who are here, do you have dental insurance?

Rs: No. Yes (multiple).

R: No, I don’t.

R: I don’t have it, either.

M: And Ellen doesn’t.

R: This is Liz again, and I’m 63, I still have all my teeth in my mouth.

M: That’s great Liz.

R: I’m going to keep my mouth forever, my mouth is my beauty. Along with my hair.

(laughter)

M: How does the dentist or the dental clinic where you go meet your needs? Do you feel that your needs are being met?

R: Yes, 20 years.

R: Liz. Very pleased

M: Karen, yes, 100% your needs are met. Adrianne is pleased. Everyone else?

R: Maria is pleased.

M: Ellen, no.

R: No.

M: Ok, and for Sarah and Anita, the answer is no. Do you know of any places that you could go to if you had a dental emergency other than the dentist’s office?

R: Yes. I could go the hospital.

R: I go to GW for emergencies.

R: Yeah, I go to Howard University.

M: So Ellen goes to Howard.

R: And they have also cleaning, too.

M: Okay, so Sarah, you go to the Cardoza Clinic if you have a dental problem.

R: Yes.

M: Okay, anyone else?

R: Yeah, I can go there also. I go there, too. Cardoza, because that’s the Unity Clinic up there, and they have one down the street. So I could go to either one.

R: I really don’t because he’s a private doctor. I guess I would have to go to a hospital or somewhere if I had a problem. Because it’s closed in the evening.

M: So the hours of operation.

R: Yeah, just to let her know, Unity have a walk-in clinic on Minnesota Avenue. One up off of Cardoza. They both work Saturday and Sunday. So if she had an emergency…

R: You have to have the insurance.

R: I just have the Medicare and the QMB, that’s the only ones I have, and I’ve been going there for years.

M: Okay.

R: My name is Tracy. (unintelligible) they don’t do that no more.

R: Well, you mean down the street? What clinic are you talking about?

R: Cardoza?

R: Yeah. They don’t do that no more.

R: Adrianne. I need a second one, so that’s why I want to get information from Miss Liz. She’s on Columbia Avenue.

R: No Liz, that’s you, Dianne.

R: Oh, okay.

R: Put your address here, then it’s 3333 14^th^ Street. I’ll call you, get your phone number. I showed you the building two years ago.

**Insurance and Affordability**

M: How do you feel about the amount of money that you end up having to spend in out of pocket at a dentist’s office?

R: Miss Liz talking again. I don’t have to pay.

M: Okay.

R: My name is Tiffany. I was told I had to pay, but before I had AmeriHealth Insurance, but now I’ve got SSI because of a certain situation, so they changed mine to straight Medicaid. So I’m not sure yet if I have to pay for certain stuff. I have to find all of that information out. I don’t know yet.

M: Okay.

R: No. This is Miss Liz talking. You need to get in touch with your health insurance. You need to get the book in your mail slot and it will guide you from there. Call on the back of your card.

R: But I have a dentist already that I go to. I don’t need the book, really.

(multiple voices)

R: Oh.

R: Yeah, you need what I got, yes. You need the book.

R: But I do have a dentist, 1613 Harvard Street.

R: I’ve been there, too.

M: That’s a private dentist’s office?

R: Private dentist, yes. I’ll probably just stay there where I’m at. If they don’t charge me too much for my services for the straight Medicaid, I just might finish it up there.

M: As long as it meets your needs.

R: Miss Liz talking. You can go somewhere where you don’t have to pay nothing.

R: Oh, just Medicaid?

R: Use that money for something else, like getting your house.

R: Oh, yeah, okay.

R: Yes, I’m going to talk to you about that.

M: And that brings me to another question. Do you know what’s covered by your insurance?

R: A lot of people don’t know. Because they don’t get the pamphlet in the mail slot.

M: So Liz knows. A lot of people don’t know. Do you know what’s covered?

R: Yes. So Liz knows.

R: If I don’t know, my daughter takes care of all that, and she finds out what’s what, and I don’t never need to worry about anything as far as medical or whatever. She oversee it all, she acts more like a mother.

(multiple voices)

R: Well, they just changed my insurance without them telling me. I didn’t know they was going to change it, because of the situation that I’m going to be doing something different now, so they didn’t even send me no book or stuff in the mail, so I’ve probably just got to get on the phone and call them. I don’t know, because they just changed my Medicaid and when I went to pick up my prescription from CVS when they called me, I found out I was paying out of pocket and I wasn’t paying before with the other insurance.

R: I had to make all the calls because they didn’t tell me about the straight Medicaid, but I did get on the phone and I got the card right away.

M: Oh. Yes, you definitely need to find out.

R: Them that have straight Medicaid have no problem. If they have a problem, there’s a number that you call.

R: On the back of your card?

R: You could even get a scooter.

R: No, I don’t need no scooter. I don’t want no walker s and no scooter.

R: Blue Shield, it will interfere with Medicaid every time. It look like they take more money from Medicare than they do from Medicaid.

M: So what I’m hearing, is that it matters what kind of dental health insurance you have. And knowing what they actually cover. Because you can have an insurance that doesn’t really cover your needs. So it’s important to know what kind of insurance coverage you have and what they actually cover. For those of you who are working, is there a policy in place at your job for taking time off to go to dental appointments?

R: We don’t work.

M: Ok.

R: I am the only one still working? Sad.

(laughter)

M: Only Sarah.

R: Tiffany works, but I can be there when I want to be there. Flexibility. I’m on my own time.

M: For those of you who have health insurance, please describe what your experience has been with having your insurance processed by the dental clinic.

R: No problems.

M: So Susan has had no problems with insurance.

R: Liz has no problems. The owner does my mouth.

M: Wow, okay.

R: The owner.

R: The owner does mine also. He tells me that I have a lot of money to get my teeth fixed that’s in the pot. He said, you’ve got all this money. He said, you have money that’s set aside to get my teeth fixed, so whatever I need to be done, I have money in the pot already that’s on my insurance. And I didn’t understand what they were saying – from my job – until my doctor explained it to me. I don’t want too much done after my twin passed, because I don’t want to have no taking out and put all my teeth back in, because my twin told me don’t do it, because that’s what killed her, she said.

R: No problems with mine whatsoever.

M: The thing is, it’s hard to know exactly what happened, or what caused that, but it’s important to definitely for dental check-ups, and make sure that you have the care that you need for yourself.

R: Yes.

M: So I also want to check… Liz, you had a comment?

R: Yeah. If you have any rotten teeth in your mouth, you better get rid of them, because we want to see you again next year.

R: Amen.

R: Yeah, because you get drainage from a tooth down in your body. And it affects a whole lot.

M: And it can spread through your body. It’s important to have that taken care of.

R: It can mess you hearing up, and your eyes.

R: Yes (multiple).

M: So it’s important to have dental check-ups and screenings. Make sure your dental health, or oral health overall is intact.

R: I don’t like the dentist.

R: I love my dentist.

(multiple voices)

R: My gums are so very sensitive. So what they have to do, they have to swab me first, gel me, and then stick it over my tongue, because he said my teeth is like this. So close together. I’ve got very sensitive gums.

R: Me, too.

R: I brush my teeth and they do not bleed, but it’s very sensitive, so they do me two ways. Even when I get my teeth cleaned, they have to gel me, and then stick me.

M: It’s good that your dentist takes the time to make sure that you’re comfortable during treatments.

R: Yes. But if he’s not, I’m going to speak up for myself. I don’t what that in mouth – where that needle at? I don’t see that needle. I need that needle.

**Dental Appointments**

M: So that brings me to my next question. What qualities do you look for in a dentist? Adrianne?

R: Adrianne. That the place is neat and clean, well taken care of, and they wash their hands and stuff like that. And brand new equipment, and not wash it and use the same thing in my mouth and in her mouth.

R: Adrianne. They pull it off the little plastic thing, and then they lay it on a little tray. And then they cover it up.

R: Right. That’s how mine does it.

M: So you want to make sure that they’re clean.

R: Right. If it’s going in my mouth, I’m looking.

R: Yeah, me, too.

M: Does anyone else have anything to add? What qualities do you look for in a dentist, Sarah?

R: Yes, when I go to see the dentist, I like see how many years has experience. Make sure, because experience is very important. Because sometimes they open the clinic, you don’t know how many years has the experience, maybe it’s just graduate. I don’t have problem with doctors who just graduate, but it’s very important in somebody you could trust who has more experience. Reference is very important, too. The reference and the experience of other people.

M: Good point

R: Do you remember a couple years ago there were a lot of dentists were down because their equipment wasn’t up to par?

R: As a matter of fact, it was last year, and another year, and they showed them on TV, and some of these people didn’t have licenses to go up in your mouth. All this was affecting Maryland, not DC, though.

M: And they weren’t supposed to be.

R: Right. They shut them down. It was on the news.

M: Ok, so it’s important, too, to know who you’re going to. Check to be sure the person is qualified and make sure it’s someone who has training in whatever field you need treatment from.

R: I think everybody knows how a license is supposed to look.

R: And their references are very important.

M: Their references, yeah. If you know people who have gone to that dentist and give good reviews of them, then you can kind of believe a bit more that that person knows what they’re doing because they’re all good references.

R: Um hmm.

M: Now in terms of scheduling dental appointments, how long does it usually take for you to get a dental appointment that you need?

R: If I called today, they could get me next month.

M: Okay

R: Karen. Mine is automatically scheduled unless it’s an emergency. So yeah, I go every three months for x-rays and cleaning and all. But if I called and say such and such is wrong, in a few days I would have an appointment.

M: That’s good. Adrianne?

R: He would tell me to come in right now.

M: So you can get an appointment right away?

R: I could go right now. He’d say come on in.

R: Tiffany. I could get one right away or I could just walk in there on my own.

R: I’ve got to call first.

R: Yeah, I’d have to call and make the appointment.

R: That’s why I call on the phone. I can’t walk in.

R: Yeah.

M: How about you, Sarah?

R: I am Sarah, and I used to call in and they find out when they have available, so they give me the date to see the doctor. The give me the appointment. Sometimes they are one week, two weeks, or one month.

M: Okay. Do you have a good enough relationship with your dentist or dental health provider that you feel you can trust what they say when they advise you about what needs to be done with your dental work?

R: Karen. Yeah, it’s been 20 years.

M: Ok, so you trust your dental provider.

(multiple voices)

M: You also trust your dental provider. Adrianne?

R: Yes, I do. I’ve been there for 30 years.

R: I trust mine, too.

M: Donna, do you trust your dental provider?

R: Donna. I don’t know. I’m just getting used to this one.

M: Thanks. Ellen?

R: Ellen. I just started going.

M: Just started as well. Michelle?

R: Michelle. I just started. I haven’t really been back yet because the only thing I need is the lower part. And I haven’t been there yet. But you know what, I really feel that if your teeth can go, you can use your hearing. Oh yeah. I believe that’s what happened to me. I lost 80% of my hearing, and I never got a hearing aid because that’s another thing that’s expensive. A hearing aid. I’ve been needing one between 8 and 10 years. But I never got it.

M: Have you tried to go through insurance to get the hearing aid?

R: Yep, I have been to the insurance. But I’m going to try again this year when I go to my primary doctor. I’m going to talk to her about it.

M: Yes, try again.

R: Yeah. Like I say, on my right side all the way down is what I have a problem with. My hearing, the tooth, my hip.

R: Did you have a mild stroke?

R: And my arm. All that on the right side. And also my eye problem. My eye have something to do with it, too.

M: You should definitely follow up with your doctor. And if you feel you need to, get another opinion from another doctor. But definitely follow up to make sure that you’re doing well.

R: Yeah.

M: After each visit with your dentist or dental health provider, do you feel that you know better how to care for your teeth and gums? Liz?

R: I know more now than I knew back then, when we was in school. I know more now.

M: Okay, so you definitely do. Karen?

R: Usually after every appointment it seem to me like I learned something that I didn’t know before.

M: That’s very good. Adrianne?

R: Yes, I have, too. My name is Adrianne, and the last time I went to the dentist, he gave me this thing that you take and you put it down this way – it’s like how long you’re supposed to brush your teeth. And I’ve never seen that. When it all goes down in there like salt, that’s when you’re finished brushing your teeth. And I’ve never had that. And I’m 60 some years old. Then you put it this way and that’s how long you’re supposed to brush your teeth. And I gave one to all my grandkids. I have one, and like Miss Liz said, you’re supposed to throw your toothbrush away every three months. Have them covered up and just like what Miss Karen said, you’re supposed to do those things. And I didn’t know them. And you learn as you go. And the more you go to the doctor, the more you learn more things. You learn how to take care of your teeth and your grandkids’ teeth.

R: The dentist is still – when he’s working on you, he’s talking and telling you different things the whole time that are true.

M: I think I want to go to your dentist!

R: You go to Dr. Lee.

R: I’m Tiffany. Dr. Sherman is so quick. I sat there and I think less than – he had to do an extraction of my wisdom tooth, and I sat down. It was just a few little minutes. I just sat down. He said something to me – and I don’t remember what he was talking about, but he said I’m done. And I jumped up. I had a smile on my face when I left.

M: So he was done and you were happy with his work.

R: I’m always happy – I don’t know, but I’ve seen people in there saying things, they look like – I guess it’s how they take pain, but they was coming out all like this. Some was coming out with pain medicine. I wasn’t even in pain. The lady at the desk was saying, “You still happy, and you got a smile on your face.” I said, that’s because it’s a good dentist.

R: You got good gums. Your gums mean a lot.

R: I’ve always been okay with the gum thing. My gums are very sensitive, because I went to this other little place and the lady told me, like, where did I go? I was at another organization, and she said you can get $10 if you go – for people that had, I think, straight Medicaid, got $20. And she took us to this place to get our teeth done with a dentist, and I just went to get them cleaned, but since I only had straight Medicaid, they paid me $10 instead of the $20. But the man that was cleaning my teeth, I didn’t really like how he was cleaning my teeth. It hurt. I had to fake him out like I was really having more pains to make him hurry up and finish my mouth. And he was like, I didn’t even do anything. I said I will never go back here. I just wanted to get my $10. I said I will never be back there again. And they wanted me to come back, the lady was like, when are you going to do the appointment? I said I’ll just give you a call later. I’m not going back to that place.

M: That brings up another point, too. You may have had a bad experience with a particular dentist, but it doesn’t mean you will have a bad experience with all dentists. So it’s important try another one. Don’t stop because you had a bad experience with one or two. Find another one.

R: Like you said, they give up.

M: And it’s important not to give up, because guess what, we have to be our own advocate. We have to take care of ourselves, so if a provider isn’t meeting your needs, you can find another one that is a good fit for you.

R: Like you said, a lot of people be scared. They don’t want to go back.

M: Do you feel that you’re able to talk with your dental health provider?

Rs: Yes.

M: So Tiffany is comfortable, Liz is comfortable, Adrianne is comfortable, anyone else? Ellen is comfortable. Okay. That’s good.

R: I am enjoying this, I like this.

M: I’m happy you are. What has your dentist ever told you about things you can do to have good oral health?

R: Liz. Dental floss.

R: Sarah. Rinse the mouth with Listerine.

R: Brush twice a day.

R: Miss Adrianne talking. He told me to continue to floss, all the time, and those things I was not flossing. He told me I need to do both.

R: Me, too.

R: And you know, he told me about the Waterpic, too. And they also tell you about using it. I love that Waterpic. It’s better than doing this.

R: Yeah.

R: I am Sarah. It’s also good to take care of rinse the teeth with salt water, too. Sometimes I don’t like the (unintelligible) from Listerine, so I like to do with the clear water. It’s much easier warm water and put the salt in there and rinse my mouth.

M: Okay, so instead of Listerine or mouthwash sometimes you like to use salt water.

R: Yes.

R: I don’t like the dental floss or the little pick thing. Sometimes I just get my toothbrush and just brush my teeth again. And I even have a travel toothbrush, too, I carry in my purse.

(multiple voices)

R: Or a toothpick. You have to put a string around it. And they have mint. It’s a mint one. Safeway has them. You can get 90 of them or 30 for $2 or $3. Sometimes you can catch I on sale for $1.

R: I use them.

M: Oh Adrianne has one now.

R: I have one right here. I keep them to get the food out of my mouth.

M: It’s important, whether it’s by Waterpic, regular dental floss or those picks with floss on them, to get between teeth, because the toothbrush is not able to clean between teeth properly.

(multiple voices)

R: Miss Adrianne. My doctor told me not to use a regular toothpick. He told me a regular toothpick, it goes all in my mouth. He told me to use those flossing sticks. He told me don’t use them. It breaks in your teeth.

Rs: Yeah.

R: And I don’t even buy them anymore. I buy these now. And these got taste to them. They got a minty taste, and they come in…

R: I just brush my teeth again. I normally brush them more than twice.

R: I do, too, but I can’t get the food out.

R: My toothbrush do. I get all the way…

R: Huh uh, no you can’t.

R: I can get all the way down there.

R: You can’t do that. Don’t do that. Don’t tell no story now.

R: Mine do.

M: So let me ask you – when you’ve been to the dentist, how do you feel that the dentist treats you?

R: He treat me good.

R: Yes, Miss Adrianne. My dentist treats me very nice, and he is Japanese. And he’s very, very gentle and kind and he talks – like Miss Liz say, he makes me comfortable, because I don’t like the dentist, and this is the reason why I haven’t switched to another dentist, because he’s real quiet and he makes me comfortable and he doesn’t make me nervous. Like with Miss Tiffany, like she said, she went and her doctor made her nervous.

R: No, he don’t make me nervous.

R: You said he was digging real hard, and…

R: Oh, that, oh.

R: My doctor, if I raise my hand, that means he’s hurting me. That’s what he told me to do. He’s gentle. He talks to me the whole time. He lets me know. I’m getting ready to give the needle, I’m getting ready to do this. So this is the reason why me and Sunshine have went nowhere else. Because he’s gentle and he talks to me. He tells me everything he’s doing before he do it.

M: That’s good. How about everyone else? How does your dentist treat you?

R: Mine is just like hers.

M: Liz and Tiffany have a similar experience as what Adrianne has.

R: Very gentle and understanding. He tells you everything.

M: That’s very good, very good. How about anyone else? Sarah.

R: Yeah. I have the same experience, very good experience for the dentist, because she is a dental Arabic (?). But she speaks English, Spanish. So to make me feel comfortable she speaks some type of Spanish.

R: Oh, that’s nice.

R: Yeah, that’s nice, and she is very sweet, very kind, very nice doctor. She made me feel comfortable.

M: That’s wonderful.

R: I’m Karen. Mine, he almost acts like we’re friends.

(laughter)

M: That’s very good, so far I’m hearing that everybody pretty much had a good experience and you feel that your dentist treats you well?

Rs: Yes.

M: How about you, Maria? Are you happy with how your dentist treats you?

R: Yes.

M: Michelle, are you happy with how your dentist treats you?

R: Michelle. Yes.

M: Okay. Does your dentist talk to you about pulling teeth versus saving teeth?

Rs: Yes.

R: I have – what do you call it? Your back tooth, your wisdom tooth. He’s surprised my wisdom tooth haven’t been giving me any problem when I’m 63. I’ve still got 32 teeth in my mouth. He says your wisdom tooth is good. Does it ache? Nope.

M: That’s really good.

R: Will you have to pull it? If it’s not bothering you, then no I don’t.

M: How about everyone else? Has your dentist talked to you about pulling teeth versus saving teeth?

R: I am Sarah. I don’t know if he is normal. My doctor said when I had the other dentist, they told me I have to pull it out, the ones in the back, because that’s not good – it doesn’t work anything in my mouth. They say sometimes it’s much easier to catch the cavities with the other teeth. So he told me to pull out the four. One on the top, one on the bottom and the other side, too.

M: Those might be your wisdom teeth.

R: Yeah, and then they say the rest is okay, but this doesn’t – it’s not important. Keep it.

R: That’s what he told me, too. The wisdom tooth, they’re not that important to keep.

M: So Karen, your dentist told you the same thing?

R: Karen. Yes. Not this one I go to, it was years and years ago, when I was going to other dentists, they said that. And Dr. Rose, he did not pull them.

R: Excuse me, Mrs. Anita wants to go to the bathroom.

M: Oh, that’s fine, sure. No need to ask.

**Oral Health History**

R: And the dentist told me sweets will ruin your teeth. And that’s probably why I still got all my teeth in my mouth right now. I eat sweets, but I eat them in spells. If I buy some cookies, next month they’re going to be in the trash, because they’re stale. I don’t deal with sweets too much. That’s why he told me I’ve still got all my teeth in my mouth. I don’t do candy or sweets. Give me some chocolate ice cream.

R: I eat sweets every now and then – I’m Tiffany – but when I’m finished… but I don’t eat it that much. It’s only certain things I like. I eat them in spells and I’ll brush my teeth.

M: And that’s very important. Tiffany just hit right on it. You have to get that sugar off your teeth. Adrianne?

R: Yes. This is Miss Adrianne. The key is like Miss Tiffany said, that’s what I tell my grandkids. You can eat all the candy you want, as long as you brush your teeth after.

R: I brushes mine, and I’m good.

R: You still can’t do that.

(multiple voices)

M: Let’s hear Susan.

R: Do you know the contents of dark sodas?

R: I’ve learned the hard way. They do mess up – they eat the enamel off your teeth.

R: That’s right.

M: Good point, soda can wear away at the enamel. The thing, too is, not just dark sodas, but pretty much all sodas have a lot of acid.

Rs: Acid.

R: It sure is. You got to be cool about that the soda drink, too.

(multiple voices)

M: Has your dentist ever talked to you about diseases such as cancer, HPV, cardiovascular disease, or diabetes?

Rs: Yes.

M: Adrianne, yes. Karen, no.

R: Because my twin had cancer and she passed away, so I’m being checked regularly for dentist and at the doctor’s.

M: Anyone else?

R: I get an x-ray and cleaning every three months, so that’s what they’re doing there.

M: Okay. Liz?

R: When you go to the dentist, it’s not sitting down and getting your teeth cleaned. The first thing they do is x-rays. Then we find out what they’re going to do with you. X-ray first. That means a lot.

M: Susan?

R: You know, we left out one little, teensy thing. A lot of people like talking about their teeth, but do they brush them? No they don’t.

M: Brushing is very important.

R: Didn’t Miss Liz say that? Change your toothbrush when? Every three months.

R: Yes, Adrianne. I try to brush mine before I go to bed and it’s so hard. Like Miss Susan said, it’s so hard, and sometimes I get up, when I go to the bathroom, I brush my teeth if I didn’t do it before I get to bed and I’ve got my grandkids doing it. As a matter of fact, the dentist told them to brush before they go to bed.

R: It’s three times a day.

R: Right. And they do it before they – well they can’t do it while they’re in school, they’re not going to let them do it, but they should. But they’re not going to let them do it. But they do it at night, and I’m doing it at night, and just like Miss Susan said, I forget at night.

R: We all do.

(multiple voices)

R: I’m doing it during the day. And I’m struggling, like Miss Susan says, I’m struggling at night and when I get up and go to the bathroom, I end up doing it.

R: These people, when they got yellow teeth all in their mouth – they got yellow teeth plus rotten teeth. They need to brush their teeth.

M: It’s important to note that just because teeth are yellow doesn’t necessarily mean that they’re dirty. Sometimes teeth are stained by the things that we eat or drink, like wine and coffee. Nicotine can also stain teeth.

R: And smoking will put your teeth in bad shape.

(multiple voices)

R: My teeth are pure white.

M: Have you ever been screened for oral cancers or HPV at your dentist’s office or clinic?

R: Yeah. Can you explain HPV?

M: Sure. HPV is the human papilloma virus. It’s a sexually transmitted disease.

R: Yes, and that’s the shot that they wanted to give – they give it to the girls now.

M: Yes, there are HPV vaccines to help prevent infection with the virus.

R: And it’s like a mandatory – they’re making me do it, but I have custody of a couple of girls now. They’re not my kids, but my girlfriend’s daughter died from that shot.

R: Oh, my god.

M: From the shot?

R: Yes, the shot. And so I didn’t get the shot. I let their mother, their mother has to be able to say to give them that shot. They almost kicked the girls out of school that I had because they didn’t get that shot.

R: Are you serious?

R: Yeah, they’re making all the girls get it now. Right?

R: Oh my goodness.

M: There is a mandate for them to be vaccinated, but if children and/or parents don’t want to, they can opt out. I don’t know of any deaths caused by the vaccine. There can be side effects however from the vaccination, such as pain and swelling at the site of injection, fever, nausea, and lightheadedness. Your arm may also be sore for a couple of days, just like with the flu shot.

(multiple voices)

M: Not just gums, but also your sexual organs.

R: Right, there you go. They give it for the sexual organs, but they’re only giving it to the girls, right?

R: They should be giving it to the boys.

M: No, it’s not just for girls, it’s there’s actually a mandate for boys in DC now as well.

R: They need it. Because they’re the ones that need it.

R: You know they need it.

M: It goes both ways. Both males and females need it to help protect themselves against HPV.

R: Yes. well, my girlfriends daughter died from it.

R: She didn’t sue?

R: I don’t know anything about what she’s doing. I just know the girls that I have custody for that I need to sign for, so I made their mother sign. I didn’t do it. I don’t know what that, like my girlfriend’s girlfriend said.

R: You said the woman died.

R: Her daughter died from that shot.

M: I’m very sorry for her loss. Unfortunately, none of us here know what the reason may have been for her passing. To date, there have been no deaths found to be caused by the Gaurdasil vaccine. However, we all have the right to look into it and choose for ourselves whether or not to have the vaccine.

R: After she got that shot, she passed away.

R: I know this. It makes you bleed heavy. A lot of females don’t like to take it because it causes headaches – it has a couple of side effects.

R: Yeah, a lot of side effects.

(multiple voices)

M: You’re talking about the HPV vaccine?

Rs: Yes. It has side effects.

R: Liver and kidneys.

M: People who have liver or kidney problems should speak with their doctor before vaccination and before starting any new medications. However, I have not heard of any cases in which liver or kidney problems were caused by the HPV vaccine.

R: No. When they came out, Rhonda had several stories about that. And about heavy bleeding.

M: About heavy bleeding and HPV?

R: And headaches.

R: Yes, different little things. A lot of side effects, a lot.

R: Yeah, I heard that there had been girls that they reported lots of side effects.

R: Not specifically about the heavy bleeding, but they were reporting some side effects. Like getting dizzy, and there was one death, with the girl who had died, they didn’t know that she had lupus. So she did pass because they doctor that administered the shot didn’t know that she young lady had lupus.

R: Oh, I believed you.

M: Well, that’s important to know. too, Lupus is an autoimmune condition, and autoimmune conditions fight against your own body, much less something foreign that’s coming in. So it’s important for your doctor to know your entire health situation before you make a decision like that. We are all different. Having an autoimmune condition can definitely have a big impact on how your body responds. So talk with your doctor first.

Rs: Yes, right.

R: I think that’s what it was– the mother that actually signed it didn’t give it any thought.

R: Oh, okay.

M: It’s important for the doctors to know so they can help you to make an informed decision.

R: Yeah, he supposedly looked it up before he gave her it. That’s why I didn’t sign for those girls, their mother did. I didn’t want that on my head. I’m not signing for that shot. Their mother had to do it.

R: And in case you get a new doctor, to tell your doctor your medical history. And they pull a copy of your record from the previous doctor to send to your new doctor.

R: Years ago I didn’t pay attention to directions with medicines and things, but now any new medicine that a doctor give me, that sheet of paper that’s in there, I read that from A to Z before I take it, and if there’s something on there that I don’t think I ought to take it, I won’t take it at all.

M: That’s very important, too. Read thoroughly anything that you’re given to sign before you have any kind of treatment. Make sure you know exactly what’s what before you agree to it. And also make sure your doctor, whether a regular medical doctor or a dentist, knows what your health condition is. They’ll ask you your medical history. Make sure you let them know exactly what is going on.

R: I did. I read it. They put a form out and they kicked them out from school, and then they had to put them back in school because I told them, I’m not signing it, and they’re going back to school. Both of them.

M: It’s a choice. You can choose.

R: But they put them out of school.

R: That’s messed up.

R: Yeah, they put pressure and put them out of school. Kicked them out and told them I had to sign for that shot.

M: You don’t have to sign. You can opt out. If you don’t want them to have it, you’re their guardian, you can opt out.

R: That’s just like inoculations for small pox, chicken pox. That’s mandatory.

M: You always have a choice. In some situations you can request an exemption.

R: But they always ask you, do you have a religious…

M: They do.

R: Yeah, they ask you that.

R: They want to know what your religion is.

R: And those kids was kicked out.

R: That’s sad.

M: Ok, let’s get back to oral health. Have you ever missed work before because of mouth pain?

R: No.

R: Yes.

M: So Adrianne, yes. Karen?

R: I did when I fell and knocked everything out, and I missed about a week’s work. I was on my way to work when I feel. I was running to catch the bus to go to work, and I tripped over a tree branch and that’s when I went face down. And I was out of work for about two weeks because the ambulance took me straight to Howard. That’s where they gave me the 22 stitches up there that night.

M: Wow. Has anyone else ever missed work because of mouth pain?

R: I am Sarah. When I pull out the molar, I used to continue bleeding all the time. Sometimes the next day, too. So I can’t go to work like that with the bleeding, even I put in the gauze every time. But I don’t know what happened. So that’s why I’m scared to pull out one more tooth from my mouth, because that’s what starts the bleeding.

R: Did you get stitches?

R: No, they didn’t give me, because they took it out, one, and they say it’s not necessary to give a stitch, but if you need, they do. But this time was better, when they are going to start with the implant. It was much better. I don’t know what they did, but this new doctor worked much better.

R: That’s good.

R: They stopped the bleeding faster, in 30 minutes. And I went back home and I had no more bleeding. They told me I don’t need to put anything and leave like that.

M: Have you ever had to go to the emergency room because of mouth pain?

Rs: No.

M: So Karen, yes. Adrianne?

R: Yes.

R: Tiffany. I wanted to say I stopped it on my own so I didn’t have to go to the emergency room, because I had fresh piece of garlic, and I just stuck it up on the side of my gum and the it knocked out the pain right away.

M: Okay. So that brings me to the next thing. Have any of you ever treated yourself for mouth pain instead of going to a dental care provider?

R: I never had that problem.

M: So Maria, yes. How, how did you treat yourself?

R: I put that – the purple kind, and they had a little thing on there. And put it in your mouth – you just put it on your teeth.

M: It takes the pain away – Ambesol.

R: Ambesol, or gelatin garlic.

R: I use fresh garlic.

R: I use a tea bag.

M: Oh, Adrianne uses tea bags, okay.

R: Peroxide also helps.

M: With the pain?

R: Yeah.

R: And I use Bayer aspirin.

M: Bayer aspirin, okay.

R: Not to swallow it, but put it on my tooth.

M: Oh, you put it directly on there, okay. For those of you who have treated yourself for mouth pain, why didn’t you seek help from a dental care provider?

R: A lot of times you can’t get there when you can.

R: Yeah.

M: Susan says sometimes you can’t get to a dental provider.

R: That’s true, yeah.

R: You do it temporarily and then you just make your appointment.

M: Okay.

R: I didn’t have to go to an emergency room.

M: Sometimes it’s just a temporary thing, is what I’m hearing. And then other times you’re just not able to get to a dental care provider at the time when you’re having pain.

R: I can use something nobody has ever tried, and it actually works.

R: What?

M: What is that?

R: It’s vinegar, pure vinegar. And it’ll knock that pain out.

R: It’s white vinegar.

R: It’ll stop your pain.

R: Oh, you put something on there and held the vinegar on it?

R: You can just put it in your mouth. And it’ll knock it right out.

M: Have you or any of your family members ever lost any teeth?

Rs: Yeah.

R: You know when something fall out in there. On their own.

R: Tiffany. I maybe had a bad tooth, because I bit a cheeseburger and it came out. And it wasn’t bleeding. It didn’t even hurt.

M: It just came right out?

R: I had no pain.

M: For those of you who have lost teeth, has it affected chewing or digestion?

R: Someone who has missing teeth…

(multiple voices)

M: But while they were out…

R: Yeah, I had a whole lot of them fall out.

R: I’ve got to some here.

M: Does it affect chewing or digestion?

R: I just move the food.

M: Do you believe that losing teeth is a normal part of getting older?

Rs: No.

M: So Liz says no, Karen says no. I’m hearing no’s all around the room.

R: My father was in his 80s when he passed and he had every one of his.

R: My mother is 80… So I guess it’s that chewing tobacco that kept it back in there.

M: Well, you’ve got to be careful of that, though, because tobacco can lead to oral cancer.

R: Yes it can, yes.

R: Tiffany. When you take care of them, a friend of mine back in high school, she was so young when she lost all of her teeth in high school. She just used to eat bags of candy. Not brushing her teeth, not taking care until they were rotten. My neighbor’s son is another and other little boy. What was he doing? I think she was giving the baby the bottle at night – messed up his teeth.

R: Ohhh.

M: So Tiffany mentioned something important, about how well you take care of teeth

R: Don’t give them no bottles to put them to sleep, and don’t give them no bottles and leave them with it.

M: Right, the milk or juice ends up sitting on the teeth for a long time and thats not good.

R: That’s so sad. So young.

M: That happens with a lot with children. You’d be surprised. Sometimes young children have plaque and gingivitis. And you would think they’re so young. Why? Why does that happen? But it’s important even for little ones to make sure that they have good oral health.

(multiple voices)

R: Teach them to brush at an early age.

M: Yes. What risky behaviors do you think may cause poor oral health, aside from not brushing or flossing? How about smoking?

Rs: Yes.

M: And Adrianne also mentioned drinking. What else?

R: Soda, yeah.

R: You just not taking care of yourself. Not going to the doctor when you get a little pain you ignore it. Like (unintelligible) had a lady, she kept ignoring this pain. She died on the outside. You know what I’m talking about. On the outside of your building. She kept getting – she got arm pain at first. And she always told me her arm ache, and I say you better go see about it. She got that one massive heart attack and it took her away.

M: Wow, it’s important to seek medical care when you feel that something is not right with your body. Ok, anyone else? What risky behaviors do you think may cause poor oral health? How about sexual activity? We talked about HPV earlier? Infection with the human papilloma virus, can lead to cancer in the mouth and throat. Do you feel that it’s important to eat a healthy diet in order to maintain good oral health?

Rs: Yes.

M: Okay, so we have consensus on that. What kinds of foods do you feel may increase the risk of having poor oral health? We talked about sodas and we talked about candies. Any other ideas?

R: Greasy food.

M: Okay.

R: Low in calories, low in calories.

M: So low calorie food you think increase the risk?

R: No. Yes, like for your health, it’s better to eat low in calories.

M: Oh, ok, you’re saying that eating low calorie foods are good for your health in general. Not that low calorie foods are bad for oral health. Ok, now, for poor oral health, sugar-sweetened beverages, like sodas and juices can be bad for your oral health.

Rs: Yes. Right. Candies and gum.

M: Gum specifically that has sugar. Sugar-free gum is okay.

R: Right.

M: The thing is that you don’t want that sugar and acid to sit on your teeth. With juice, I’m not saying it’s bad to drink juice, but you don’t want to let it sit on your teeth for long periods of time. So after you drink juice, it would be good to rinse your mouth out with water or brush when you can. Because having sugar and acid sitting on your teeth will wear down the enamel over time and damage teeth. And it can happen to any of us. So it’s important to get that sugar off your teeth. The other thing – sticky foods, it’s important to clean teeth after sticky foods.

R: Sometimes pork is good for you, and sometimes it’s bad. It depends on how you cook it and what you put on it.

R: That makes sense.

M: Ok. So we have to watch our health in general. We talked about the foods and beverages that can lead to poor oral health. What kind of foods or beverages do you think may help you to have good oral health? Adrianne?

R: Vegetables. Salads. I fix a lot of salads.

M: Okay, very good. Maria?

R: Baked chicken.

M: So baked chicken, so lean protein. Chicken and especially chick breasts.

R: But you get so tired of it so easy.

M: I understand that. Sometimes you want to switch it up.

Rs: Yeah, fried chicken. Steak.

R: A mess of eggs and potatoes.

R: They do have oil with that, with the heart on it that’s low in cholesterol. Basically what you have to fight is that cholesterol.

M: Now, a big thing that we missed –

R: Whole grains.

M: Whole grains are good

R: And I see something else that’s good and it’s bad sometimes. It’s yogurt.

M: Well, yogurt has protein, calcium, and is good for the digestive system.

R: But it’s not good for diabetics.

M: That’s the thing, because many yogurts have a good a lot of sugar. So you have to know your own situation and how much sugar you’re able to have at that time. There are yogurts that are lower in sugar. But generally speaking, yogurt is good for you

R: Yeah, I love yogurt.

R: I don’t like it.

M: One of the most important things that we didn’t mention, Water. Water is very important…for oral health and health overall.

Rs: (Multiple agree).

**Barriers to Oral Health**

M: So let me ask you, are you afraid to go to the dentist?

Rs: No.

M: Okay. That’s very good.

R: I don’t like it, but I go.

R: It’s just like anything else. You’re tired of going to the doctor, but you got to go.

M: Yes, you have to do what is best for your health

R: That’s true.

R: I love going to the doctor and the dentist.

M: You do? That’s good.

M: Now let’s touch on another topic. Do you find it to be easy or difficult to find transportation to get to dental appointments?

R: No.

R: It depends on your insurance.

M: Ok, Susan says it depends on what’s on your insurance. So Liz, does your insurance provide transportation?

R: Um hmm. Medicaid pays for this. I’m not scared yet from riding buses and subways. I’ll get on them and go where I want to go.

R: Me, too. I was on one yesterday.

R: And I’ll ride that $5 taxi, too.

R: Amen, I do too.

R: They got Medicaid now, and Medicaid pay for all that, too.

M: So in general I think most of us are saying it’s not difficult to get to a dental appointment.

R: No. They have transportation for the insurance card, too. You just have to call and find out.

M: Okay

R: We can get on a Metro van

M: Metro access?

R: Yeah, that works for Metro. They pay you $10.

R: I have everything. I ride free. All my appointments, and everything.

M: That brings us back to the point of knowing what your health insurance covers. It’s important to know that.

M: Have you received any information about oral health or preventative care that was connected to other issues, like smoking cessation, dietary counseling, fluoride treatments?

R: Liz. Yes. All this is in the pamphlet that I get in the mail once a year.

M: So Liz, yes. Karen, no. Maria – how about you?

R: Yes.

M: Donna?

R: Yes.

M: Tiffany?

R: Yes.

M: Okay, so everyone has received this information. Now do you like to receive information about health? Do you like to receive it, for instance, by television, internet, radio, email?

R: Email.

R: I like to hear it from my doctor’s mouth. I don’t need no email. I want to hear it out of his mouth, in my presence.

M: Okay. So both Liz and Karen said they want to hear it from the doctor.

R: Yeah.

R: From my doctor.

M: Tiffany by a doctor. Ellen? Through the mail?

R: Yes.

M: Donna from a doctor.

R: I like it from the TV.

M: Okay, so Sarah likes to get it from the TV. How about you, Susan? How do you like to receive health information?

R: I receive a lot of it, and a lot of it…

R: I like to receive it other ways, too. Sometimes I just listen to Dr. Oz…

(multiple voices)

R: What he’s saying is not true, and that’s why they took him off the air.

M: Of all the different ways of receiving information, whether it’s the TV, internet, email, text messages on your cell phone, of all these different ways of information, which do you prefer to get it by? Which do you feel is the best for reaching you?

R: I agree with her.

R: I want it out of the mouth.

R: Because it’s a lot of false advertising. You can’t believe everything you hear.

M: Ok

R: I want to hear it out your mouth.

M: So Susan brought up an interesting point, too. You said you can’t believe everything that you hear.

R: No, you can’t.

M: There can be false advertising. So you want to make sure that the source is a reputable source that you can trust. Okay. Let me ask you, have you seen or heard any messages about oral health in the media?

R: No (multiple).

R: This is Karen. When they’re talking about it on television, oral health, and all of that, I don’t usually pay much attention to all of that stuff that they’re talking about. In fact, I don’t pay too much attention to anything – medical stuff that they’re talking about on television or the paper or whatever.

M: So Susan, you agree with Karen. You don’t pay attention to the media?

R: Because they trying to sell theyself.

Rs: (multiple agree)

R: They’re trying to sell lies.

M: Does anyone else have anything to add to this, with the messages that you hear on TV about oral health or even health in general. Do you pay attention to them or do you ignore them?

R: What, on TV?

M: Yes.

R: I ignore the TV, but I pay attention to what my doctor tells me.

M: Okay. And so what would you say is the reason why – for those who do ignore the messages – why do you ignore them?

R: I’m Adrianne. Like Miss Susan said, they’re selling themselves, and they’ll tell you anything on TV to try to get you to try that pill or buy that bottled medicine. So I don’t pay attention to the TV at all.

R: They’ve got everything on TV, my medication. They want you to buy.

R: And I want to tell you something. You don’t listen to your friends, either.

(unintelligible)

M: So what I’m hearing is that for those who don’t pay attention to them, you’re not sure if you can trust the message, that’s what I’m hearing.

Rs: Right. No trust.

M: So you want to have that trust.

R: Exactly.

M: Can you tell me of any messages that you’ve seen or heard that have helped you or encouraged you to maintain good oral health?

R: It’s just that I know that I should do it. I’ve never heard anything specific that would tell me that, but I know that I’m supposed to.

M: Okay.

R: They talk to you all of your life.

R: I was going to say why you should listen to your doctor, for two reasons. They run tests on you, they do things that help you improve. Like I had to go get a kidney test, so was I going to be a darned fool and not go?

Rs: Exactly. Right. That’s good.

R: So I went.

M: Yes, because you have to take care of yourself.

Rs: Right.

M: Not going doesn’t change a situation. If anything, it will make things worse because if there’s a problem that’s not being taken care of.

M: So what I’m hearing is that you like to receive those health messages, from your doctor or another trusted resource, and the doctor, from what I’m hearing, is the most trusted resource. So you want to hear more of this from your doctor. That’s what I’m getting.

Rs: Yes ma’am. (several respond yes)

M: Well, I thank you guys so much for your time.

R: I enjoyed it. (multiple)

M: I enjoyed it, too and I learned a lot. Thank you

R: I learned a lot, too. And I’m glad I came and I hope to see you all again.

M: We’re going to let Karen tell us something really quickly.

R: You know, I’ve been going over to Providence because I’m having a problem with a fear that I can’t get off the curbs and get on escalators or anything now, so they’re sending me over to Providence. So I saw this doctor, he’s a psychiatrist, that’s what they said. And I went over there and the first time I went he gave me a pill, an anxiety pill, to combat this fear that I have. So when I got the pill from the drugstore and I took it, I took it about two or three nights, and then I woke up one night, I was falling in a deep hole, a deep black hole. I took it for about three or four nights and each night I would wake up, two or three times, falling in this hole, and it scared me to death. So then I stopped for a while, and my daughter told me, she said, well, try it one more time and see if it happens. So I went on and took it again for about three more times, and then it got in my system. That time, when I went to sleep, when I woke up worms was crawling all over me. I jumped up out of the bed, turned the lights on – I pulled the covers off everything.

R: That was side effects of the medication.

R: And this was an anxiety pill. So she called him, and he told her to tell me not to take any more, which I wasn’t going to take it. But it was such a scary experience. My daughter said, you know, he told you there were going to be some side effects. But he said he’d never heard of side effects that I was having. It was a scary experience.

M: If you experience side effects, please see your doctor or go to the emergency room right away. Before I close, I want to know, would you be interested in receiving any other health information from us?

Rs: Sure. Anything we can get. Yes. I need that information to go out.

M: I thank you so much for coming out to talk with us. Your opinions and this discussion mean a lot to us.

// end of recording //

Jill
